# The Tungsten-Promoted Synthesis of Piperidyl-Modified *erythro*-Methylphenidate Derivatives

**DOI:** 10.1021/acscentsci.3c00556

**Published:** 2023-08-30

**Authors:** Jonathan D. Dabbs, Megan N. Ericson, Justin H. Wilde, Rachel F. Lombardo, Earl C. Ashcraft, Diane A. Dickie, W. Dean Harman

**Affiliations:** Department of Chemistry, University of Virginia, Charlottesville, Virginia 22904, United States

## Abstract

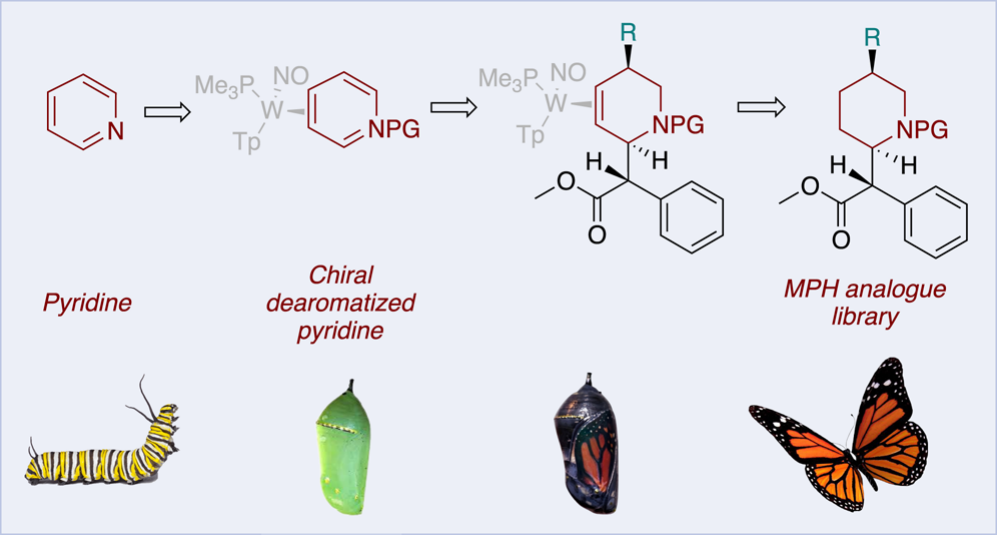

Due to its efficacy as a dopamine receptor agonist, methylphenidate (MPH) is of interest as a potential therapeutic for cocaine addiction. While numerous derivatives of MPH have been investigated for their potential medicinal value, functionalization of the piperidine ring has not been explored. The pyridine borane ligand in WTp(NO)(PMe_3_)(η^2^-pyBH_3_) is dearomatized by the metal and can be elaborated to the analogous η^2^-mesylpyridinium complex. Installing a methyl phenylacetate moiety at the C2′ position via a Reformatsky reaction followed by a tandem protonation/nucleophilic addition sequence results in a library of *erythro* MPH analogues functionalized at the piperidyl C5′ position. The functional group is added chemoselectively to C5′, *cis* to the methyl phenylacetate. Repeating this procedure with an enantioenriched source of the tungsten reagent results in enantioenriched MPH derivatives. All identities of the newly reported compounds are supported by comprehensive 2D NMR and HRMS data or crystallographic data.

## Abstract 2

Pyridine η^2^-bound to a tungsten fragment enables its transformation to a highly functionalized tetrahydropyridine. This method was utilized to develop a library of piperidyl-containing drug analogues.

## Introduction

**Figure 1:**
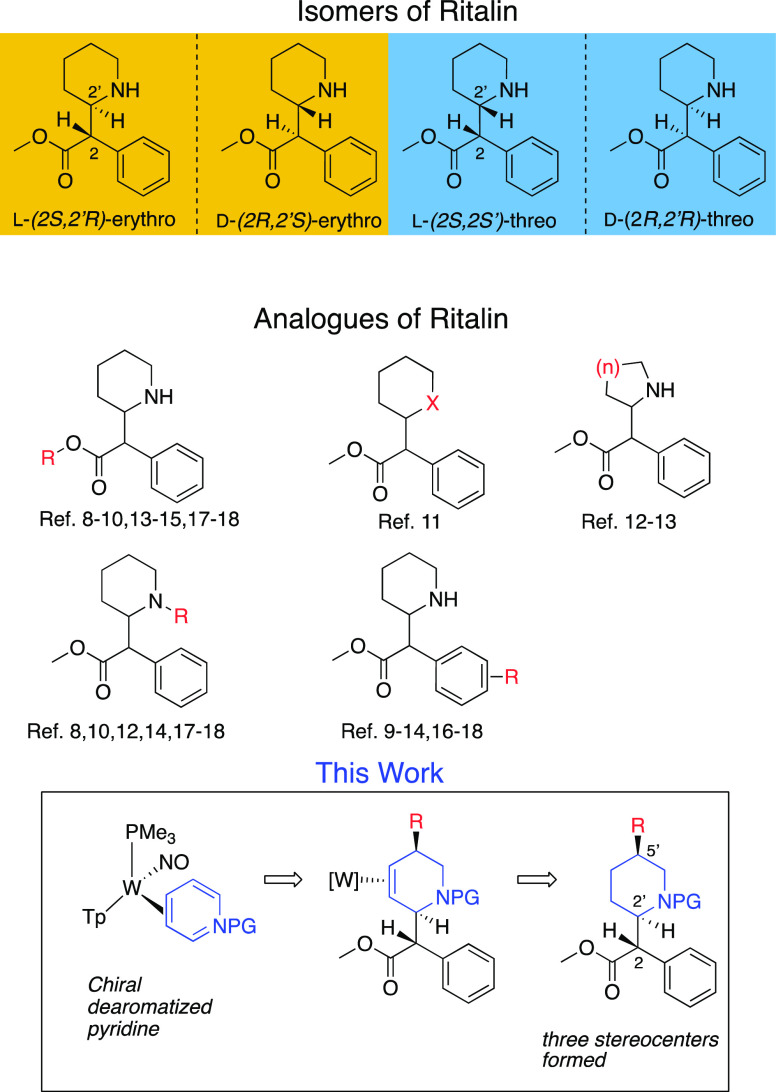
Two pairs of MPH diastereomers, previously reported analogues of MPH, and a proposed dearomatization approach to C5′-substituted MPH analogs.

The Center for Disease Control reported over 100,000 deaths in the United States due to overdoses in 2021, about a quarter of which were attributed to cocaine.^1^ Small-molecule therapeutics have proven to be effective treatments for various drug addictions. Heroin addiction, for example, is a disorder most commonly treated with methadone, which prevents drug withdrawal symptoms while being less addictive than the original drug.^2,3^ While pharmacotherapies exist for heroin and other opioids, effective therapeutics are lacking for cocaine addiction.^4^ Methylphenidate (MPH), commercially known as Ritalin and prescribed extensively for treating attention deficit hyperactivity disorder (ADHD), behaves similarly to cocaine as a dopamine receptor agonist.^5,6^ It is typically administered as a mixture of isomers, which consists of two diastereomers and their respective enantiomers (Figure 1). Numerous research groups have explored MPH analogues in hopes of identifying a compound that prevents cocaine from binding to the dopamine transporter (DAT) while allowing for the reuptake of dopamine.^7^ Most of these studies confine the areas of structural diversity on the MPH scaffold to the ester, phenyl ring, piperidine nitrogen, or the heterocycle ring-size (Figure 1).^8−18^ However, there appear to be no studies that incorporate functionality on the piperidine ring, largely owing to the lack of established general synthetic methods for such compounds. Herein we demonstrate an organometallic-based approach for synthesizing *erythro*-MPH analogues in which functionalized piperidyl variants are stereoselectively prepared from a chiral η^2^-pyridine complex (Figure 1).

**Figure 2:**
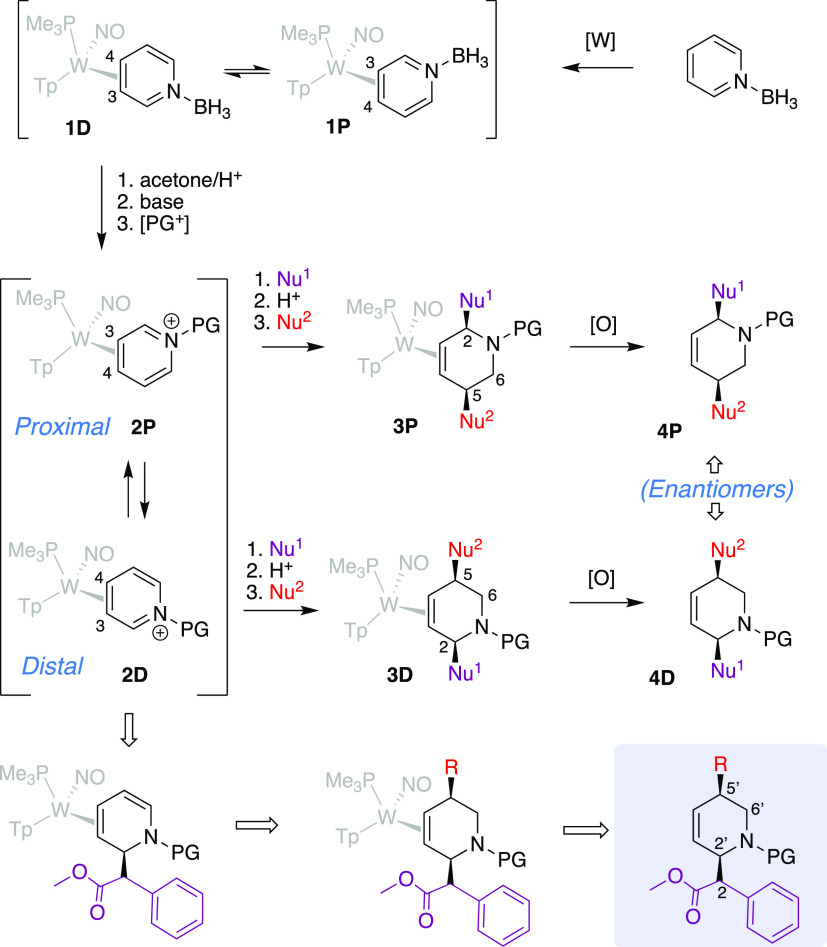
Tungsten-promoted dearomatization of pyridines and its proposed application to MPH analogues.

Previous work in our laboratory has demonstrated that highly functionalized tetrahydropyridines (THPs) can be synthesized stereoselectively from pyridine borane through its dihapto-coordination to {WTp(NO)(PMe_3_)} (Figure 2; Tp = hydrotris(pyrazolyl)borate).^19,20^ The tungsten binds to C3 and C4 of the pyridine on either face of the ring, resulting in two “coordination diastereomers” (**1D**, **1P**). Their equilibrium ratio (cdr) is 3:1 favoring **1D**, with the nitrogen distal to the PMe_3_. This complex can be converted to the pyridinium (pyH^+^) analogue via acetone and acid and then deprotonated in the presence of a protecting group (typically in the form of an anhydride), through which an array of protecting groups (PG^+^) can be added to the nitrogen (**2P**, **2D**; Figure 2). From this point, a range of different nucleophiles (Nu^1–^) can be added to C2 to generate dihydropyridine (DHP) complexes, which in turn can be protonated at C6′ of the MPH core and then treated with a second nucleophile (Nu^2–^) at C5′ to form THP complexes **3P** and **3D**. Oxidative decomplexation reveals *cis*-disubstituted THP products **4P** and **4D**. The two THP coordination diastereomers led to the same organic product under racemic conditions (**4P** = **4D**). However, if a single configuration of the tungsten complex was to be used, **4P** and **4D** would be enantiomers. Therefore, a high cdr of the pyridinium complex is essential if enantioenriched organic products are desired. Electron-withdrawing PGs have been shown to improve the cdr owing to a thermodynamic preference of the complex to orient electron-deficient allylic carbons distal to the trimethylphosphine ligand.^21^ They also render the C2 carbon more electrophilic, providing a broader range of possible nucleophiles. Our proposed strategy in preparing MPH derivatives was to install the methyl phenylacetate portion at C2 of a distal η^2^-pyridine complex via a Reformatsky reaction, followed by elaboration of the resulting DHP to a *cis*-disubstituted THP or piperidine.

## Results and Discussion

**Figure 3:**
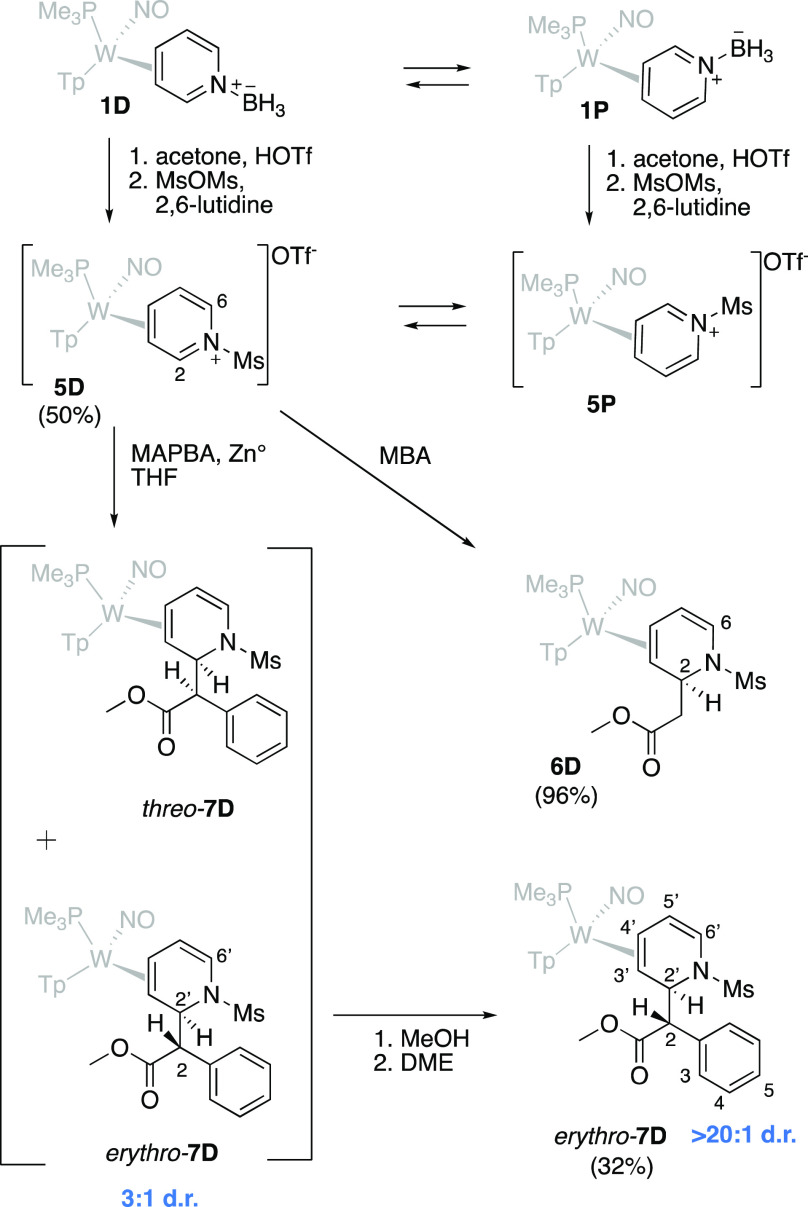
Synthesis of a key DHP precursor (*erythro*-**7D**) to piperidine analogs of MPH.

Previous studies exploring the tungsten-promoted dearomatization of pyridine utilized an acetyl protecting group (PG = Ac; Figure 2), where the corresponding pyridinium complex could be prepared in up to a 10:1 cdr mixture.^22−24^ The *N*-acetyl-2-ethyl acetate-1,2-dihydropyridine complex **2D** was synthesized via a Reformatsky reaction between the corresponding pyridinium complex (**1**), methyl bromoacetate (MBA), and zinc.^25^ Owing to the position of the metal, the addition cleanly occurs at C2, with no sign of competing C4 addition.^26^ It was postulated that a similar strategy could be utilized to install the methyl phenylacetate moiety to the pyridinium core. However, the acetyl protecting group resulted in rotational isomers (∼1:1) for all downstream complexes for this system due to hindered acetyl rotation,^27^ and the Reformatsky reaction was not stereoselective (*erythro*: *threo* ratio: 1:1). Because comprehensive NMR analysis of these ∼1:1:1:1 isomeric mixtures proved to be challenging, an alternative protecting group was sought. Given the broad use of sulfonamides in drug design,^28^ we settled on a methanesulfonyl (mesyl or Ms) group to protect and activate the nitrogen. The reaction of mesyl anhydride in the presence of **1** and lutidine forms the new pyridinium complex **5**, and stirring the solution in an oil bath at 60 °C enriches the cdr from 3:1 to 10:1 as tracked by ^31^P NMR. After the mixture was subjected to aqueous washes, coordination diastereomers **5D** and **5P** were precipitated in stirring diethyl ether. To our delight, triturating this powder mixture in ethyl acetate further enriched the cdr to >20:1 (Figure 3). Furthermore, any decomposition impurities are removed during this trituration, resulting in consistently clean and highly enriched material on a multigram scale.

### Synthesis of the Key DHP Complex

To ensure that the mesyl-substituted pyridinium complex (**5D**) behaved in a manner similar to the acetyl derivative,^25^ a Reformatsky reaction with MBA was performed on **5D** resulting in the DHP complex **6D** (Figure 3). The structure was confirmed by 2D NMR analysis and single-crystal X-ray diffraction (SC-XRD). Under similar reaction conditions, **5D** smoothly undergoes a Reformatsky addition with methyl α-phenyl bromoacetate (MAPBA) to produce a DHP complex (**7D**). Incorporation of the methyl phenyl ester moiety occurs exclusively *anti* to the metal at C2 of the pyridine ring. However, the product is formed as a 3:1 *erythro*:*threo* mixture of diastereomers. We found that trituration in methanol followed by trituration in dimethoxyethane (DME) enriches the dr from 3:1 to >20:1. Whereas methanol trituration removes decomposition impurities, isomeric enrichment occurs during the DME trituration (Supporting Information).

**Figure 4:**
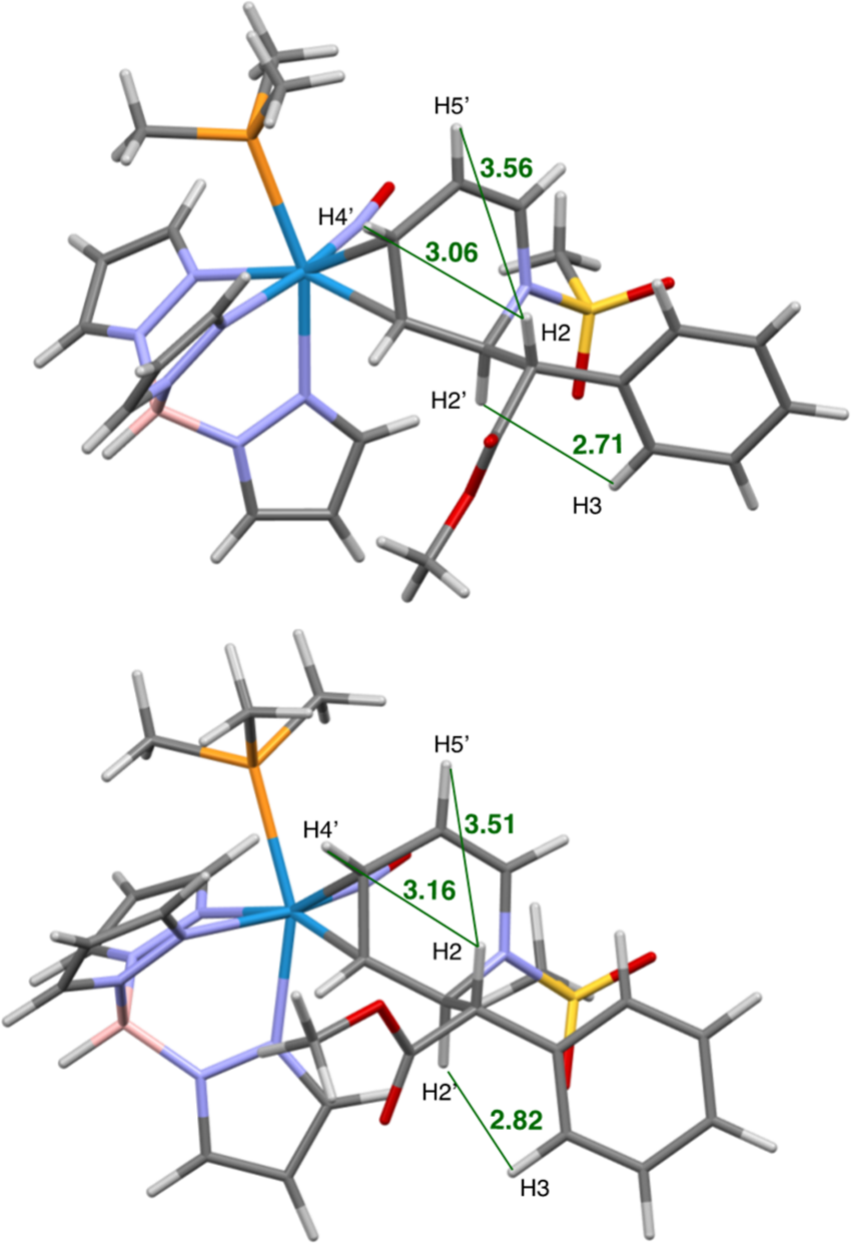
Molecular structure determination of *erythro*-**7D** from SC-XRD (top) and DFT calculation (bottom).

SC-XRD data were obtained for the *erythro* diastereomer (*erythro-***7D**; Figure 4); however, to confirm the stereochemistry of the bulk material, NOE data were considered along with a DFT analysis. Calculations were performed using the M06 functional alongside a split basis set with LANL2DZ applied to tungsten and 6-31G(d,p) on all main group atoms for optimizations to the lowest energy conformations of the two diastereomers (Supporting Information). Hydrogen–hydrogen distances were determined and compared to experimental nuclear Overhauser effect spectroscopy (NOESY) data of a 3:1 dr sample, and several unique interactions were identified. The distance between H2 and H9 was 2.82 Å in the *erythro* diastereomer and 4.34 Å in the *threo* complex. Additionally, the distances between H7 and H4/H5 were 3.16 Å/3.51 Å in the *erythro* isomer and 4.10 Å/5.34 for *threo*. NOESY spatial interactions were present between H2′-H3, H2-H4′, and H2-H5′ in the major complex but absent in the minor complex (Supporting Information). Collectively, these observations support the hypothesis that the *erythro* diastereomer (*erythro-***7D**) is the major complex, while the *threo* (*threo-***7D**) diastereomer is the minor. The addition of the methyl phenyl ester moiety can be performed on a multigram scale (overall yield of 22% from **1D**; 2–3 g), resulting in a pure *erythro* methyl phenyl ester DHP complex (*erythro-***7D**; dr > 20:1) without chromatography. We note that, while the *threo* isomer of MPH is most biologically active,^5,6^ this is not necessarily the case for its derivatives,^8−18^ so we continued our study with the high purity *erythro* isomer **7D**.

### Preparing a Library of THP Complexes

**Figure 5:**
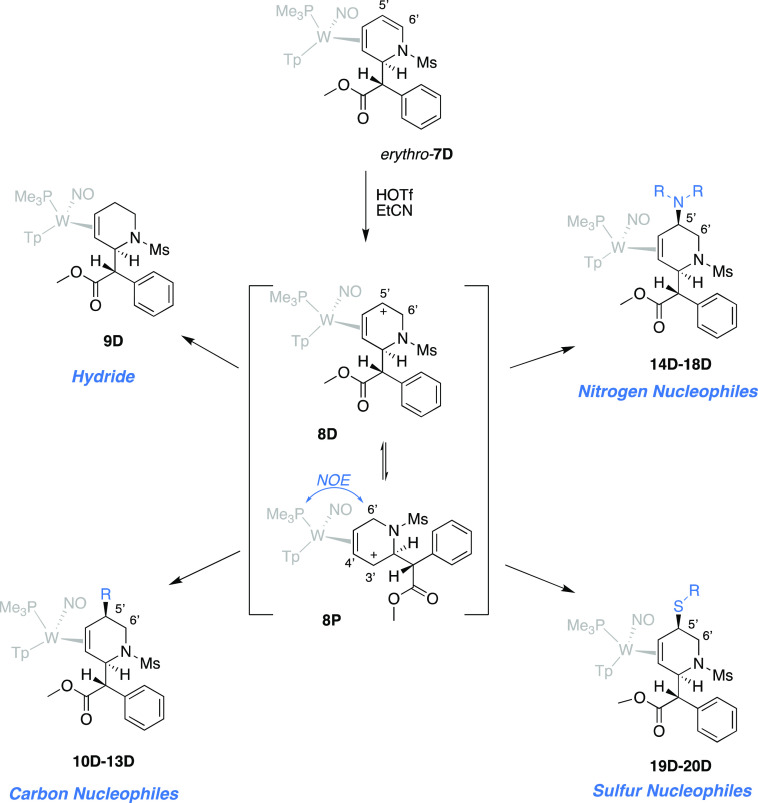
Synthesis of THP precursors to MPH derivatives.

**Figure 6:**
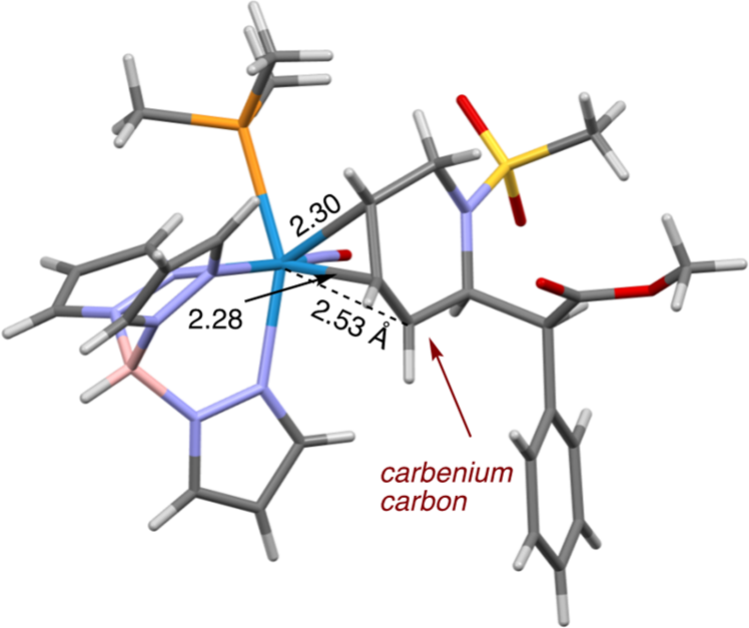
Molecular structure determination for η^2^-allyl complex **8P** showing carbenium carbon distal to the PMe_3_.

*Erythro*-**7D** could be protonated at C6′ by triflic acid in propionitrile to form a single isomer of the allyl complex (**8P**Figure 5). We note here that tungsten backbonding directs the proton to C6′, rather than C5′, the expected site of protonation for an organic enamide.^22^ Precipitation was then induced by adding the solution to stirring ether to form a readily isolable powder. Previous studies have demonstrated how π-allyl complexes of {WTp(NO)(PMe_3_)} are hyperdistorted.^21^ They are described herein as dihapto-coordinated with the central allylic carbon and one terminal carbon tightly bound to the tungsten and the other terminally bound carbon loosely coordinated (the carbenium-like carbon). DFT calculations indicate that for η^2^-allyl complexes of {WTp(NO)(PMe_3_)}^21^ there is a significant thermodynamic preference for placing the carbenium carbon distal to the trimethylphosphine ligand. Accordingly, NOESY shows a prominent interaction between the C6′ methylene protons and trimethylphosphine ligand, indicating that **8P** is the thermodynamically favored allyl conformer. This is further supported by SC-XRD, which revealed that **8P** is the conformation present in the solid state (Figure 6). Yet nucleophilic attack is observed to occur at C5′ (vide infra), indicating an allyl conformational shift to **8D** prior to nucleophilic addition.

With the goal of demonstrating modular diversity that would be amenable to forming combinatorial libraries, we explored the reactivity of allyl complex **8D** with various nucleophiles. *cis*-5-Substituted-2-(methyl phenylester)-1,2,5,6-tetrahydropyridine (THP) complexes (Figure 5) were formed in solution and then precipitated in stirring chilled hexanes or pentanes. The nucleophiles in Table 1 were chosen to exemplify the broad compatibility of the metal complex with various classes of nucleophile and to demonstrate the potential of this methodology to directly access a wide range of functional groups. These nucleophiles, which include hydride, cyanide, enolates, Grignard reagents, amines, amides, imides, and thiols, can be added to isolated allyl complex **8D** or added in a one-pot reaction following the protonation of *erythro*-**7D**. Despite the preference of the carbenium to be distal to the PMe_3_ (**8P**), all of these nucleophiles add to C5 exclusively via the minor conformer of the allyl species (**8D**). In all cases, there is a high stereochemical preference for the addition *anti* to the metal, as is indicated by SC-XRD and NOESY data (Supporting Information).

**Table 1:**
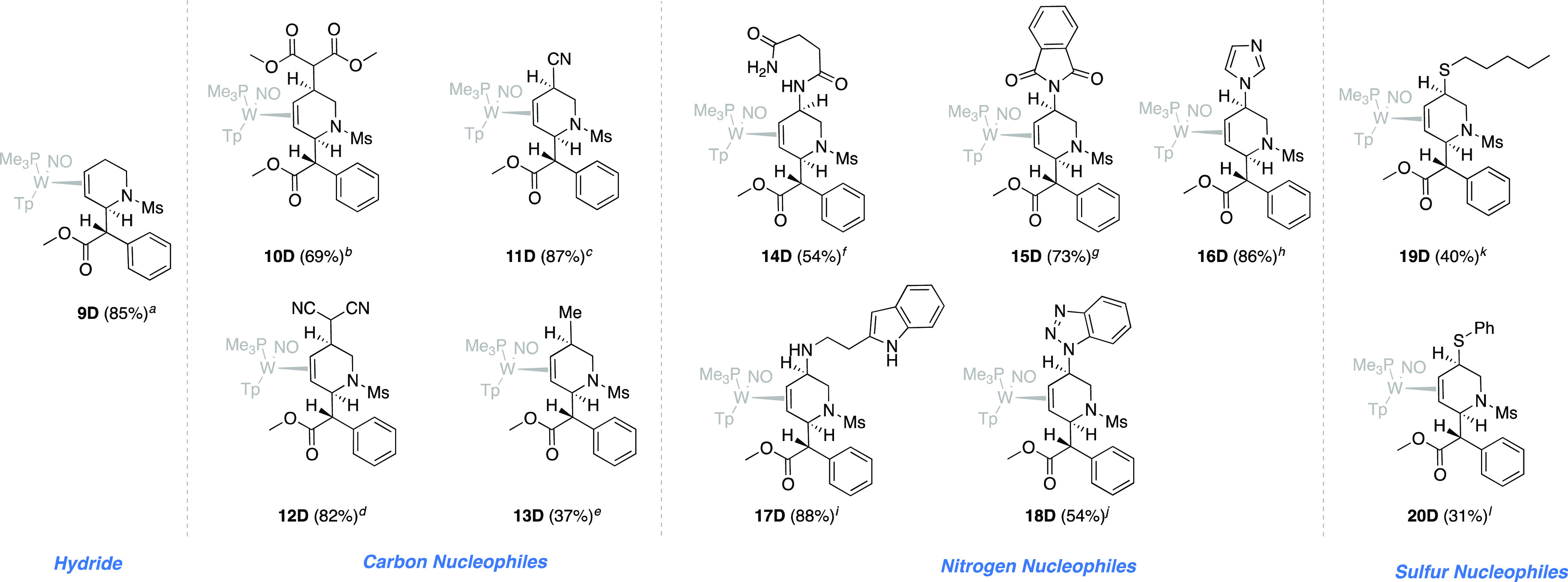
THP Precursors to MPH Analogs with C5 Functionalization of the Piperidine Ring

a Step 1: **7D**, HOTf, 25 °C; Step 2: NaCNBH_3_, −50 °C.

b Step 1: **7D**, HOTf, 25 °C; Step 2: LiCH(CO_2_Me)_2_, −10 °C.

c Step 1: **7D**, HOTf, 25 °C; Step 2: NaCN, −40 °C.

d Step 1: **7D**, HOTf, 25 °C; Step 2: (CN)_2_CH_2_, KOtBu, 0 °C.

e **8**, MeMgBr, −60 °C.

f **8**, succinamide, nBuLi, −60 °C.

g Step 1: **7D**, HOTf, 25 °C; Step 2: phthalimide, KOtBu, −60 °C.

h Step 1: **7D**, HOTf, 25 °C; Step 2: imidazole, KOtBu, −30 °C.

i Step 1: **7D**, HOTf, 25 °C; Step 2: tryptamine, KOtBu, −50 °C.

j Step 1: **7D**, HOTf, 25 °C; Step 2: benzotriazole, KOtBu, −40 °C.

k Step 1: **7D**, HOTf, 25 °C; Step 2: pentanethiol, KOtBu, −50 °C.

l Step 1: **7D**, HOTf, 25 °C; Step 2: thiophenol, KOtBu, −40 °C.

While the parent THP complex (**9D**) was synthesized via the addition of sodium cyanoborohydride at −50 °C, the addition of **8** to a lithium dimethyl malonate solution provided **10D**. Similarly, an allyl **8** solution was added to a cooled methanol solution (−50 °C) containing sodium cyanide to form **11D**. Malononitrile was also added successfully (**12D**) by combining cooled solutions of allyl **8** and deprotonated malononitrile. Grignard reagents are also compatible nucleophiles: Successful addition of MeMgBr occurs after reacting chilled reactants at −60 °C for 60 h to form **13D**. Both aliphatic amines and amides can be incorporated, as well as N-heterocycles: Tryptamine addition (addition at primary amine) was achieved by combining it with potassium *tert*-butoxide and a solution of **8** to form **17D**. Similarly, **14D** was prepared by adding a solution of **8** to a solution of succinamide and base. Benzotriazole was treated with potassium *t*-butoxide and cooled before the addition of allyl complex **8**, to form **18D**. Phthalimide (**15D**) and imidazole (**16D**) followed a similar reaction course. Finally, thiol additions were accomplished using pentanethiol (**19D**) or thiophenol (**20D**) and base. Despite the presence of strong acid and base in these reactions, the diastereomeric enrichment of these compounds is retained throughout the syntheses, resulting in THP complexes with diastereomeric ratios of >20:1. THP complexes are produced in yields ranging from 31 to 88% and were characterized by multiple spectroscopic techniques including ^1^H NMR, ^13^C NMR, 2D-NMR, and IR, as well as being characterized by SC-XRD, HRMS, and cyclic voltammetry.

### Releasing the THP from the Metal

**Figure 7:**
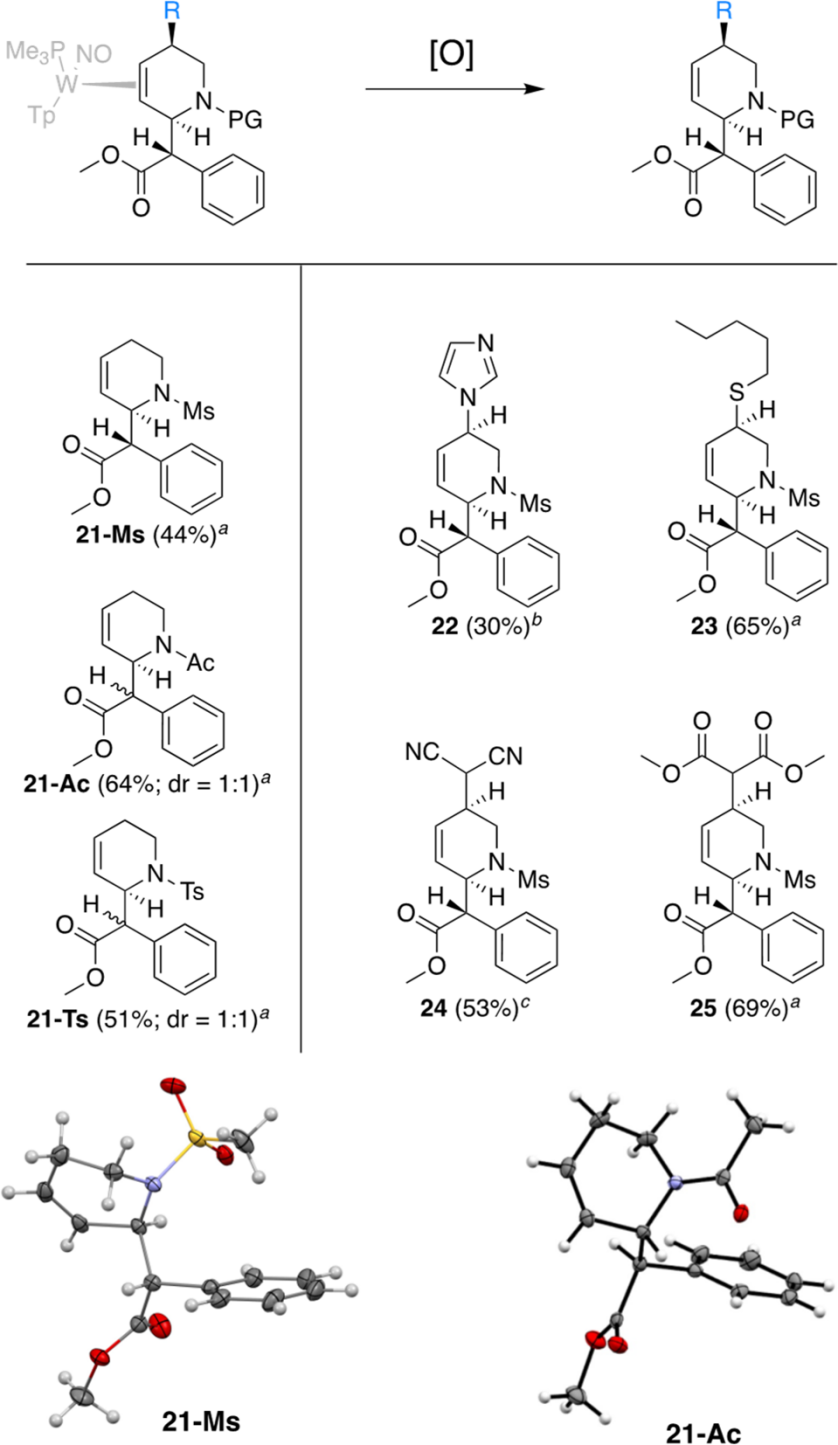
Oxidative decomplexation of THP compounds **21**–**25**. ^*a*^DDQ, 25 °C. ^*b*^DDQ, HOTf, −40 °C. ^*c*^CAN, 25 °C.

The functionalized THPs can be liberated by oxidation of the metal center. As an example, **9D** undergoes chemical oxidation with four equivalents of DDQ in acetone, and subsequent chromatography results in purified THP organic (**21**). Incomplete oxidation was observed via ^1^H NMR when less than four equivalents were used. We attribute this to metal decomposition products undergoing multiple oxidation events, thus necessitating a greater amount of oxidant. Similarly, **22**–**25** were also oxidatively liberated via DDQ. All organics (Figure 7) were recovered through extraction or purified via chromatography. Similar procedures to those described above provided routes to both *N*-tosylated and *N*-acetylated variations of THP MPH derivatives (Supporting Information). As with the mesylated analogs, the Reformatsky reaction occurred with low stereoselectivity. Two examples of the parent methyl phenyl acetate THP (**21-Ac** and **21-Ts**) were prepared and are included in Figure 7 for comparison.^25,26^

### Hydrogenation of the THP to Piperidine

**Figure 8:**
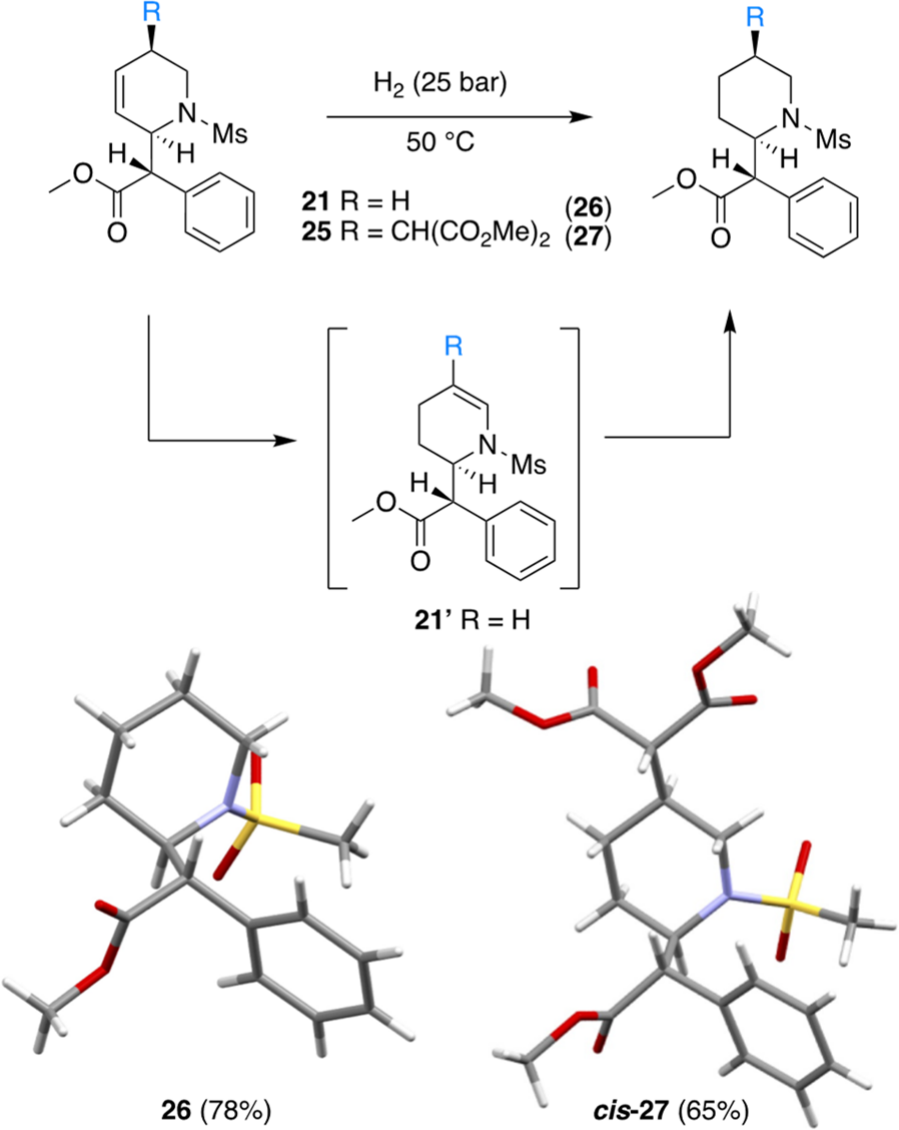
Hydrogenations of MPH and THP organics were performed to form the corresponding *erythro* MPH analogue. The presence of the trans isomer is attributed to an observed enamine intermediate formed from a Pd-promoted alkene isomerization.

As a demonstration of the ability to generate the corresponding piperidines from THPs such as those reported in Figure 7, THP **21-Ms** was reduced to its saturated analogue (**26**) through hydrogenation. Using 5% Pd on activated carbon catalyst, the hydrogenation of **21-Ms** was carried out at 50 °C under 25 bar (∼360 psi) of H_2_ (Figure 8). Complete conversion to the reduced form (**26**) occurred after 5 h. However, prematurely arresting the reaction revealed the presence of an intermediate (**21′**; Supporting Information). 2D NMR analysis indicated that this compound was likely a constitutional isomer of THP **21-Ms** in which the alkene has migrated to form the “enamide” **21′** (Figure 8). Similar palladium-catalyzed isomerizations have been reported for other THPs and for a 3-pyrrolene, in which an enamide (2-pyrrolene) was formed.^29−31^ As anticipated, further reduction of the mixture of **26** and **21′** resulted in complete conversion to the piperidine **26**. The structure of **26** was confirmed via SC-XRD. Compound **25** was also hydrogenated to **27** under identical conditions as confirmed by SC-XRD. Of note, Ellman et al. have shown the value of 3,4-THPs as precursors to highly functionalized, oxygenated piperidines.^32^ Several procedures have been published for removal of the mesyl group from secondary amines, but we have not explored this aspect in any detail.^33−35^

### Enantioenriched THPs

**Figure 9:**
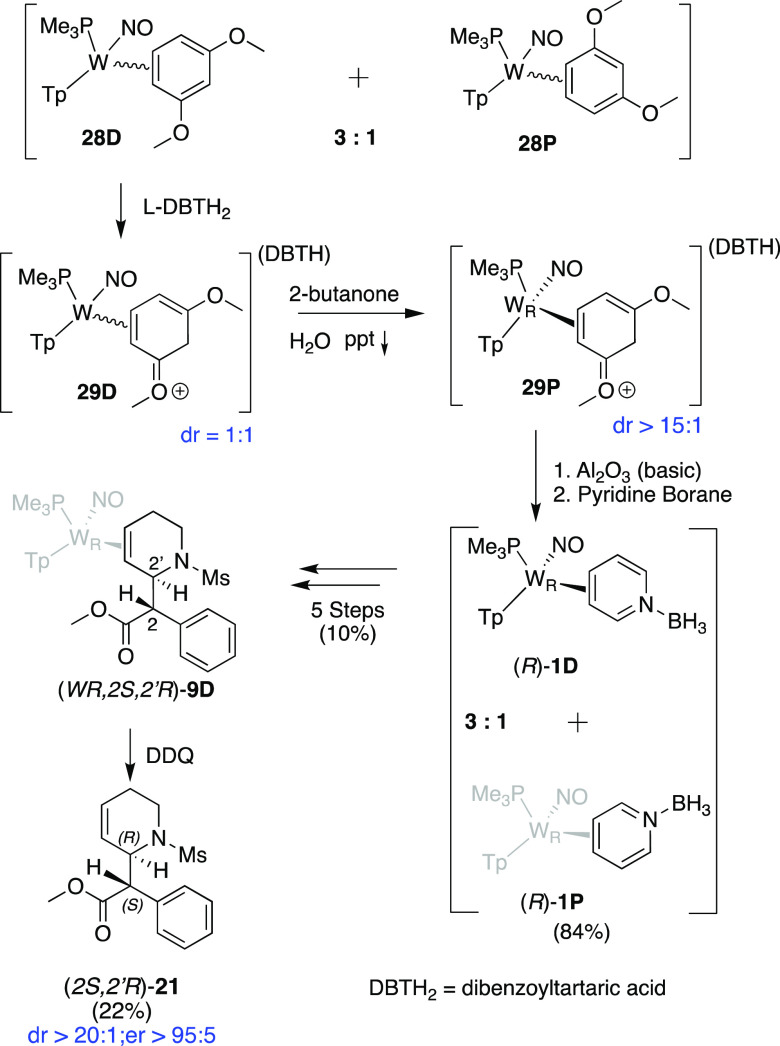
Preparation of enantioenriched MPH analog.

We next explored the possibility of using the methodology outlined above to obtain enantioenriched compounds. Previously, Lankenau et al. developed a general procedure for the resolution of WTp(NO)(PMe_3_)(η^2^-1,3-dimethoxybenzene) enantiomers,^36^ and this process has since been modified and optimized.^37^ The procedure resolves the *S* and *R* hands of the metal by protonating the corresponding racemic dimethoxybenzene complex with l-dibenzoyl tartaric acid (l-DBTH_2_) and then physically separating the resulting diastereomer salts based on their solubility differences. This robust approach allows for enriched samples of either hand on a gram scale, with ee’s from 80 to 95+%, and the dimethoxybenzene ligand can be replaced with other aromatic molecules (e.g., benzene, anisole) with complete retention of the metal stereocenter. Applying this technique to pyridines introduced several challenges. Synthesizing the pyridine borane **1** in an enantioenriched form required the deprotonation of [(*R*)-WTp(NO)(PMe_3_)(η^2^-dimethoxybenzenium)](L-DBTH) (**29D**; dr ≈ 10:1) to generate an exchange-labile precursor (**28**; Figure 9). Deprotonation was conducted with basic alumina (owing to its ease of separation) in THF, and (*R*)-WTp(NO)(PMe_3_)(η^2^-dimethoxybenzene) (**28**) was then precipitated with stirring hexanes. This compound was then combined with pyridine borane (30 equiv) and TEA (1.5 equiv) for 36 h. The workup for this reaction was identical to the previously published version.^19^ This enantioenriched (*R*)-**1** was then subjected to deprotection, mesylation, and Reformatsky addition (vide supra). However, the tandem methanol/DME triturations failed to clean and enrich the dr of (*WR,2S,2′R*)-**7D**, which, as in the racemic preparation, was present as a 3:1 mixture of isomers. This apparent difference in diastereomer solubilities was attributed to the enantioenriched **7D** crystallizing in a different space group than does racemic **7D**. The racemic material crystallizes from dimethoxyethane in the space group *P*1̅, which, being centrosymmetric, requires both hands of the complex to be present in equal amounts. We found that purifying (*WR,2S,2′R*)-**7D** via a basic alumina column and triturating the precipitated complex in ethanol improves the dr to >20:1. Clean (*WR,2S,2′R*)-**7D** was then protonated under conditions identical to the conditions used for the racemic mixture to form (*WR,2S,2’R*)-**8**. This compound was then converted to (*WR,2S,2′R*)-**9D** and the desired organic (*2S,2′R*)-**21** was then oxidatively liberated from the metal with DDQ. The er of the free organic was determined by chiral HPLC to be >95:5 (Supporting Information). Notably, this compound has the configuration at C2′ (*R*) that matches the desired d enantiomer of *threo*-MPH, and procedures have been developed by Novartis to convert (*2S,2′R*)-*erythro*-MPH to the desired (2*R,2′R*)-MPH (d-*threo*) in alkaline solution.^38^

Of the numerous derivatives of MPH that have appeared in the academic and patent literature,^8−18^ remarkably few have a substituent on a saturated piperidine ring. Of those few examples that have been reported, most are prepared from piperidines or piperidones via the C–H activation and addition of the phenylacetate group adjacent to the ring nitrogen by a rhodium catalyst.^39^ Although there do not appear to be examples of C5′-substituted MPH analogs prepared from this method, such an approach may be viable, but controlling stereochemistry would be challenging. It seems that the most general method for preparing such analogs would be from the hydrogenation of pyridine precursors. While hydrogenations of pyridines and pyridinium salts are well established^40−42^ and have been used to prepare the parent MPH,^43^ such an approach has not been applied to MPH analogs in which the piperidine was functionalized.

## Conclusions

This study describes a methodology capable of accessing piperidine-functionalized *N*-mesyl *erythro* methylphenidate analogues with high degrees of regioselectivity, stereoselectivity, and functional group tolerance. A Reformatsky reaction involving a {WTp(NO)(PMe_3_)} *N*-mesyl pyridinium complex and α-phenylmethyl bromoacetate yields an *erythro* methylphenidate DHP complex, which is enriched to a dr of >20:1 through triturations. After the formation of an allyl complex, the successful incorporation of nucleophiles to form tetrahydropyridine complexes with high diastereoselectivity includes cyanide, malononitrile, dimethyl malonate, Grignard reagents, amines, amides, imides, and thiols. This strategy provides access to a wide range of MPH analogues functionalized at the C5′-position on the piperidine ring that would be difficult to access via conventional methods. Furthermore, the ubiquity of the piperidine scaffold within small-molecule drugs underscores the potential utility of this system, which rapidly incorporates a broad range of functional groups and nucleophile types into piperidine cores. Efforts continue on developing a fully stereoselective Reformatsky reaction with an η^2^ pyridinium system that would directly lead to the *threo* diastereomer family of C5-substituted MPHs, which, at least for the parent, is the most biologically potent.^44^

## Supporting Information Available

^1^H and ^13^C NMR spectra of selected compounds (PDF-1)
crystallographic information for compounds **6D**, **7D**, **8**, **9D**, **13D**, **19D**, **21**-**Ms**, **21**-**Ac**, **22**, **26**, and **27**. CCDC 2256163–2256172 and 2260260 deposition numbers contain the supplementary crystallographic data for this paper. These data can be obtained free of charge from The Cambridge Crystallographic Data Centre via www.ccdc.cam.ac.uk/structures (CIF, PDF-2, PDF-3)
Transparent Peer Review report available (PDF)

## Supplementary Material

oc3c00556_si_001.pdf

oc3c00556_si_002.cif
