# Supplementary material for: The Tungsten-Promoted Synthesis of Piperidyl-Modified erythro-Methylphenidate Derivatives
Source: ACS Cent Sci. 2023 Aug 30;9(9):1775–83. doi: 10.1021/acscentsci.3c00556 (PMC10540299; doi:10.1021/acscentsci.3c00556)
Supplement: Supplementary file 1 — oc3c00556_si_001.pdf [file oc3c00556_si_001.pdf]

**Supporting Information**

# The Tungsten-Promoted Synthesis of Piperidyl-Modified *erythro*-Methylphenidate Derivatives

Jonathan D. Dabbs,[#,1] Megan N. Ericson,[#,1] Justin H. Wilde,[1] Rachel F. Lombardo,[1] Earl C. Ashcraft,[1] Diane A. Dickie,[1] and W. Dean Harman*[,1]

[1] *Department of Chemistry, University of Virginia, Charlottesville, Virginia 22904, United States*
# authors contributed equally
*Email: wdh5z@virginia.edu

**Table of Contents**

| | |
|---|---|
| **S1-S3** | **Supporting Information Title Page, Table of Contents** |
| **S4** | **General Methods** |
| **S5** | **Synthesis and Characterization of 5D** |
| **S6** | **Synthesis and Characterization of 6D** |
| **S7** | **Synthesis and Characterization of 7D** |
| **S8** | **Synthesis and Characterization of 8** |
| **S9** | **Synthesis and Characterization of 9D** |
| **S10** | **Synthesis and Characterization of 10D** |
| **S11** | **Synthesis and Characterization of 11D** |
| **S12** | **Synthesis and Characterization of 12D** |
| **S13** | **Synthesis and Characterization of 13D** |
| **S14** | **Synthesis and Characterization of 14D** |
| **S15** | **Synthesis and Characterization of 15D** |
| **S16** | **Synthesis and Characterization of 16D** |
| **S17** | **Synthesis and Characterization of 17D** |
| **S18** | **Synthesis and Characterization of 18D** |
| **S19** | **Synthesis and Characterization of 19D** |
| **S20** | **Synthesis and Characterization of 20D** |
| **S21** | **Synthesis and Characterization of 21-Ms** |
| **S22** | **Synthesis and Characterization of 21-Ac** |

| | |
|---|---|
| **S23** | **Synthesis and Characterization of 21-Ts** |
| **S24** | **Synthesis and Characterization of 22** |
| **S25** | **Synthesis and Characterization of 23** |
| **S26** | **Synthesis and Characterization of 24** |
| **S27** | **Synthesis and Characterization of 25** |
| **S28** | **Synthesis and Characterization of 26** |
| **S29** | **Synthesis and Characterization of 27** |
| **S30** | **Figures S1 and S2: $^{1}$H-NMRs of 5 and 5D** |
| **S31** | **Figure S3: $^{13}$C-NMR of 5** |
| **S32** | **Figures S4 and S5: $^{1}$H-NMR and $^{13}$C-NMR of 6D** |
| **S33** | **Figures S6 and S7: $^{1}$H-NMR and $^{13}$C-NMR of 7D** |
| **S34** | **Figures S8 and S9: $^{1}$H-NMR and $^{13}$C-NMR of *erythro*-7D** |
| **S35** | **Figures S10 and S11: $^{1}$H-NMR and $^{13}$C-NMR of 8** |
| **S36** | **Figures S12 and S13: $^{1}$H-NMR and $^{13}$C-NMR of 9D** |
| **S37** | **Figures S14 and S15: $^{1}$H-NMR and $^{13}$C-NMR of 10D** |
| **S38** | **Figures S16 and S17: $^{1}$H-NMR and $^{13}$C-NMR of 11D** |
| **S39** | **Figures S18 and S19: $^{1}$H-NMR and $^{13}$C-NMR of 12D** |
| **S40** | **Figures S20 and S21: $^{1}$H-NMR and $^{13}$C-NMR of 13D** |
| **S41** | **Figures S22 and S23: $^{1}$H-NMR and $^{13}$C-NMR of 14D** |
| **S42** | **Figures S24 and S25: $^{1}$H-NMR and $^{13}$C-NMR of 15D** |
| **S43** | **Figures S26 and S27: $^{1}$H-NMR and $^{13}$C-NMR of 16D** |
| **S44** | **Figures S28 and S29: $^{1}$H-NMR and $^{13}$C-NMR of 17D** |
| **S45** | **Figures S30 and S31: $^{1}$H-NMR and $^{13}$C-NMR of 18D** |
| **S46** | **Figures S32 and S33: $^{1}$H-NMR and $^{13}$C-NMR of 19D** |
| **S47** | **Figures S34 and S35: $^{1}$H-NMR and $^{13}$C-NMR of 20D** |
| **S48** | **Figures S36 and S37: $^{1}$H-NMR and $^{13}$C-NMR of 21-Ms** |

| | |
|---|---|
| **S49** | **Figures S38 and S39: $^{1}$H-NMR and $^{13}$C-NMR of 21-Ac** |
| **S50** | **Figures S40 and S41: $^{1}$H-NMR and $^{13}$C-NMR of 21-Ts** |
| **S51** | **Figures S42 and S43: $^{1}$H-NMR and $^{13}$C-NMR of 22** |
| **S52** | **Figures S44 and S45: $^{1}$H-NMR and $^{13}$C-NMR of 23** |
| **S53** | **Figures S46 and S47: $^{1}$H-NMR and $^{13}$C-NMR of 24** |
| **S54** | **Figures S48 and S49: $^{1}$H-NMR and $^{13}$C-NMR of 25** |
| **S55** | **Figures S50 and S51: $^{1}$H-NMR and $^{13}$C-NMR of 26** |
| **S56** | **Figure S52: $^{1}$H-NMR of 21' and 26** |
| **S57** | **Figures S53 and S54: $^{1}$H-NMR and $^{13}$C-NMR of 27** |
| **S58** | **Computational Methods and DFT Analysis for 7D** |
| **S59-S60** | **Table S1: DFT Geometry Optimization, Energies and Coordinates for *erythro*-7D** |
| **S61-S62** | **Table S2: DFT Geometry Optimization, Energies and Coordinates for *threo*-7D** |
| **S63** | **Figure S55: H-H Bond Lengths and NOESY Interactions** |
| **S64** | **Chiral HPLC Parameters, Figures S56-S58** |
| **S65** | **Figure S59 and Table S3: SC-XRD of 6D** |
| **S66** | **Figure S60 and Table S4: SC-XRD of *erythro* 7D** |
| **S67** | **Figure S61 and Table S5: SC-XRD of 8** |
| **S68** | **Figure S62 and Table S6: SC-XRD of 9D** |
| **S69** | **Figure S63 and Table S7: SC-XRD of 13D** |
| **S70** | **Figure S64 and Table S8 : SC-XRD of 19D** |
| **S71** | **Figure S65 and Table S9: SC-XRD of 21-Ms** |
| **S72** | **Figure S66 and Table S10: SC-XRD of 21-Ac** |
| **S73** | **Figure S67 and Table S11: SC-XRD of 22** |
| **S74** | **Figure S68 and Table S12: SC-XRD of 26** |
| **S75-S76** | **Figure S69 and Table S13 : SC-XRD of 27; References** |

**General Methods**:

NMR spectra were obtained on an 800 MHz spectrometer. Chemical shifts are referenced to tertramethylsilane (TMS) utilizing residual $^{1}H$ or $^{13}C$ signals of the deuterated solvents as internal standards. Chemical shifts are reported in ppm, and coupling constants (*J*) are reported in hertz (Hz). Infrared Spectra (IR) were recorded on a spectrometer as a glaze on a diamond anvil ATR assembly, with peaks reported in $cm^{-1}$. Electrochemical experiments were performed under a nitrogen atmosphere. Most cyclic voltammetric (CV) data were recorded at ambient temperature at 100 mV/s, unless otherwise noted, with a standard three-electrode cell from +1.25 V to -1.25 V with a platinum working electrode, *N,N*-dimethylacetamide (DMA) or acetonitrile solvent, and tetrabutylammonium hexafluorophosphate (TBAH) electrolyte (~1.0 M). All potentials are reported versus the normal hydrogen electrode (NHE) using cobaltocenium hexafluorophosphate ($E_{1/2}$ = -0.78 V, -1.75 V) or ferrocene ($E_{1/2}$ = 0.55 V) as an internal standard. The peak separation of all reversible couples was less than 100 mV. All synthetic reactions were performed in a glovebox under a dry nitrogen atmosphere unless otherwise noted. All solvents were purged with nitrogen prior to use. Deuterated solvents were used as received from Cambridge Isotopes. NMR assignments of all compounds were determined using 2D NMR methods, including NOESY, COSY, HMBC, and HSQC. When possible, pyrazole (Pz) protons of the (trispyrazolyl) borate (Tp) ligand were uniquely assigned (e.g., "PzB3") using two-dimensional NMR data (see Figure S1). If unambiguous assignments were not possible, Tp protons were labeled as "Pz3/5 or Pz4". All *J* values for Pz protons are 2 (±0.4) Hz. BH peaks (around 4-5 ppm) in the $^{1}H$ NMR spectra are not assigned due to their quadrupole broadening. High-resolution electrospray ionization mass spectrometry (ESI-MS analyses were taken on an Agilent 6545B Q-TOF LC/MS using purine and hexakis(1H, 1H, 3H-tetrafluoropropoxy)phosphazine as internal standards. Samples were dissolved in MeCN and eluted with a MeCN/$H_2O$ solution containing 0.1% formic acid.

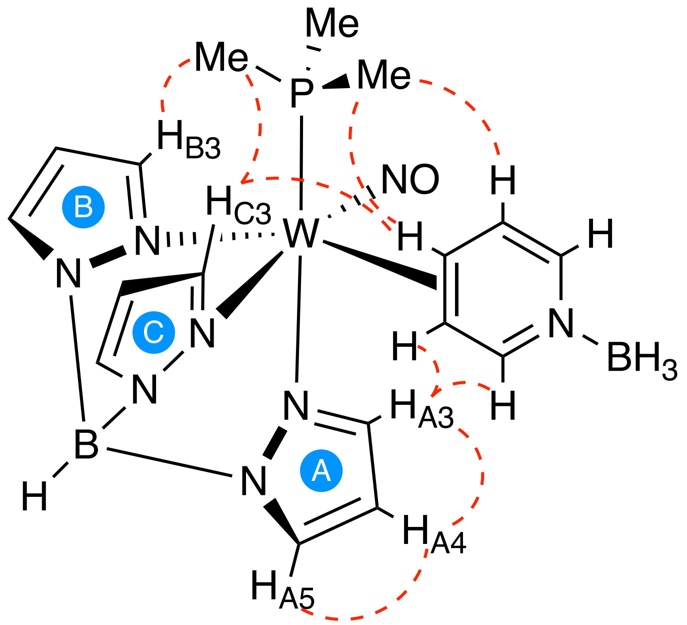

**Synthesis and characterization of WTp(NO)($PMe_3$)($\eta^2$-(*N*-mesyl)pyridinium) (OTf) (5D)**

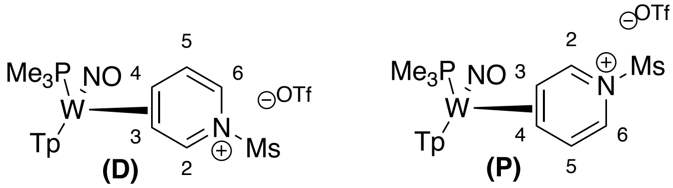


***D:P > 20:1***

Methanesulfonic anhydride (5.87 g, 33.70 mmol), WTp(NO)($PMe_3$)($\eta^2$-pyridinium)(OTf) (9.87 g, 13.48 mmol), and EtCN (40 mL) were charged to a flame-dried 100 mL round-bottom flask with a 1-inch stir bar. Lutidine (3.69 g, 34.43 mmol) was added to initiate the reaction, and the flask was submerged in a pre-heated oil bath set to 55 °C. After stirring the solution for ~2 hours, the solution was diluted with 150 mL of DCM and added to a separatory funnel. This solution was washed 3x with 200 mL of saturated aqueous $NaHCO_3$. The organic layer was isolated and set aside. The combined aqueous layers were combined and back-extracted with 50 mL of DCM to prevent product loss. The organic layers were combined in a single flask and dried with anhydrous $MgSO_4$. This powder was then filtered off into a 60 mL coarse porosity fritted funnel and washed with DCM. The dried organic layers were then reduced *in vacuo* down to dryness. The residue in the flask was redissolved in minimal DCM (approximately 10 mL). The solution was then slowly added to 500mL of stirring diethyl ether. An orange precipitate formed immediately and was allowed to stir for ~10 minutes to ensure total precipitation. This powder was collected on a 60 mL medium porosity frit and washed 2x with 30 mL of ether. This powder was dried in a desiccator under vacuum for ~30 minutes. The dried powder was gently added to a stirring solution of 150 mL of HPLC grade ethyl acetate and triturated overnight. The final orange precipitate was collected on the F frit, washed 2x with 30 mL of ethyl acetate and 2x with 30 mL of diethyl ether, and dried in the desiccator overnight under vacuum (5.5 g, 50% yield).

$^1$H NMR ($CD_2Cl_2$, δ, 25 °C): ***(D)***:9.14 (d, *J* = 5.9 Hz, 1H, H2), 7.97 (d, *J* = 2.1 Hz, 1H, PzB3), 7.96 (d, *J* = 2.3 Hz, 1H, PzC5), 7.93 (d, *J* = 2.4 Hz, 1H, PzB5), 7.90 (d, *J* = 2.1 Hz, 1H, PzA3), 7.76 (d, *J* = 2.4 Hz, 1H, PzA5), 7.65 (d, *J* = 2.1 Hz, 1H, PzC3), 6.64 (ddd, *J* = 1.8, 5.6, 7.8 Hz, 1H, H5), 6.48 (t, *J* = 2.3 Hz, 1H, PzC4), 6.45 (dd, *J* = 1.6, 7.8 Hz, 1H, H6), 6.44 (t, *J* = 2.2 Hz, 1H, PzB4), 6.39 (t, *J* = 2.3 Hz, 1H, PzA4), 4.16 (dt, *J* = 6.2, 12.4 Hz, 1H, H4), 3.61 (s, 3H, Ms), 3.08 (td, *J* = 1.8, 6.2 Hz, 1H, H3), 1.26 (d, $J_{PH}$ = 9.2 Hz, 9H, $PMe_3$). ***(P)***: 9.43 (d, *J* = 5.1 Hz, 1H, H2), (2 other Pz3/5 signals buried) 7.93 (d, *J* = 2.3 Hz, 1H, Pz3/5), 7.91 (d, *J* = 2.4 Hz, 1H, Pz3/5), 7.78 (d, *J* = 2.4 Hz, 1H, Pz3/5), 7.58 (d, *J* = 2.1 Hz, 1H, Pz3/5), 6.81 (dd, *J* = 1.0, 6.7 Hz, 1H, H5), 6.42 (t, *J* = 2.3 Hz, 1H, Pz4), 6.41 (t, *J* = 2.3 Hz, 1H, Pz4), 6.32 (t, *J* = 2.2 Hz 1H, Pz4), 6.31 (dd, *J* = 1.4, 7.3 Hz, 1H, H6), 4.21 (q, *J* = 6.2 Hz, 1H, H4), 3.71 (s, 3H, Ms), 2.86 (t, *J* = 6.7 Hz, 1H, H3), 1.33 (d, $J_{PH}$ = 8.80 Hz, 9H, $PMe_3$).

$^{13}$C NMR ($CD_2Cl_2$, δ, 25 °C): ***(D)***: 167.9 (C2), 146.8 (PzA3), 145.8 (d, $J_{PC}$ = 2.0 Hz, PzB3), 141.9 (PzC3), 138.9 (PzC5), 138.7 (PzB5), 137.8 (PzA5), 124.1 (d, $J_{PC}$ = 3.0 Hz, C5), 121.5 (q, $J_{FC}$ = 320.5 Hz, $TfO^-$), 115.2 (C6), 108.4 (PzC4), 108.3 (PzB4), 108.0 (PzA4), 67.1 (d, $J_{PC}$= 13.9 Hz, C4), 66.1 (C3), 44.2 (Ms), 13.2 (d, $J_{PC}$ = 31.3 Hz, 3C, $PMe_3$). ***(P)***: 163.0 (C2), 144.7 (Pz3/5), 142.4 (Pz3/5), 141.1 (Pz3/5), 138.6 (Pz3/5), 138.1 (Pz3/5), 137.1 (Pz3/5), 127.3 (C5), 112.6 (C6), 108.1 (Pz4), 108.0 (Pz4), 106.9 (Pz4), 68.1 (C4), 65.1 (C3), 43.4 (Ms), 13.8 (d, $J_{PC}$ = 30.3 Hz, 3C, $PMe_3$).

IR $\nu$(NO) = 1617 cm$^{-1}$, $\nu$(BH) = 2519 cm$^{-1}$.
CV (DMA; 100 mV/s) $E_{p,a}$ = +1.18 V (NHE), $E_{p,c}$ = -0.96 V (NHE).
HRMS (ESI) m/z: $[M]^+$ Calcd for $C_{18}H_{27}BN_8O_3PSW^+$ 661.1261; Found 661.1261

**Synthesis and characterization of WTp(NO)($PMe_3$)($\eta^2$-(*N*-mesyl)-2-(methylacetate)-1,2-dihydropyridine) (6D)**

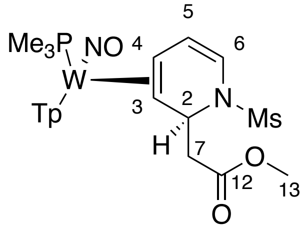


Methyl bromoacetate (107 mg, 0.70 mmol), (**5D**) (204 mg, 0.25 mmol), and THF (3.0 mL) were charged to a flame-dried 50 mL round-bottom flask with a 1-inch stir bar. Zinc powder (67 mg, 1.02 mmol) was added to initiate the reaction, and the heterogenous solution was stirred for 1 hour. The zinc was removed by filtering the solution through a celite plug set up in a 60 mL coarse porosity frit with 1 inch of celite, which was then washed with residual DCM to prevent loss of product. The solution was diluted with 150 mL of DCM and added to a separatory funnel. This solution was washed 3x with 200 mL of saturated aqueous $NaHCO_3$. The organic layer was isolated and set aside. The combined aqueous layers were combined and back-extracted with 50 mL of DCM to prevent product loss. The organic layers were combined in a single flask and dried with anhydrous $MgSO_4$. This powder was then filtered off into a 60 mL coarse porosity fritted funnel and washed with DCM. The dried organic layers were then reduced *in vacuo* to dryness. The residue in the flask was redissolved in minimal DCM (approximately 10 mL). The solution was then slowly added to 200mL of stirring pentane. A tan precipitate formed immediately and was allowed to stir for ~10 minutes to ensure total precipitation. This powder was collected on a 60 mL medium porosity frit and washed 2x with 30 mL of pentane. This powder was dried in a desiccator under vacuum for ~30 minutes (178 mg, 96% yield).

$^1$H NMR ($CD_2Cl_2$, δ, 25 °C): 8.12 (d, $J$ = 1.9 Hz, 1H, PzA3), 8.08 (d, $J$ = 1.9 Hz, 1H, PzB3), 7.78 (d, $J$ = 2.3 Hz, 1H, PzB5), 7.75 (d, $J$ = 2.3 Hz, 1H, PzC5), 7.69 (d, $J$ = 2.3 Hz, 1H, PzA5), 7.28 (d, $J$ = 2.1 Hz, 1H, PzC3), 6.36 (t, $J$ = 2.2 Hz, 1H, PzB4), 6.32 (t, $J$ = 2.3 Hz, 1H, PzA4), 6.23 (t, $J$ = 2.3 Hz, 1H, PzC4), 5.86 (d, $J$ = 7.8 Hz, 1H, H6), 5.66 (ddd, $J$ = 0.7, 5.1, 7.8 Hz, 1H, H5), 5.06 (m, 1H, H2), 3.47 (s, 3H, H9), 3.19 (s, 3H, Ms), 3.09 (dd, $J$ = 3.8, 14.7 Hz, 1H, H7), 3.00 (dd, $J$ = 9.0, 14.7 Hz, 1H, H7’), 2.79 (ddd, $J$ = 5.1, 10.6, 13.1 Hz, 1H, H4), 1.55 (d, $J$ = 10.6 Hz, 1H, H3), 1.17 (d, $J_{PH}$ = 8.5 Hz, 9H, $PMe_3$).

$^{13}$C NMR ($CD_2Cl_2$, δ, 25 °C): 172.5 (C8), 143.5 (PzA3), 143.3 (d, $J_{PC}$ = 1.7 Hz, PzB3), 140.7 (PzC3), 137.3 (PzC5), 136.6 (PzB5), 136.4 (PzA5), 115.3 (C6), 112.6 (d, $J_{PC}$ = 3.0 Hz, C5), 107.1 (PzB4), 106.6 (PzC4), 106.4 (PzA4), 62.2 (d, $J_{PC}$ = 1.4 Hz, C3), 53.2 (C2), 51.7 (C10), 44.9 (C7), 44.5 (d, $J_{PC}$ = 10.5 Hz, C4), 42.4 (Ms), 13.3 (d, $J_{PC}$ = 28.6 Hz, 3C, $PMe_3$).

IR $\nu$(NO) = 1537 cm$^{-1}$, $\nu$(BH) = 2483 cm$^{-1}$, $\nu$(C=O) = 1725 cm$^{-1}$
CV (DMA; 50 mV/s) $E_{p,a}$ = +0.53 V (NHE)
SC-XRD data on S65.

**Synthesis and characterization of WTp(NO)(PMe$_3$)($\eta^2$-(*N*-mesyl)-2-(methyl-*α*-phenylacetate)-1,2-dihydropyridine) (7D)**

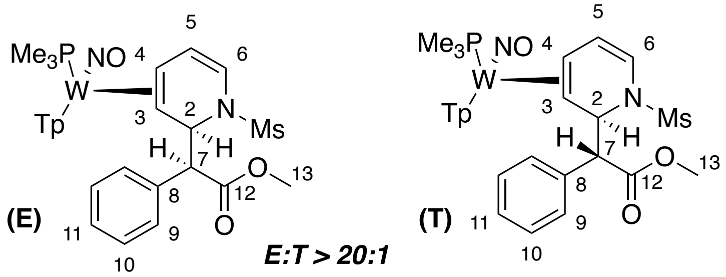


Methyl *α*-phenylbromoacetate (1.69 g, 7.38 mmol), (**5D**) (1.75 g, 2.16 mmol), and THF (10 mL) were charged to a flame-dried 50 mL round-bottom flask with a 1-inch stir bar. Zinc powder (490 mg, 7.49 mmol) was added to initiate the reaction, and the heterogenous solution was stirred for 1 hour. The zinc was removed by filtering the solution through a celite plug set up in a 60 mL coarse porosity frit with 1 inch of celite, which was then washed with residual DCM to prevent loss of product. The solution was diluted with 150 mL of DCM and added to a separatory funnel. This solution was washed 3x with 200 mL of saturated aqueous $NaHCO_3$. The organic layer was isolated and set aside. The combined aqueous layers were combined and back-extracted with 50 mL of DCM to prevent product loss. The organic layers were combined in a single flask and dried with anhydrous $MgSO_4$. This powder was then filtered off into a 60 mL coarse porosity fritted funnel and washed with DCM. The dried organic layers were then reduced *in vacuo* to dryness. The residue in the flask was redissolved in minimal DCM (approximately 10 mL). The solution was then slowly added to 200mL of stirring pentane. A tan precipitate formed immediately and was allowed to stir for ~10 minutes to ensure total precipitation. This powder was collected on a 60 mL medium porosity frit and washed 2x with 30 mL of pentane. This powder was dried in a desiccator under vacuum for ~30 minutes. The dried powder was gently added to a 4-dram vial containing a stir pea, filled with 10 mL of stirring MeOH, and triturated overnight. This trituration is performed to remove residual paramagnetic tungsten impurities; however, eluting the product off a basic alumina plug with ethyl acetate and subsequently precipitating the reduced solution in pentane will suffice to replace this step. The tan precipitate was collected on a 15 mL fine porosity frit and washed 2x with 10 mL of MeOH. After drying in a desiccator for 2 hours, the highly pure tan precipitate was added to a 4-dram vial and stir pea filled with 10 mL of DME and triturated overnight. This step is where enrichment occurs, but is only successful when the material has no tungsten impurities by $^1$H NMR or CV. Both diastereomers are partially soluble in DME, so having a batch that is slightly enriched (>3:1 c.d.r.) is crucial. A 1:1 c.d.r. batch will not enrich via this approach. After stirring overnight, the highly enriched tan powder was collected on a 15 mL fine porosity frit and washed 1x with 10 mL of DME and 2x with 10 mL of diethyl ether. This powder was then dried in the desiccator (32% yield).

$^1$H NMR ($CD_2Cl_2$, δ, 25 °C): ***(E)***: 8.15 (d, *J* = 1.8 Hz, 1H, PzA3), 8.10 (d, *J* =1.9 Hz, 1H, PzB3), 7.78 (d, *J* = 2.4 Hz, 1H, PzB5), 7.75 (d, *J* = 2.3 Hz, 1H, PzC5), 7.67 (d, *J* = 2.4 Hz, 1H, PzA5), 7.41 (m, 2H, H9), 7.25 (m, 1H, PzC3), 7.24 (m, 2H, H10), 7.20 (m, 1H, H11), 6.35 (t, *J* = 2.2 Hz, 1H, PzB4), 6.29 (t, *J* = 2.2 Hz, 1H, PzA4), 6.23 (t, *J* = 2.2 Hz, 1H, PzC4), 5.78 (ddd, *J* = 0.7, 5.0, 7.7 Hz, 1H, H5), 5.72 (d, *J* = 7.7 Hz, 1H, H6), 5.22 (dd, *J* = 1.1, 8.3 Hz, 1H, H2), 4.34 (d, *J* = 8.3 Hz, 1H, H7), 3.42 (s, 3H, H13), 2.90 (ddd, *J* = 4.9, 10.7, 14.5 Hz, 1H, H4), 2.60 (s, 3H, Ms),1.64 (dd, *J* = 1.1, 10.7 Hz, 1H, H3), 1.20 (d, $J_{PH}$ = 8.5 Hz, 9H, $PMe_3$). ***(T)***: 8.14 (d, *J* = 1.8 Hz, 1H, Pz3/5), 8.05 (d, *J* = 1.9 Hz, 1H, Pz3/5), 7.77 (d, *J* = 2.4 Hz, 1H, Pz3/5), 7.73 (d, *J* = 2.2 Hz, 1H, Pz3/5), 7.68 (d, *J* = 2.3 Hz, 1H, Pz3/5), (H9, 10, and 11 are buried), 6.34 (t, *J* = 2.2 Hz, 1H, Pz4),6.33 (t, *J* = 2.2 Hz, 1H, Pz4), 6.21 (t, *J* = 2.2 Hz, 1H, Pz4), 5.61 (d, *J* = 7.8 Hz, 1H, H6), 5.50 (dd, *J* = 0.9, 5.4 Hz, 1H, H2), 5.17 (dd, *J* = 4.9, 7.8 Hz, 1H, H5), 4.54 (d, *J* = 5.4 Hz, 1H, H7), 3.44 (s, 3H,

H13), 3.23 (s, 3H, Ms), 2.54 (ddd, $J$ = 4.9, 10.7, 13.6 Hz, 1H, H4), 1.42 (dd, $J$ = 0.9, 10.7 Hz, 1H, H3), 1.08 (d, $J_{PH}$ = 8.5 Hz, 9H, $PMe_3$).

$^{13}$C NMR ($CD_2Cl_2$, δ, 25 °C): ***(E)***: 173.70 (C12), 144.17 (PzA3), 143.10 (d, $J_{PC}$ = 1.6 Hz, 1C, PzB3), 140.16 (PzC3), 137.83 (C8), 137.22 (PzC5), 136.61 (PzB5), 136.11 (PzA5),130.29 (2C, C9), 128.51 (2C, C10), 127.90 (C11), 116.02 (C6), 114.92 (d, $J_{PC}$ = 2.6 Hz, 1C, C5), 107.01 (PzB4), 106.61 (PzC4), 106.40 (PzA4), 64.11 (d, $J_{PC}$ = 1.6 Hz, 1C, C3), 60.19 (C7), 59.36 (C2), 52.02 (C13), 44.57 (d, $J_{PC}$ = 10.4 Hz, 1C, C4), 42.15 (Ms), 13.25 (d, $J_{PC}$ = 28.0 Hz, 3C, $PMe_3$). ***(T)***: 173.11 (C12), 143.65 (Pz3/5), 143.18 (d, $J_{PC}$ = 2.2 Hz, 1C, Pz3/5), 140.36 (Pz3/5), 137.26 (Pz3/5), 136.81 (Pz3/5), 136.41 (Pz3/5), (C8 obscured), 131.52 (2C, C9/C10), 127.73 (2C, C9/C10), 127.22 (C11), 115.94 (C6), 112.22 ((d, $J_{PC}$ = 3.0 Hz, 1C, C5), 107.07 (Pz4), 106.61 (Pz4), 106.26 (Pz4), 60.98 (C7), 58.25 (C2), 57.85 (d, $J_{PC}$ = 1.4 Hz, 1C, C3), 52.00 (C13), 46.16 (d, $J_{PC}$ = 10.6 Hz, 1C, C4), 42.37 (Ms), 13.10 (d, $J_{PC}$ = 28.0 Hz, 3C, $PMe_3$).

IR: $\nu$(NO) = 1557 cm$^{-1}$, $\nu$(BH) = 2489 cm$^{-1}$, $\nu$(CO) = 1732 cm$^{-1}$.

CV (DMA; 100 mV/s): $E_{p,a}$ = +0.52 V (NHE).

SC-XRD data on S66.

**Synthesis and characterization of WTp(NO)($PMe_3$)($\eta^2$-(*N*-mesyl)-2-(methyl-$\alpha$-phenylacetate)-1,2,3,6-tetrahydropyridinium (OTf) (8)**

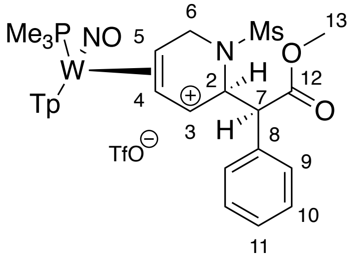

***Erythro*-7D** (97 mg, 0.12 mmol) was added to a 4-dram vial containing a stir pea and diluted with propionitrile (3 mL). To a separate 4-dram vial, propionitrile (1 mL) and TfOH (24 mg, 0.16 mmol) were added. The acid/propionitrile solution was slowly added to the ***erythro*-7D**/propionitrile solution and stirred for 5 minutes. The reaction was then slowly added to 100 mL of stirring diethyl ether, which immediately formed a tan precipitate. This powder was triturated in the ether solution for 10 minutes and then collected on a 30 mL medium porosity frit. The collected powder was then washed 2x with 20 mL of diethyl ether and dried in a desiccator (104 mg, 90% yield).

$^{1}$H NMR ($CD_2Cl_2$, δ, 25 °C): 8.31 (d, $J$ = 2.2 Hz, 1H, PzB3), 8.14 (d, $J$ = 2.3 Hz, 1H, PzC3), 7.90 (d, $J$ = 2.3 Hz, 1H, PzC5), 7.81 (d, $J$ = 2.3 Hz, 1H, PzB5), 7.60 (d, $J$ = 2.4 Hz, 1H, PzA5), 7.54 (d, $J$ = 7.4 Hz, 2H, H9), 7.46 (t, $J$ = 7.4 Hz, 2H, H10), 7.42 (t, $J$ = 7.4 Hz, 1H, H11), 6.86 (d, $J$ = 2.2 Hz, 1H, PzA4), 6.58 (t, $J$ = 2.3 Hz, 1H, PzC4), 6.47 (t, $J$ = 2.4 Hz, 1H, PzB4), 5.98 (t, $J$ = 2.4 Hz, 1H, PzA4), 5.89 (d, $J$ = 7.9 Hz, 1H, H3), 5.46-5.48 (m, 1H, H2), 5.23 (t, 7.8 Hz, 1H, H4), 4.81 (dd, $J$ = 2.3, 12.7 Hz, 1H, H6), 4.78 (d, $J$ = 5.3 Hz, 1H, H7), 4.53-4.58 (m, 1H, H5), 4.12 (d, $J$ = 12.7 Hz, 1H, H6'), 3.84 (s, 3H, H13), 2.95 (s, 3H, Ms), 1.27 (d, $J_{PH}$ = 9.7 Hz, 9H, $PMe_3$).

$^{13}$C NMR ($CD_2Cl_2$, δ, 25 °C): 171.3 (C12), 146.4 (PzA3), 144.4 (d, $J_{PC}$ = 2.2 Hz, PzB3), 142.7 (PzC3), 138.9 (PzC5), 138.7 (PzA5/B5), 138.6 (PzA5/B5), 134.6 (C8), 129.7 (2C, C10), 129.0 (2C, C9), 128.8 (C11), 121.4 (q, $J_{FC}$ = 320.4 Hz, $TfO^-$), 120.0 (C3), 109.1 (PzB4/C4), 108.9 (PzB4/C4), 107.3 (PzA4), 98.3 (d, $J_{PC}$ = 3.0 Hz, C4), 65.3 (d, $J_{PC}$ = 14.5 Hz, C5), 58.7 (C2), 55.9 (C7), 53.0 (C13), 42.9 (d, $J_{PC}$ = 2.5 Hz, C6), 41.1 (Ms), 13.5 (d, $J_{PC}$ = 33.0 Hz, 3C, $PMe_3$).

IR: $\nu$(NO) = 1661 cm$^{-1}$, $\nu$(BH) = 2511 cm$^{-1}$, $\nu$(CO) = 1733 cm$^{-1}$.

CV (MeCN; 100 mV/s): $E_{p,c}$ = -0.74 V (NHE).

HRMS (ESI) m/z: $[M]^+$ Calcd for $C_{27}H_{37}BN_8O_5PSW^+$ 811.1942; Found 811.1945

SC-XRD data on S67.

**Synthesis and characterization of WTp(NO)(PMe$_3$)($\eta^2$-(*N*-mesyl)-2-(methyl-$\alpha$-phenylacetate)-1,2,5,6-tetrahydropyridine) (9D)**

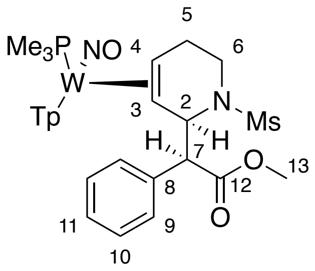


Cyanoborohydride (136 mg, 2.16 mmol) and methanol (2.0 mL) were added to a screw-cap test tube and placed in a cold bath at -50 °C. To another tube, ***erythro*-7D** (185 mg, 0.228 mmol) and propionitrile (3.0 mL) were added. Triflic acid (41 mg, 0.273 mmol) was then added to the ***erythro*-7D** solution, which was swirled until dissolution and then also added to the cold bath. Both tubes were chilled for approximately 15 minutes before the cyanoborohydride/methanol solution was added to the solution of **8**, which sat in the cold bath for 16 hours. This solution was diluted with 50 mL of DCM and was washed 3x with 50 mL of saturated aqueous $NaHCO_3$. The organic layer was isolated and set aside. The combined aqueous layers were combined and back-extracted with 50 mL of DCM to prevent product loss. The organic layers were combined in a single flask and dried with anhydrous $MgSO_4$. This powder was then filtered off into a 60 mL coarse porosity fritted funnel and washed with DCM. The dried organic layers were then reduced *in vacuo* down to dryness. The residue in the flask was redissolved in minimal DCM (approximately 10 mL). The solution was then slowly added to 100 mL of stirring pentane. A tan precipitate formed immediately and was allowed to stir for ~10 minutes to ensure total precipitation. This powder was collected on a 15 mL fine porosity frit and washed 2x with 10 mL of pentane. This powder was dried in a desiccator under vacuum overnight (172 mg, 93% yield).

$^1$H NMR (($(CD_3)_2CO$, δ, 25 °C): 8.27 (d, $J$ = 1.8 Hz, 1H, PzA3), 8.10 (d, $J$ = 1.8 Hz, 1H, PzB3), 7.92 (d, $J$ = 2.3 Hz, 1H, PzC5), 7.91 (d, $J$ = 2.4 Hz, 1H, PzB5), 7.78 (d, $J$ = 2.3 Hz, 1H, PzA5), 7.43 (d, $J$ = 2.0 Hz, 1H, PzC3), 7.35-7.38 (m, 2H, H9), 7.15-7.17 (m, 3H, H10/11), 6.38 (t, $J$ = 2.2 Hz, 1H, PzB4), 6.32 (t, $J$ = 2.2 Hz, 1H, PzC4), 6.26 (t, $J$ = 2.2 Hz, 1H, PzA4), 5.57 (dd, $J$ = 1.5, 6.8 Hz, 1H, H2), 4.13 (d, $J$ = 6.8 Hz, 1H, H7), 3.41 (dt, $J$ = 4.3, 13.0 Hz, H6'), 3.22 (s, 3H, H13), 2.98 (ddd, $J$ = 3.8, 10.9, 14.6 Hz, 1H, H6), 2.86-2.92 (m, 1H, H4), 2.64-2.70 (m, 1H, H5'), 2.58-2.62 (m, 1H, H5), 2.49 (s, 3H, Ms), 1.26 (d, $J_{PH}$ = 8.4 Hz, 9H, $PMe_3$), 0.94 (d, $J$ = 11.7 Hz, 1H, H3).

$^{13}$C NMR: ($(CD_3)_2CO$, δ, 25 °C): 172.8 (C12), 144.0 (PzA3), 143.9 (d, $J_{PC}$ = 1.5 Hz, PzB3), 141.2 (PzC3), 138.1 (C8), 137.4 (PzB5/C5), 137.0 (PzB5/C5), 136.6 (PzA5), 130.8 (2C, C9), 128.6 (2C, C10), 127.9 (C11), 107.0 (PzB4), 106.9 (PzC4), 106.6 (PzA4), 63.3 (C7), 60.7 (C2), 53.8 (t, $J_{WC}$ = 32.3 Hz, C3), 51.4 (C13), 47.3 (d, $J_{PC}$ = 12.5 Hz, C4), 42.0 (C6), 40.5 (Ms), 28.8 (d, $J_{PC}$ = 2.2 Hz, C5), 13.5 (d, $J_{PC}$ = 27.9 Hz, 3C, $PMe_3$).

IR: $\nu$(NO) = 1538 cm$^{-1}$, $\nu$(BH) = 2486 cm$^{-1}$, $\nu$(CO) = 1729 cm$^{-1}$
CV (DMA; 100 mV/s): $E_{p,a}$ = +0.44 V (NHS)
HRMS (ESI) m/z: [M]$^+$ Calcd for $C_{27}H_{37}BN_8O_5PSW^+$ 811.1942; Found 811.1945
SC-XRD data on S68.

**Synthesis and characterization of WTp(NO)(PMe$_3$)($\eta^2$-(*N*-mesyl)-5-dimethylpropanedioate-2-(methyl-$\alpha$-phenylacetate)-1,2,5,6-tetrahydropyridine (10D)**

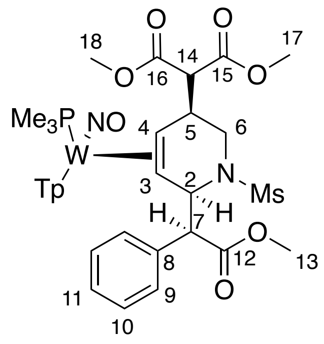


***Erythro*-7D** (200 mg, 0.247 mmol) was dissolved in 1 mL acetonitrile in a test tube with a stir pea. HOTf (38 mg, 0.253 mmol) was added to the test tube, and the mixture was stirred at room temperature for 5 minutes. The dark brown solution was cooled at -10 °C for 15 minutes. Lithium dimethylmalonate (227 mg, 1.64 mmol) was dissolved in 1 mL acetonitrile in a test tube with a stir pea. The solution was then cooled to -10 °C for 15 minutes. The allyl solution was added to the lithium dimethylmalonate solution and reacted for 30 minutes at 0 °C. The solution was stirred at room temperature for an additional 30 minutes. The solution was diluted with DCM (10mL), washed with $H_2O$ (3 x 5 mL), and the aqueous solution was back extracted with DCM (3 x 2mL). The solution was dried with $MgSO_4$, removed on a 15 mL medium porosity frit, washed with DCM (3 x 2 mL), and the resulting filtrate was evaporated to dryness. The product was dissolved in minimal DCM and added to a stirring solution of hexanes (75 mL), yielding a precipitate. This precipitate was isolated on a 15 mL fine porosity frit, washed with hexanes (10 mL), and desiccated to yield **10D** (160 mg, 69% yield).

$^1$H NMR ($CD_2Cl_2$, δ, 25 °C): 8.42 (d, $J$ = 1.0 Hz, 1H, PzA3), 8.08 (d, $J$ = 1.8 Hz, 1H, PzB3), 7.77 (d, $J$ = 2.2 Hz, 1H, PzC5), 7.76 (d, $J$ = 2.5 Hz, 1H, PzB5), 7.70 (d, $J$ = 2.3 Hz, 1H, PzA5), 7.42 (d, J = 7.2 Hz, 2H, H9), 7.30 (t, $J$ = 7.3 Hz, 2H, H10), 7.25 (t, $J$ = 7.3, 1H, H11), 7.07 (d, $J$ = 2.0 Hz, 1H, PzC3), 6.34 (t, $J$ = 2.5 Hz, 1H, PzA4), 6.31 (t, $J$ = 2.2 Hz, 1H, PzB4), 6.26 (t, $J$ = 2.2 Hz, 1H, PzC4), 5.54 (d, $J$ = 9.4 Hz, 1H, H2), 4.13 (d, $J$ = 9.6 Hz, 1H, H7), 3.95 (bs, 4H, H14/17/18) 3.77 (s, 3H, H17/18), 3.49 (m, 2H, H6/H5), 3.38 (m, 1H, H6), 2.97 (s, 3H, H13), 2.57 (ddd, $J$ = 3.4, 13.7, 15.1 Hz, 1H, H4), 1.88 (s, 3H, Ms), 1.23 (d, $J_{PH}$ = 8.0 Hz, 9H, PMe$_3$), 0.72 (d, $J$ = 6.6 Hz, 1H, H3).

$^{13}$C NMR ($CD_2Cl_2$, δ, 25 °C): 172.3 (C12), 169.5/169.3 (2C, C15/16), 144.6 (PzA3), 143.8 (PzB3), 140.4 (PzC3), 137.6 (C8), 137.1 (PzC5), 136.9 (PzB5), 136.5 (PzA5), 129.9 (2C, C9), 129.0 (2C, C10), 128.4 (C11), 106.4 (PzA4), 106.7 (PzB4), 106.6 (PzC4), 61.8 (C7), 58.7 (C2), 56.8 (C3), 56.3 (C14), 53.0/53.1 (2C, C17/18), 51.5 (C13), 46.6 (d, $J_{PC}$ = 12.0 Hz, C4), 40.4 (C6), 39.5 (C5), 37.1 (Ms), 14.5 (d, $J_{PC}$ = 27.6 Hz, 3C, PMe$_3$).

IR: $\nu$(NO) = 1543 cm$^{-1}$, $\nu$(CO) = 1728 cm$^{-1}$
CV (DMA; 100 mV/s): $E_{p,a}$ = +0.61 V (NHE)
HRMS (APCI) m/z: [M]$^+$ Calcd for $C_{32}H_{46}BN_8O_9PSW^+$ 943.2365; Found 943.2368

**Synthesis and characterization of WTp(NO)($PMe_3$)($\eta^2$-(*N*-mesyl)-5-cyano-2-(methyl-$\alpha$-phenylacetate)-1,2,5,6-tetrahydropyridine (11D)**

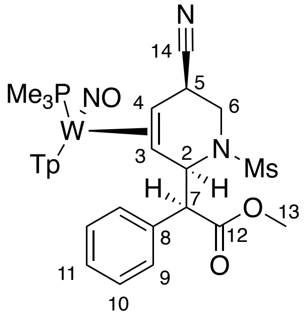


***Erythro*-7D** (469 mg, 0.578 mmol) was dissolved in 3 mL propionitrile in a test tube with a stir pea. HOTf (375 mg, 2.50 mmol) was added to the test tube, and the mixture was allowed to stir at room temperature for 5 minutes. The dark brown solution was cooled at -40 °C for 15 minutes. Sodium cyanide (235 mg, 4.79 mmol) was dissolved in 3 mL methanol in a test tube with a stir pea. The solution was then cooled to -40 °C for 15 minutes. The allyl solution was added to the sodium cyanide solution and reacted for 12 hours at -40 °C. The solution was warmed to room temperature. The solution was diluted with DCM (10 mL), washed with $Na_2CO_3$ (3 x 5 mL), and the aqueous solution was back extracted with DCM (3 x 2 mL). The solution was dried with $MgSO_4$, removed on a 15 mL medium porosity frit, washed with DCM (3 x 2 mL), and the resulting filtrate was evaporated to dryness. The product was dissolved in minimal DCM and added to a stirring solution of hexanes (75 mL), yielding a precipitate. This precipitate was isolated on a 15 mL fine porosity frit, washed with hexanes (10 mL), and desiccated to yield **11D** (421 mg, 87% yield).

$^1$H NMR ($CD_2Cl_2$, δ, 25 °C): 8.44 (d, $J$ = 1.3 Hz, 1H, PzB3), 8.05 (d, $J$ = 1.7 Hz, 1H, PzA5), 7.77 (d, $J$ = 2.2 Hz, 1H, PzA3), 7.76 (d, $J$ = 2.2 Hz, 1H, PzC5), 7.72 (d, $J$ = 2.6 Hz, 1H, PzB5), 7.41(d, $J$ = 7.3 Hz, 2H, H9), 7.32 (t, $J$ = 7.3 Hz, 2H, H10), 7.27 (t, $J$ = 7.7 Hz, 1H, H11), 7.13 (d, $J$ = 1.7 Hz, 1H, PzC3) 6.39 (t, $J$ = 2.2 Hz, 1H, PzB4), 6.34 (t, $J$ = 2.2 Hz, 1H, PzA4), 6.27 (t, $J$ = 2.2 Hz, 1H, PzC4), 5.53 (dd, $J$ = 1.4, 10.4 Hz, 1H, H2), 4.03 (d, $J$ = 10.3 Hz, 1H, H7), 3.82 (ddd, $J$ = 5.1, 5.8, 11.3 Hz, 1H, H5), 3.67 (dd, $J$ = 4.8, 13.2 Hz, 1H, H6), 3.18 (dd, $J$ = 11.3, 13.2 Hz, 1H, H6), 2.92 (ddd, $J$ = 6.0, 11.4, 14.1 Hz, 1H, H4), 2.83 (s, 3H, H13), 1.73 (s, 3H, Ms), 1.30 (d, $J_{PH}$ = 8.0 Hz, 9H, $PMe_3$), 0.46 (d, $J$ = 11.9 Hz, 1H, H3).

$^{13}$C NMR ($CD_2Cl_2$, δ, 25 °C): 171.9 (C12), 144.7 (PzB3), 143.6 (PzA5), 140.0 (PzC3), 137.8 (C13), 136.9 (PzA3), 136.8 (PzC5), 136.4 (PzB5), 129.6 (2C, C9), 129.2 (2C, C10), 128.5 (C11), 123.6 (C14), 106.8 (PzA4), 106.6 (PzB4), 106.6 (PzC4), 61.9 (C7), 58.4 (C2), 52.9 (C3), 51.4 (13), 43.4 (d, $J_{PC}$ = 13.6 Hz, C4), 42.7 (C6), 40.7 (Ms), 29.6 (C5), 14.2 (d, $J_{PC}$ = 29.0 Hz, 3C, $PMe_3$).

IR: $\nu$(NO) = 1556 cm$^{-1}$, $\nu$(CO) = 1732 cm$^{-1}$, $\nu$(CN) = 2225 cm$^{-1}$
CV (DMA; 100 mV/s): $E_{p,a}$ = +0.68 V (NHE)
HRMS (ESI) m/z: [M]$^+$ Calcd for $C_{30}H_{38}BN_9O_5PSW^+$ 838.2051; Found 838.2055

**Synthesis and characterization of WTp(NO)(PMe$_3$)($\eta^2$-(*N*-mesyl)-5-dicyanomethyl-2-(methyl-$\alpha$-phenylacetate)-1,2,5,6-tetrahydropyridine (12D)**

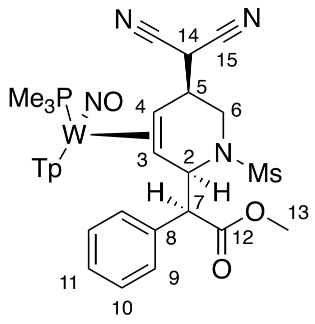


***Erythro*-7D** (200 mg, 0.247 mmol) was dissolved in ~0.5 mL propionitrile in a test tube with a stir pea. HOTf (44 mg, 0.293 mmol) was added to the test tube, and the mixture was allowed to stir at room temperature for 5 minutes. The dark brown solution was cooled to 0 °C for 15 minutes. Malononitrile (162 mg, 2.45 mmol) was dissolved in ~0.5mL THF in a test tube with a stir pea. tBuOK (20% wt., 692 mg, 1.23 mmol) was added. The solution was then cooled to 0 °C for 15 minutes. The allyl solution was added to the deprotonated malononitrile solution and reacted for 4 hours at 0 °C. The solution was warmed to room temperature, diluted with DCM (10 mL), washed with $Na_2CO_3$ (3 x 5 mL), and the aqueous solution was back extracted with DCM (3 x 2 mL). The solution was dried with $Na_2SO_4$, removed on a 15 mL medium porosity frit, washed with DCM (3 x 2 mL), and the resulting filtrate was evaporated to dryness. The product was dissolved in minimal DCM and added to a stirring solution of cold pentanes (75 mL), yielding a precipitate. This precipitate was isolated on a 15 mL fine porosity frit, washed with pentanes (5mL), and desiccated to yield **12D** (177 mg, 82% yield).

$^1$H NMR ($CD_2Cl_2$, δ, 25 °C): 8.28 (bs, 1H, PzA3), 8.17 (d, $J$ = 1.7 Hz, 1H, PzB3), 8.01 (d, $J$ = 2.2 Hz, 1H, PzC5), 7.97 (d, $J$ = 2.4 Hz, 1H, PzB5), 7.82 (d, $J$ = 2.0 Hz, 1H, PzA5), 7.49 (d, $J$ = 2.0, 1H, PzC3), 7.35 (d, $J$ = 7.0 Hz, 1H, H9), 7.26 (t, $J$ = 7.3 Hz, 1H, H11), 7.23 (t, $J$ = 6.2 Hz, 2H, H10), 6.45 (t, $J$ = 2.0 Hz, 1H, PzC4), 6.43 (t, $J$ = 2.4 Hz, 1H, PzB4), 6.24 (t, $J$ = 2.1 Hz, 1H, PzA4), 5.72 (bs, 1H, H2), 4.39 (bs, 1H, H7), 3.74 (dd, $J$ = 3.2, 13.1 Hz, 1H, H6), 3.58 (dd, $J$ = 4.4, 13.1 Hz, 1H, H6), 3.54 (s, 3H, H13), 3.44 (bs, 1H, H5), 3.32 (bs, 1H, H14), 2.91 (s, 3H, Ms), 2.63 (t, $J$ = 11.3 Hz, 1H, H4), 1.38 (dd, $J$ = 11.6 Hz, 1H, H3), 1.25 (d, $J_{PH}$ = 8.5 Hz, 9H, PMe$_3$).

$^{13}$C NMR ($CD_2Cl_2$, δ, 25 °C): 173.1 (C12), 144.3 (PzB3), 143.5 (PzA3), 141.6 (PzC3), 138.3 (PzC5), 137.6 (PzA5), 137.5 (PzB5), 137.0 (C8), 132.0 (2C, C9), 129.1 (2C, C10), 128.1 (C11), 114.2/113.1 (2C, C15), 107.6 (2C, PzC4/PzB4), 107.1 (PzA4), 63.3 (C7), 61.3 (C2), 52.5 (C3), 52.0 (C13), 50.0 (d, $J_{PC}$ = 12.7Hz, C4), 44.2 (C6), 41.3 (C5), 36.0 (Ms), 23.4 (C14), 13.3 (d, $J_{PC}$ = 28.4 Hz, 3C, PMe$_3$).

IR: $\nu$(NO) = 1538 cm$^{-1}$, $\nu$(CO) = 1725 cm$^{-1}$, $\nu$(CN) = 2221 cm$^{-1}$
CV (MeCN; 100 mV/s): $E_{p,a}$ = +0.83 V (NHE)
HRMS (ESI) m/z: [M]$^+$ Calcd for $C_{30}H_{39}BN_{10}O_5PSW^+$ 877.2160; Found 877.2150.

**Synthesis and characterization of WTp(NO)(PMe$_3$)($\eta^2$-(*N*-mesyl)-5-methyl-2-(methyl-$\alpha$-phenylacetate)-1,2,5,6-tetrahydropyridine (13D)**

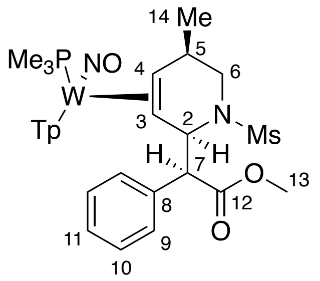


**8** (100 mg, 0.104 mmol) was dissolved in ~0.5 mL propionitrile in a test tube with a stir pea. The solution was cooled to -60 °C for 2 hours. MeMgBr (3M, 0.173 mL, 5.20 mmol) was added to 0.5 mL THF and chilled to -60 °C for 2 hours. The allyl solution was added to the Grignard solution and allowed to react for 60 hours. The solution was warmed to room temperature, diluted with DCM (10 mL), washed with $Na_2CO_3$ (3 x 5 mL), and the aqueous solution was back extracted with DCM (3 x 2 mL). The solution was dried with $Na_2SO_4$, removed on a 15 mL medium porosity frit, washed with DCM (3 x 2 mL), and the resulting filtrate was evaporated to dryness. The product was dissolved in minimal DCM and added to a stirring solution of cold hexanes (75 mL), yielding a precipitate. This precipitate was isolated on a 15 mL fine porosity frit, washed with hexanes (5 mL), and desiccated to yield **13D** (32 mg, 37% yield).

$^1$H NMR ($CD_3CN$, δ, 25 °C): 8.4 (bs, 1H, PzA3), 8.0 (d, *J* = 1.5 Hz, 1H, PzB3), 7.8 (d, *J* = 3.0 Hz, 1H, PzC5), 7.83 (d, *J* = 2.4 Hz, 1H, PzB5), 7.79 (d, *J* = 2.5 Hz, 1H, PzA5), 7.37 (d, *J* = 7.3 Hz, 2H, H9), 7.27 (t, *J* = 7.0 Hz, 2H, H10), 7.23 (t, *J* = 7.0 Hz, 1H, H11), 7.20 (d, *J* = 1.5 Hz, 1H, PzC3), 6.36 (t, *J* = 2.2 Hz, 1H, PzA4), 6.34 (t, *J* = 2.3 Hz, 1H, PzB4), 6.27 (t, *J* = 2.2 Hz, 1H, PzC4), 5.50 (dd, *J* = 1.4, 9.6 Hz, 1H, H2), 4.06 (d, *J* = 9.6 Hz, 1H, H7), 3.20 (dd, *J* = 4.9, 13.3 Hz, 1H, H6), 2.83 (s, 3H, H13), 2.68 (dd, *J* = 11.4, 13.1 Hz, 1H, H6), 2.67 (ddd, *J* = 4.4, 11.6, 15.6 Hz, 1H, H4), 1.97 (s, 3H, Ms), 1.26 (d, *J* = 6.7 Hz, 3H, H14), 1.31 (m, 1H, H5), 1.25 (d, $J_{PH}$ = 8.2 Hz, 9H, $PMe_3$), 1.18 (d, *J* = 8.6 Hz, 1H, H3).

$^{13}$C NMR ($CD_3CN$, δ, 25 °C): 173.1 (C12), 144.8 (PzA3), 144.4 (PzB3), 141.3 (PzC3), 138.7 (C8), 137.4 (PzC5), 137.3 (PzB5), 137.0 (PzA5), 130.4 (2C, C9), 129.3 (2C, C10), 128.6 (C11), 107.1 (PzC4), 107.0 (PzA4), 106.9 (PzB4), 62.6 (C7), 59.1 (C2), 57.3 (C3), 52.1 (d, $J_{PC}$ = 10.7 Hz, C4), 51.6 (C13), 47.8 (C6), 40.7 (Ms), 23.0 (C5), 22.0 (C14), 14.4 (d, $J_{PC}$ = 30.0 Hz, 3C, $PMe_3$).

IR: $\nu$(NO) = 1537 cm$^{-1}$, $\nu$(CO) = 1730 cm$^{-1}$
CV (MeCN; 100 mV/s): $E_{p,a}$ = +0.50V (NHE)
SC-XRD data on S69.

**Synthesis and characterization of WTp(NO)(PMe$_3$)($\eta^2$-(*N*-mesyl)-5-succinamide-2-(methyl-*α*-phenylacetate)-1,2,5,6-tetrahydropyridine (14D)**

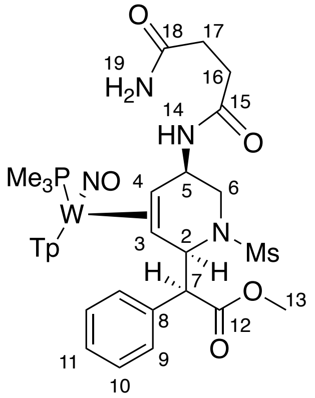


**8** (150 mg, 0.156 mmol) was dissolved in ~1 mL THF in a test tube with a stir pea. The solution was cooled to -60 °C for 15 minutes. Succinamide (155 mg, 1.56 mmol) was dissolved in 1 mL THF in a test tube with a stir pea, n-butyl lithium (1.5 M, 0.2 mL, 0.30 mmol) was added and allowed to react for 10 minutes. The solution was then cooled to -60 °C for 15 minutes. The allyl solution was added to the deprotonated succinimide solution and reacted for 60 hours at -60 °C. The solution was warmed to room temperature. The solution was diluted with DCM (10 mL), washed with $Na_2CO_3$ (3 x 5 mL), and the aqueous solution was back extracted with DCM (3 x 2 mL). The solution was dried with $Na_2SO_4$, removed on a 15 mL medium porosity frit, washed with DCM (3 x 2 mL), and the resulting filtrate was evaporated to dryness. The product was dissolved in minimal DCM and added to a stirring solution of chilled hexanes (200 mL), yielding a precipitate. This precipitate was isolated on a 15 mL fine porosity frit, washed with hexanes (10 mL), and desiccated to yield **14D** (78 mg, 54% yield).

$^1$H NMR ($CD_3CN$, δ, 25 °C): 8.47 (bs, 1H, PzA3), 8.03 (d, *J* = 2.3 Hz, 1H, PzB3), 7.86 (d, *J* =2.3 Hz, 1H, PzB5), 7.84 (d, *J* = 2.3 Hz, 1H, PzC5), 7.82 (d, *J* = 2.4 Hz, 1H, PzA5), 7.41 (d, *J* = 7.3 Hz, 2H, H9), 7.32 (t, *J* = 7.4 Hz, 2H, H10), 7.26 (t, *J* = 7.2 Hz, 1H, H11), 7.13 (d, *J* = 2.2 Hz, 1H, PzC3), 6.41 (t, *J* = 2.3 Hz, 1H, PzA4), 6.36 (t, *J* = 2.1 Hz, 1H, PzB4), 6.28 (t, *J* = 2.3 Hz, 1H, PzC4), 5.52 (d, *J* = 10.4 Hz, 1H, H2), 5.36 (m, 1H, H5), 4.42 (d, *J* = 10.3 Hz, 1H, H7), 3.53 (dd, *J* = 11.1, 12.9 Hz, 1H, H6), 3.41 (bs, 2H, H19), 3.28 (s, 1H, H14), 3.10 (dd, *J* = 6.5, 12.9 Hz, 1H, H6), 3.02 (ddd, *J* = 3.8, 12.0, 14.4 Hz, 1H, H4), 2.91 (s, 3H, 13), 2.77 (m, 4H, H16/H17), 1.85 (s, 3H, Ms), 1.05 (d, $J_{PH}$ = 8.3 Hz, 9H, PMe$_3$), 0.58 (d, *J* = 12.1 Hz, 1H, H3). Minor Complex – DHP ***erythro*-7D**.

$^{13}$C NMR ($CD_3CN$, δ, 25 °C): 178.9 (2C, C15/C18), 173.2 (C12), 145.1 (PzA3), 143.5 (PzB3), 140.7 (PzC3), 138.7 (C8), 137.5 (2C, PzB5/C5), 137.3 (PzA5), 130.3 (2C, C9), 129.6 (2C, C10), 128.9 (C11), 107.5 (PzB4), 107.3 (PzA4), 107.0 (PzC4), 62.0 (C8), 58.7 (C2), 56.6 (C3), 51.7 (C13), 50.0 (C5), 45.6 (d, $J_{PC}$ = 12.0 Hz, C4), 40.6 (Ms), 40.1 (C6), 29.0. (2C, C16/C17), 14.0. (d, $J_{PC}$ = 30.8 Hz, 3C, PMe$_3$).

IR: $\nu$(NO) = 1553 cm$^{-1}$, $\nu$(Ester CO) = 1735 cm$^{-1}$, $\nu$(Amide CO) = 1695 cm$^{-1}$
CV (MeCN; 100 mV/s): $E_{p,a}$ = +0.63V (NHE)
HRMS (ESI) m/z: [M]$^+$ Calcd for $C_{31}H_{43}BN_{10}O_6PSW^+$ due to $H_2O$ loss from McLafferty rearrangement of amide 909.2422; Found 909.2412.

**Synthesis and characterization of WTp(NO)($PMe_3$)($\eta^2$-(*N*-mesyl)-5-phthalimide-2-(methyl-*α*-phenylacetate)-1,2,5,6-tetrahydropyridine (15D)**

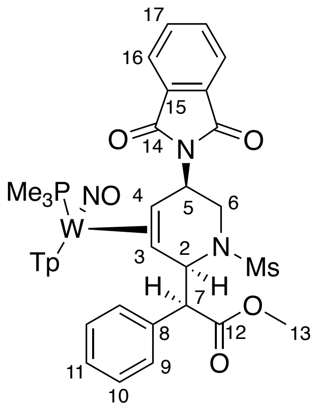


***Erythro*-7D** (50 mg, 0.062 mmol) was dissolved in ~0.5 mL propionitrile in a test tube with a stir pea. HOTf (57 mg, 0.380 mmol) was added, and the mixture was allowed to stir at room temperature. The dark brown solution was cooled at -60 °C for 15 minutes. Phthalimide (46 mg, 0.312 mmol) was dissolved in 0.5 mL EtCN in a test tube with a stir pea, tBuOK/THF (20% wt., 70 mg, 0.125 mmol) was added and allowed to react for 10 minutes. The solution was then cooled to -60 °C for 15 minutes. The allyl solution was added to the deprotonated phthalimide solution and reacted for 12 hours at -60 °C. The solution was warmed to room temperature. The solution was diluted with DCM (10 mL), washed with $Na_2CO_3$ (3 x 5 mL), and the aqueous solution was back extracted with DCM (3 x 2 mL). The solution was dried with $Na_2SO_4$, removed on a 15 mL medium porosity frit, washed with DCM (3 x 2 mL), and the resulting filtrate was evaporated to dryness. The product was dissolved in minimal DCM and added to a stirring solution of chilled hexanes (200 mL), yielding a precipitate. This precipitate was isolated on a 15 mL fine porosity frit, washed with hexanes (10mL), and desiccated to yield **15D** (42 mg, 73% yield).

$^1$H NMR ($CD_3CN$, δ, 25 °C): 8.51 (bs, 1H, PzA3), 8.07 (d, *J* = 1.8 Hz, 1H, PzB3), 7.80-7.94 (m, 4H, H16/H17), 7.75 (d, *J* = 2.5 Hz, 1H, PzB5), 7.73 (d, *J* = 2.5, 3.1 Hz, 2H, PzC5/PzA5), 7.45 (d, *J* = 7.3 Hz, 2H, H9), 7.29 (t, *J* = 7.3 Hz, 2H, H10), 7.23 (t, *J* = 6.3 Hz, 1H, H11), 7.10 (d, *J* = 2.1 Hz, 1H, PzC3), 6.40 (t, *J* = 2.1 Hz, 1H, PzA4), 6.32 (t, *J* = 2.3 Hz, 1H, PzB4), 6.20 (t, *J* = 2.3 Hz, 1H, PzC4), 5.57 (d, *J* = 10.3 Hz, 1H, H2), 5.55 (m, 1H, H5), 4.52 (d, *J* = 10.1 Hz, 1H, H7), 3.58 (dd, *J* = 10.8, 12.7 Hz, 1H, H6), 3.25 (dd, *J* = 6.7, 12.8 Hz, 1H, H6), 3.11 (ddd, *J* = 3.8, 12.1, 14.3 Hz, 1H, H4), 2.98 (s, 3H, H13), 1.92 (bs, 3H, Ms), 1.02 (d, $J_{PH}$ = 8.4 Hz, 9H, $PMe_3$), 0.71 (d, *J* = 11.8 Hz, 1H, H3). Minor Complex – DHP ***erythro*-7D**.

$^{13}$C NMR ($CD_3CN$, δ, 25 °C): 172.7 (C12), 168.7 (2C, C14), 144.9 (PzA3A), 142.8 (PzB3), 139.8 (PzC3), 138.1 (C8), 136.6 (PzA5), 136.6 (PzC5), 136.3 (PzB5), 134.7 (2C, C16), 132.3 (2C, C15), 129.9 (2C, C9), 129.0 (2C, C10), 128.3 (C11), 123.7 (2C, C17), 106.8 (PzB4), 106.5 (PzC4), 106.5 (PzC4), 61.5 (C7), 58.4 (C2), 56.1 (C3), 51.5 (C13), 49.0 (C5), 45.7 (d, $J_{PC}$ = 11.7 Hz, C4), 40.8 (C6), 40.7 (Ms), 13.9 (d, $J_{PC}$ = 28.6 Hz, $PMe_3$).

IR: $\nu$(NO) = 1557 $cm^{-1}$, $\nu$(Ester CO) = 1732 $cm^{-1}$ , $\nu$(Imide CO) = 1705 $cm^{-1}$
CV (DMA; 100 mV/s): $E_{p,a}$ = +0.58V (NHE)
HRMS (APCI) m/z: $[M]^+$ Calcd for $C_{35}H_{43}BN_9O_7PSW^+$ 958.2262; Found 958.2270.

**Synthesis and characterization of WTp(NO)($PMe_3$)($\eta^2$-(*N*-mesyl)-5-imidazole-2-(methyl-$\alpha$-phenylacetate)-1,2,5,6-tetrahydropyridine (16D)**

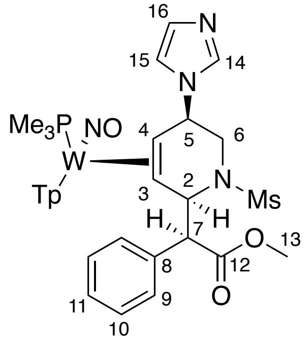

***Erythro*-7D** (250 mg, 0.308 mmol) was dissolved in 1 mL propionitrile in a test tube with a stir pea. HOTf (55.5 mg, 0.370 mmol) was added to form the allyl species, and the mixture was allowed to stir at room temperature. The dark brown solution was cooled at -30 °C for 15 minutes. Imidazole (210 mg, 3.08 mmol) was dissolved in 1.5 mL methanol in a test tube with a stir pea and cooled to -30 °C for 15 minutes. The allyl solution was added to the imidazole solution and reacted for 12 hours at -30 °C. tBuOK/THF (20% wt, 346 mg, 0.617 mmol) was added at -30 °C and allowed to react for 10 minutes. The solution was warmed to room temperature. The solution was diluted with DCM (10 mL), washed with $Na_2CO_3$ (3 x 5 mL), and the aqueous solution was back extracted with DCM (3 x 2mL). The solution was dried with $Na_2SO4$, removed on a 15 mL medium porosity frit, washed with DCM (3 x 2 mL), and the resulting filtrate was evaporated to dryness. The product was dissolved in minimal DCM and added to a stirring solution of hexanes (200 mL), yielding a precipitate. This precipitate was isolated on a 15 mL fine porosity frit, washed with hexanes (10 mL), and desiccated to yield **16D** (232 mg, 86% yield).

$^1$H NMR ($CD_3CN$, δ, 25 °C): 8.47 (bs, 1H, PzA3), 8.03 (d, *J* = 1.7 Hz, 1H, PzB3), 7.85 (bs, 1H, H14), 7.76 (d, *J* = 2.4 Hz, 1H, PzB5), 7.75 (d, *J* = 2.1 Hz, 1H, PzC5), 7.73 (d, *J* = 2.3 Hz, 1H, PzA5), 7.43 (bs, 1H, H15), 7.40 (d, *J* = 7.3 Hz, 2H, H9), 7.29 (t, *J* = 7.4 Hz, 2H, H10), 7.24 (t, *J* = 7.3 Hz, 1H, H11), 7.15 (bs, 1H, H16), 7.10 (d, *J* = 2.1 Hz, 1H, PzC3), 6.40 (d, *J* = 2.2 Hz, 1H, PzA4), 6.33 (t, *J* = 2.1 Hz, 1H, PzB4), 6.25 (t, *J* = 2.1 Hz, 1H, PzC4), 5.62 (dd, *J* = 1.7, 10.0 Hz, 1H, H2), 5.30 (m, 1H, H5), 4.14 (d, *J* = 10.0 Hz, 1H, H7), 3.47 (dd, *J* = 5.2, 13.1 Hz, 1H, H6), 3.05 (dd, *J* = 11.2, 13.1 Hz, 1H, H6), 2.94 (ddd, *J* = 4.6, 11.6, 14.0 Hz, 1H, H4), 2.88 (s, 3H, 13), 1.76 (s, 3H, Ms), 0.86 (d, $J_{PH}$ = 8.4 Hz, 9H, $PMe_3$), 0.65 (d, *J* = 12.2 Hz, 1H, H3). Minor Complex – DHP ***erythro*-7D**.

$^{13}$C NMR ($CD_3CN$, δ, 25 °C): 172.2 (C12), 144.8 (PzA3), 143.0 (PzB3), 139.7 (PzC3), 137.9 (C8), 137.1 (PzB5), 136.8 (C14), 136.7 (PzC5), 136.5 (PzA5), 130.2 (C16), 129.6 (2C, C9), 129.2 (2C, C10), 128.5 (C11), 118.2 (C15), 106.9 (PzB4), 106.6 (PzA4), 106.6 (PzC4), 62.0 (C7), 58.5 (C2), 56.2 (C5), 54.5 (C3), 51.5 (C13), 48.6 (d, $J_{PC}$ = 12.6 Hz, C4), 47.2 (C6), 40.7 (Ms), 13.6 (d, $J_{PC}$ = 28.4 Hz, 3C, $PMe_3$).

IR: $\nu$(NO) = 1563 cm$^{-1}$, $\nu$(Ester CO) = 1728 cm$^{-1}$
CV (DMA; 100 mV/s): $E_{p,a}$ = +0.57V (NHE)
HRMS (APCI) m/z: [M]$^+$ Calcd for $C_{30}H_{41}BN_{10}O_5PSW^+$ 879.2317; Found 879.2318.

**Synthesis and characterization of WTp(NO)($PMe_3$)($\eta^2$-(*N*-mesyl)-5-tryptamine -2-(methyl-*α*-phenylacetate)-1,2,5,6-tetrahydropyridine (17D)**

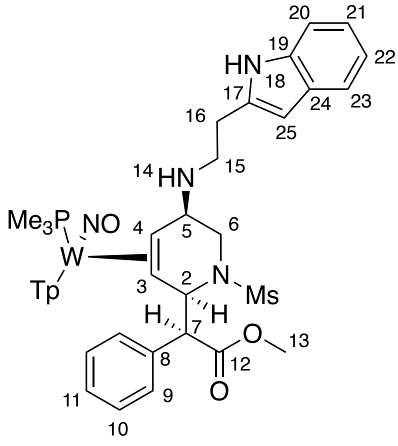


***Erythro*-7D** (100 mg, 0.123 mmol) was dissolved in ~0.5 mL propionitrile in a test tube with a stir pea. HOTf (24 mg, 0.160 mmol) was added, and the mixture was allowed to stir at room temperature. The dark brown solution was cooled at -50 °C for 15 minutes. Tryptamine (85 mg, 0.531 mmol) was dissolved in 0.5 mL EtCN in a test tube with a stir pea, tBuOK/THF (20% wt., 114 mg, 0.203 mmol) was added and allowed to react for 10 minutes. The solution was then cooled to -50 °C for 15 minutes. The allyl solution was added to the deprotonated tryptamine solution and reacted for 12 hours at -50 °C. The solution was warmed to room temperature. The solution was diluted with DCM (10 mL), washed with $Na_2CO_3$ (3 x 5 mL), and the aqueous solution was back extracted with DCM (3 x 2mL). The solution was dried with $MgSO_4$, removed on a 15 mL medium porosity frit, washed with DCM (3 x 2 mL), and the resulting filtrate was evaporated to dryness. The product was dissolved in minimal DCM and added to a stirring solution of chilled pentanes (200 mL), yielding a precipitate. This precipitate was isolated on a 15 mL fine porosity frit, washed with pentanes (10 mL), and desiccated to yield **17D** (103 mg, 88% yield).

$^1$H NMR ($CD_2Cl_2$, δ, 25 °C): 8.52 (bs, 1H, H18), 8.38 (d, *J* = 1.5 Hz, 1H, PzA3), 8.01 (d, *J* = 1.9 Hz, 1H, PzB3), 7.73 (d, *J* = 2.3 Hz, 1H, PzB5), 7.71 (d, *J* = 2.2 Hz, 1H, PzC5), 7.68 (d, *J* = 2.4 Hz, 1H, PzA5), 7.64 (d, *J* = 7.8 Hz, 1H, H23), 7.38 (d, *J* = 8.0 Hz, 1H, H20), 7.36 (d, *J* = 7.2 Hz, 2H, H9), 7.25 (t, *J* = 7.2 Hz, 2H, H10), 7.21 (t, *J* = 7.2 Hz, 1H, H11), 7.16 (t, *J* = 7.9 Hz, 1H, H22), 7.11 (d, *J* = 7.2 Hz, PzC3), 7.09 (t, *J* = 8.1 Hz, 1H, H21), 7.07 (d, *J* = 2.2 Hz, 1H, H25), 6.32 (t, *J* = 2.3 Hz, 1H, PzA4), 6.28 (t, *J* = 2.0 Hz, 1H, PzB4), 6.20 (t, *J* = 2.2 Hz, 1H, PzC4), 5.50 (d, *J* = 9.4 Hz, 1H, H2), 4.02 (d, *J* = 9.5 Hz, 1H, H7), 3.56-3.83 (m, 1H, H5), 3.64 (dd, *J* = 4.8, 12.6 Hz, 1H, H6), 3.27 (dt, *J* = 6.8, 13.8, 1H, H15), 3.00 (td, *J* = 6.47, 13.83 Hz, 1H, H17), 2.98 (td, *J* = 6.87, 13.95 Hz, 1H, H17), 2.88 (dt, *J* = 6.7, 10.9 Hz, 1H, H15), 2.84 (s, 3H, H9), 2.47-2.42 (m, 2H, H6/H4), 1.92 (s, 3H, H7), 1.43 (bs, 1H, H14), 1.18 (d, $J_{PH}$ = 8.7 Hz, 9H, $PMe_3$), 0.47 (d, *J* = 11.7 Hz, 1H, H3).

$^{13}$C NMR ($CD_2Cl_2$, δ, 25 °C): 172.5 (C12), 144.4 (PzA3), 143.6 (PzB3), 140.1 (PzC3), 138.0 (C8) 136.9 (C24), 136.5 (PzB5), 136.4 (PzC5), 136.0 (PzA5), 129.9 (2C, C9), 128.8 (2C, C10), 128.1 (C11), 128.1 (C19), 122.7 (C25), 122.0 (2C, C22), 119.3 (2C, C21), 119.2 (2C, C23), 111.6 (2C, C20), 114.4 (C17), 106.4 (PzB4), 106.3 (PzA4), 106.2 (PzC4), 62.2 (C7), 58.9 (C2), 56.5 (C5), 54.9 (C3), 51.8 (d, $J_{PC}$ = 13.4 Hz, C4), 51.2 (C13), 47.4 (C15), 46.0 (C6), 40.5 (Ms), 27.1 (C16), 14.2 (d, $J_{PC}$ = 28.9 Hz, 3C, $PMe_3$).

IR: $\nu$(NO) = 1563 cm$^{-1}$, $\nu$(Ester CO) = 1725 cm$^{-1}$
CV (DMA; 100 mV/s): $E_{p,a}$ = +0.59V (NHE)
HRMS (APCI) m/z: [M]$^+$ Calcd for $C_{37}H_{49}BN_{10}O_5PSW^+$ 971.2943; Found 971.2922.

**Synthesis and characterization of WTp(NO)(PMe$_3$)($\eta^2$-(*N*-mesyl)-5-benzotriazole -2-(methyl-$\alpha$-phenylacetate)-1,2,5,6-tetrahydropyridine (18D)**

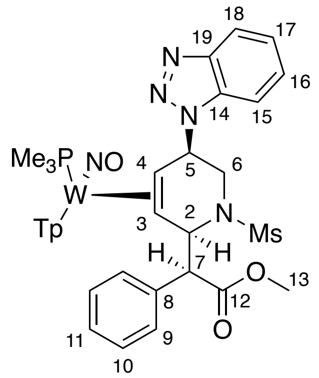

***Erythro*-7D** (50 mg, 0.062 mmol) was dissolved in 1ml propionitrile in a test tube with a stir pea. HOTf/MeCN (0.6 M, 260 mg, 0.093mmol) was added, and the mixture was allowed to stir at room temperature. The dark brown solution was cooled at -40 °C for 15 minutes. Benzotriazole (40 mg, 0.311 mmol) was dissolved in 0.5 mL THF in a test tube with a stir pea, tBuOK/THF (20% wt, 104 mg, 0.185 mmol) as added and allowed to react for 10 minutes. The solution was then cooled to -40 °C for 15 minutes. The allyl solution was added to the deprotonated benzotriazole solution and reacted for 12 hours at -40 °C. The solution was warmed to room temperature. The solution was diluted with DCM (10 mL), washed with $Na_2CO_3$ (3 x 5 mL), and the aqueous solution was back extracted with DCM (3 x 2mL). The solution was dried with $MgSO_4$, removed on a 15 mL medium porosity frit, washed with DCM (3 x 2 mL), and the resulting filtrate was evaporated to dryness. The product was dissolved in minimal DCM and added to a stirring solution of hexanes (75 mL), yielding a precipitate. This precipitate was isolated on a 15 mL fine porosity frit, washed with hexanes (10 mL), and desiccated to yield **18D** (31 mg, 54% yield).

$^1$H NMR ($CD_2Cl_2$, δ, 25 °C): 8.52 (bs, 1H, PzA3), 8.07 (d, $J$ = 1.6 Hz, 1H, PzB3), 7.96 (d, 2H, H15/H18), 7.77 (d, $J$ = 2.3 Hz, 1H, PzB5), 7.74-7.76 (m, 2H, PzA5/C5), 7.45 (m, 4H, H9/H16/H17), 7.31 (t, $J$ = 7.6 Hz, 2H, H10), 7.25 (t, $J$ = 7.3 Hz, 1H, H11), 7.15 (d, $J$ = 1.8 Hz, 1H, PzC3), 6.43 (t, $J$ = 2.2 Hz, 1H, PzA4), 6.33 (t, $J$ = 2.2 Hz, 1H, PzB4), 6.23 (t, $J$ = 2.2 Hz, 1H, PzC4), 6.09 (ddd, $J$ = 4.5, 10.2, 15.4 Hz, 1H, H5), 5.67 (d, $J$ = 10.1 Hz, 1H, H2), 4.23 (d, $J$ = 10.1 Hz, 1H, H7), 3.63 (dd, $J$ = 5.1, 12.9 Hz, 1H, H6), 3.56 (ddd, $J$ = 4.7, 11.6, 14.6 Hz, 1H, H4), 3.50 (dd, $J$ = 11.0, 12.8 Hz, 1H, H6), 2.90 (s, 3H, H13), 1.79 (s, 3H, Ms), 0.78 (d, $J_{PH}$ =8.3 Hz, 9H, PMe$_3$), 0.68 (d, $J$ = 7.7 Hz, 1H, H3).

$^{13}$C NMR ($CD_2Cl_2$, δ, 25 °C): 172.1 (C12), 144.8 (4C, PzA3/C14/C19), 143.2 (PzB3), 140.1 (PzC3), 138.0 (C8), 136.7 (2C, PzA5/B5/C5), 136.4 (PzA5/B5/C5), 129.7 (2C, C9), 129.1 (2C, C10), 128.4 (C11), 126.7 (2C, C16/C17), 118.5 (2C, C15/C18), 106.8 (PzB4), 106.6 (PzA4), 106.5 (PzC4), 68.1, (C5), 65.6 (C7), 62.0 (C2), 58.9 (C3), 51.5 (C13), 47.8 (C4), 46.0 (C6), 40.7 (Ms), 13.5 (d, $J_{PC}$ = 8.4 Hz, 3C, PMe$_3$).

IR: $\nu$(NO) = 1568 cm$^{-1}$, $\nu$(Ester CO) = 1731 cm$^{-1}$
CV (DMA; 100 mV/s): $E_{p,a}$ = +0.67 V (NHE)
HRMS (APCI) m/z: [M]$^+$ Calcd for $C_{33}H_{42}BN_{11}O_5PSW^+$ 930.2426; Found 930.2438.

**Synthesis and characterization of WTp(NO)($PMe_3$)($\eta^2$-(*N*-mesyl)-5-pentyl sulfide-2-(methyl-$\alpha$-phenylacetate)-1,2,5,6-tetrahydropyridine (19D)**

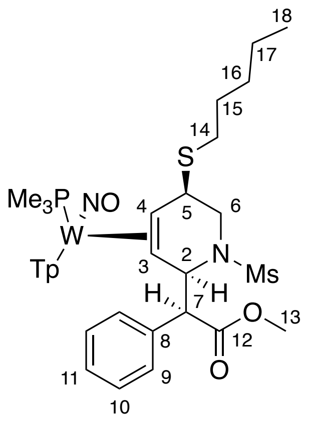


***Erythro*-7D** (100 mg, 0.123 mmol) was dissolved in 1 mL propionitrile in a test tube with a stir pea. HOTf/MeCN (30 mg, 0.200 mmol) was added, and the mixture was allowed to stir at room temperature. The dark brown solution was cooled at -50 °C for 15 minutes. Pentanethiol (90 mg, 0.864 mmol) was dissolved in 0.5 mL THF in a test tube with a stir pea, tBuOK/THF (20% wt, 208 mg, 0.371 mmol) was added and allowed to react for 10 minutes. The solution was then cooled to -50 °C for 15 minutes. The allyl solution was added to the deprotonated pentanethiol solution and reacted for 12 hours at -50 °C. The solution was warmed to room temperature. The solution was diluted with DCM (10 mL), washed with $Na_2CO_3$ (3 x 5 mL), and the aqueous solution was back extracted with DCM (3 x 2 mL). The solution was dried with $Na_2SO_4$, removed on a 15 mL medium porosity frit, washed with DCM (3 x 2 mL), and the resulting filtrate was evaporated to dryness. The product was dissolved in minimal DCM and added to a stirring solution of chilled stirring pentanes (75 mL), yielding a precipitate. This precipitate was isolated on a 15 mL fine porosity frit, washed with pentanes (10 mL), and desiccated to yield **19D** (44 mg, 40% yield).

$^1$H NMR (($(CD_3)_2CO$, δ, 25 °C): 8.45 (bs, 1H, PzA3), 8.09 (d, *J* = 1.5 Hz, 1H, PzB3), 7.94 (d, *J* = 2.1 Hz, 1H, PzC5), 7.92 (d, *J* = 2.4 Hz, 1H, PzB5), 7.82 (d, *J* = 2.4 Hz, 1H, PzA5), 7.42 (d, *J* = 7.4 Hz, 2H, H9), 7.31 (d, *J* = 1.5 Hz, 1H, PzC3), 7.25 (t, *J* = 7.2 Hz, 2H, H10), 7.22 (t, *J* = 7.5 Hz, 1H, H11), 6.37 (t, *J* = 2.2 Hz, 1H, PzB4), 6.34 (m, 2H, PzC4/PzA4), 5.57 (dd, *J* = 1.7, 9.4 Hz, 1H, H2), 4.1 (d, *J* = 9.6 Hz, 1H, H7), 3.93 (ddd, *J* = 5.4, 10.3, 15.7 Hz, 1H, H5), 3.61 (dd, *J* = 4.9, 13.4 Hz, 1H, H6), 3.03 (dd, *J* = 10.9, 13.4 Hz, 1H, H6), 2.87 (s, 3H, H13), 2.72 (m, 3H, H4/H14), 2.00 (s, 3H, Ms), 1.74-1.64 (m, 2H, H15), 1.48-1.41 (m, 2H, H16/H17), 1.41-1.34 (m, 2H, H16/H17), 1.38 (d, $J_{PH}$ = 8.70 Hz, 9H, $PMe_3$), 0.92 (t, *J* = 7.3 Hz, 3H, H18), 0.58 (d, *J* = 11.5 Hz, 1H, H3).

$^{13}$C NMR (($(CD_3)_2CO$, δ, 25 °C): 172.6 (C12), 145.1 (PzA3), 144.3 (PzB3), 141.4 (PzC3), 138.9 (C8), 137.4 (PzC5), 137.3 (PzB5), 137.0 (PzA5), 130.6 (2C, C9), 129.3 (2C, C10), 128.5 (C11), 107.12/107.08 (PzC4/PzA4), 62.8 (C7), 59.1 (C2), 55.8 (C3), 51.4 (C13), 48.1 (d, $J_{PC}$ = 14.0 Hz, C4), 47.3 (C6), 43.9 (C5), 41.0 (Ms), 32.2/23.1 (2C, C16/C17), 31.7 (C14), 30.6 (C15), 14.4 (C18), 14.3 (d, $J_{PC}$ = 28.8 Hz, 3C, $PMe_3$).

IR: $\nu$(NO) = 1567 cm$^{-1}$, $\nu$(Ester CO) = 1732 cm$^{-1}$
CV (DMA; 100 mV/s): $E_{p,a}$ = +0.54V (NHE)
HRMS (APCI) m/z: [M]$^+$ Calcd for $C_{32}H_{49}BN_8O_5PS_2W^+$ 915.2602; Found 915.2581.
SC-XRD data on S70.

**Synthesis and characterization of WTp(NO)($PMe_3$)($\eta^2$-(*N*-mesyl)-5-benzyl sulfide-2-(methyl-*α*-phenylacetate)-1,2,5,6-tetrahydropyridine (20D)**

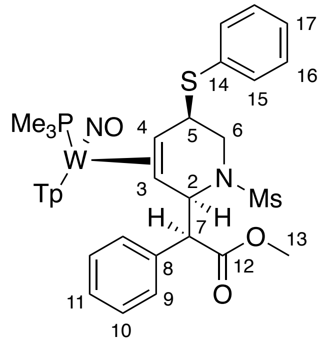


***Erythro*-7D** (100 mg, 0.123 mmol) was dissolved in 1ml propionitrile in a test tube with a stir pea. HOTf/MeCN (30 mg, 0.200 mmol) was added, and the mixture was allowed to stir at room temperature. The dark brown solution was cooled at -40 °C for 15 minutes. Benzenethiol (100 mg, 0.908 mmol) was dissolved in 0.5 mL THF in a test tube with a stir pea, tBuOK/THF (20% wt, 208 mg, 0.371 mmol) was added and allowed to react for 10 minutes. The solution was then cooled to -40 °C for 15 minutes. The allyl solution was added to the deprotonated benzenethiol solution and reacted for 12 hours at -60 °C. The solution was warmed to room temperature. The solution was diluted with DCM (10 mL), washed with $Na_2CO_3$ (3 x 5 mL), and the aqueous solution was back extracted with DCM (3 x 2 mL). The solution was dried with $MgSO_4$, removed on a 15 mL medium porosity frit, washed with DCM (3 x 2 mL), and the resulting filtrate was evaporated to dryness. The product was dissolved in minimal DCM and added to a stirring solution of hexanes (75 mL), yielding a precipitate. This precipitate was isolated on a 15 mL fine porosity frit, washed with hexanes (10 mL), and desiccated to yield **20D** (34 mg, 31% yield).

$^1$H NMR (($(CD_3)_2CO$, δ, 25 °C): 8.53 (d, *J* = 1.5 Hz, 1H, PzA3), 8.10 (d, *J* = 2.2 Hz, 1H, PzB3), 7.97 (d, *J* = 2.2 Hz, 1H, PzC5), 7.94 (d, *J* = 2.2 Hz, 1H, PzB5), 7.85 (d, *J* = 2.8 Hz, 1H, PzA5), 7.48 (d, *J* = 8.7 Hz, 2H, H15), 7.44 (d, *J* = 7.7 Hz, 2H, H9), 7.37 (d, *J* = 2.2 Hz, 1H, PzC3), 7.32 (t, *J* = 7.7 Hz, 2H, H10), 7.26 (t, *J* = 7.7 Hz, 2H, H16), 7.23 (t, *J* = 7.2 Hz, 1H, H11), 7.19 (t, *J* = 7.0 Hz, 1H, H17), 6.38-6.37 (m, 2H, PzA4/PzB4), 6.36 (t, *J* = 2.2 Hz, 1H, PzC4), 5.60 (d, *J* = 9.8 Hz, 1H, H2), 4.60 (m, *J* = 4.5, 9.9 Hz, 1H, H5), 4.14 (d, *J* = 9.7 Hz, 1H, H7), 3.57 (dd, *J* = 4.7, 13.6 Hz, 1H, H6), 3.06 (dd, *J* = 10.8, 13.6 Hz, 1H, H6), 2.85 (s, 3H, H13), 2.77 (m, 1H, H4), 1.83 (s, 3H, Ms), 1.36 (d, $J_{PH}$ = 8.5 Hz, 9H, $PMe_3$), 0.59 (d, *J* = 10.9 Hz, 1H, H3).

$^{13}$C NMR (($CD_2Cl_2$), δ, 25 °C): 172.2 (C12), 144.8 (PzA3), 143.7 (PzB3), 140.3 (PzC3), 138.1/136.4 (2C, C8/C14), 136.7 (PzC5), 136.7 (PzB5), 136.3 (PzA5), 129.0 (2C, C15), 129.7 (2C, C9), 129.6 (2C, C10), 128.9 (2C, C16), 128.3 (C11), 126.2 (C17), 106.5/106.4 (2C, PzA4/PzB4), 106.4 (PzC4), 62.0 (C7), 58.8 (C2), 55.6 (C3), 51.3 (C13), 46.5 (m, 2C, C4/C6), 43.60 (C5), 40.6 (Ms), 14.5 (d, $J_{PC}$ = 30.8 Hz, 3C, $PMe_3$).

IR: $\nu$(NO) = 1559 cm$^{-1}$, $\nu$(Ester CO) = 1732 cm$^{-1}$
CV (DMA; 100 mV/s): $E_{p,a}$ = +0.59V (NHE)
HRMS (APCI) m/z: [M]$^+$ Calcd for $C_{33}H_{44}BN_8O_5PS_2W^+$ 921.2132; Found 921.2136

**Synthesis and characterization of (*N*-mesyl)-2-(methyl-*α*-phenylacetate)-1,2,5,6-tetrahydropyridine (21-Ms)**

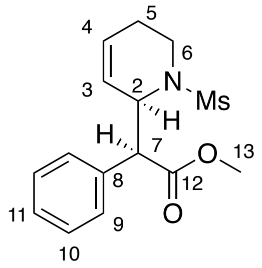


Acetone (3 mL), 2,3-dichloro-5,6-dicyano-1,4-benzoquinone (DDQ) (130 mg, 0.573 mmol), and **9D** (107 mg, 0.132 mmol) were added to a 4-dram vial containing a stir pea. The reaction was stirred in a fume hood for 4 hours. The solution was flushed through a 1 cm basic alumina plug set up in a 30 mL coarse porosity frit. This solution was diluted with 30 mL of DCM and was washed 3x with 50 mL of saturated aqueous $NaHCO_3$. The organic layer was isolated and set aside. The combined aqueous layers were combined and back-extracted with 30 mL of DCM to prevent loss of product. The organic layers were combined in a single round-bottom flask to which 5 grams of basic alumina powder were added. The solution was then reduced off *in vacuo*, bringing the eluted organic into the basic alumina powder. This powder was then dry loaded onto an 8-gram Teledyne basic alumina column, upon which clean product was eluted off of with 40% ethyl acetate in hexanes. The tubes containing organic were combined and reduced *in vacuo*, yielding a white residue (20 mg, 49% yield).

$^1$H NMR (($(CD_3)_2CO$, δ, 25 °C): 7.45 (d, *J* = 7.2 Hz, 2H, H9), 7.36 (t, *J* = 7.8 Hz, 2H, H10), 7.31 (tt, *J* = 2.0, 7.4 Hz, 1H, H11), 5.95-5.98 (m, 1H, H4), 5.85-5.88 (m, 1H, H3), 4.90 (d, *J* = 10.7 Hz, 1H, H2), 3.89 (d, *J* = 10.7 Hz, 1H, H7), 3.68 (s, 3H, H13), 3.60 (ddd, *J* = 0.9, 6.5, 14.6 Hz, 1H, H6), 3.26 (ddd, *J* = 4.4, 11.9, 14.50 Hz, 1H, H6), 2.31-2.37 (m, 1H, H5), 2.06 (s, 3H, Ms), 2.00 (dt, *J* = 4.9 18.1 Hz, 1H, H5).

$^{13}$C NMR (($(CD_3)_2CO$ δ, 25 °C): 172.8 (C12), 137.6 (C8), 129.8 (2C, C9), 129.4 (2C, C10), 128.7 (C11), 128.5 (C4), 127.3 (C3), 56.5 (C7), 56.1 (C2), 52.5 (C13), 39.8 (Ms), 39.0 (C6), 24.7 (C5).

SC-XRD data on S71.

**Synthesis and characterization of (*N*-acetyl)-2-(methyl-*α*-phenylacetate)-1,2,5,6-tetrahydropyridine (21-Ac)**

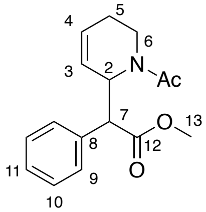


**9D-Ac** (90 mg, 0.12 mmol) was dissolved in MeCN (3.0 mL) in a 4 dram vial containing a stir pea. Separately, DDQ (50 mg, 0.22 mmol) was dissolved in MeCN (1.0 mL). The DDQ solution was added to the solution of **9D-Ac**, and the resulting mixture was stirred for 5 min. The reaction mixture was then diluted with DCM (50 mL) and extracted with saturated aqueous $NaHCO_3$ (50 mL). The aqueous layer was back-extracted with DCM (15 mL), and the combined organic layers were dried with $Na_2SO_4$. The solids were filtered off on a 30 mL medium fritted funnel, and rinsed with DCM (10 mL). The filtrate was evaporated on a rotary evaporator. The residue was dissolved in DCM (2 mL) and added to stirring $Et_2O$ (100 mL). The precipitate was filtered off on a 30 mL medium porosity fritted funnel and rinsed with $Et_2O$ (10 mL). The filtrate was evaporated onto silica on a rotary evaporator. The desired product was purified by Combiflash flash chromatography using a 0-100% EtOAc in hexanes gradient 30 mL/min elution on a 12 g silica column. The desired product eluted at ~48% EtOAc. The fractions containing the product were combined and evaporated on a rotary evaporator. The residue was desiccated under high vacuum for 30 min to yield compound **21-Ac** as a colorless oil (22 mg, 66%). Full synthetic details, characterization, and related crystal structures of 9D-Ac and its precursors. are found in Reference 1, pages 145-156.[1]

*Erythro* isomer: A mixture of two rotamers **A**:**B** = 4:3. $^{1}$H NMR ($CDCl_3$, δ, 25 °C): **A** 7.45-7.25 (m, 5H, H9-H11), 6.00 (m, 1H, H4), 5.88 (m, 1H, H3), 4.72 (dd, *J* =13.1, 6.2 Hz, 1H, H6), 4.60 (m, 1H, H2), 3.87 (d, *J* = 10.4 Hz, 1H, H7), 3.76 (s, 3H, H13), 2.83 (m, 1H, H6'), 2.27 (m, 1H, H5), 2.04 (dt, *J* = 5.0, 18.1 Hz, 1H, H5'), 1.44 (s, 3H, *N*-Ac). **B** 7.45-7.25 (m, 5H, H9-H11), 5.88 (m, 1H, H4), 5.85 (m, 1H, H3), 5.60 (m, 1H, H2), 3.89 (d, *J* = 9.4 Hz, 1H, H7), 3.64 (dd, *J* = 13.9, 5.9 Hz, 1H, H6), 3.72 (s, 3H, H13), 3.22 (ddd, *J* = 4.2, 12.9, 13.9 Hz, 1H, H6'), 2.27 (m, 1H, H5), 1.96 (dt, *J* = 5.0, 18.3 Hz, 1H, H5'), 1.90 (s, 3H, *N*-Ac).

$^{13}$C NMR ($CDCl_3$, δ): **A** 172.0 (C12), 170.0 (*N*-Ac), 135.3 (C8), 129.1 (C9), 128.5 (C10), 127.8 (C11), 129.2 (C4), 126.2 (C3), 57.2 (C2), 55.3 (C7), 52.4 (C13), 34.8 (C6), 24.9 (C5), 20.9 (*N*-Ac). **B** 172.4 (C12), 169.1 (*N*-Ac), 134.8 (C8), 128.8 (C9), 128.4 (C10), 127.8 (C11), 126.6 (C3), 126.2 (C4), 55.3 (C7), 52.4 (C13), 51.6 (C2), 40.3 (C6), 24.5 (C5), 21.6 (*N*-Ac).

*Threo* isomer: A mixture of two rotamers **A**:**B** = 1:1. $^{1}$H NMR ($CDCl_3$, δ): **A** 7.45-7.25 (m, 5H, H9-H11), 5.85 (m, 1H, H4), 5.27 (m, 1H, H3), 4.79 (m, 1H, H2), 4.66 (dd, *J* = 6.4, 13.5 Hz, 1H, H6), 3.94 (d, *J* = 10.2 Hz, 1H, H7), 3.69 (s, 3H, H13), 2.82 (m, 1H, H6'), 2.32 (s, 3H, *N*-Ac), 2.22 (m, 1H, H5), 2.08 (dt, *J* = 4.4, 17.2 Hz, 1H, H5'). **B** 7.45-7.25 (m, 5H, H9-H11), 5.85 (m, 1H, H4), 5.69 (m, 1H, H3), 5.54 (m, 1H, H2), 4.07 (d, *J* = 7.8 Hz, 1H, H7), 3.67 (s, 3H, H13), 3.54 (dd, *J* = 5.8, 13.8 Hz, 1H, H6), 2.63 (ddd, *J* = 3.9, 12.7, 14.1 Hz, 1H, H6'), 2.13 (s, 3H, *N*-Ac), 2.13 (m, 1H, H5), 1.90 (buried, 1H, H5').

$^{13}$C NMR ($CDCl_3$, δ): **A** 172.3 (C12), 170.6 (*N*-Ac), 135.3 (C8), 129.0 (C9), 128.6 (C4), 128.4 (C10), 128.3 (C11), 125.6 (C3), 57.3 (C2), 54.5 (C7), 52.3 (C13), 35.1 (C6), 24.6 (C5), 21.7 (*N*-Ac). **B** 172.5 (C12), 169.4 (*N*-Ac), 135.3 (C8), 129.9 (C9), 128.8 (C10), 128.4 (C11), 127.3 (C4), 126.2 (C3), 56.5 (C7), 52.3 (C2), 52.2 (C13), 40.5 (C6), 25.3 (C5), 22.1 (*N*-Ac).

ESI-MS: obs'd (%), calc'd (%), ppm, $(M+H)^+$: 274.1439 (100), 274.1438 (100), 0.4.
SC-XRD data on S72.

**Synthesis and characterization of (*N*-tosyl)-2-(methyl-*α*-phenylacetate)-1,2,5,6-tetrahydropyridine (21-Ts)**

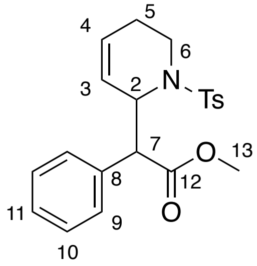


**9D-Ts** (70 mg, 0.0788 mmol) was dissolved in MeCN (3.0 mL) in a 4 dram vial containing a stir pea. Separately, DDQ (40 mg, 0.176 mmol) was dissolved in MeCN (1.0 mL). The DDQ solution was added to the solution of **9D-Ts**, and the resulting mixture was stirred for 5 min. The reaction mixture was then diluted with DCM (50 mL) and extracted with saturated aqueous $NaHCO_3$ (50 mL). The aqueous layer was back-extracted with DCM (15 mL), and the combined organic layers were dried with $Na_2SO_4$. The solids were filtered off on a 30 mL medium fritted funnel, and rinsed with DCM (10 mL). The filtrate was evaporated on a rotary evaporator. The residue was dissolved in DCM (2 mL) and added to stirring $Et_2O$ (100 mL). The precipitate was filtered off on a 30 mL medium porosity fritted funnel and rinsed with $Et_2O$ (10 mL). The filtrate was evaporated onto silica on a rotary evaporator. The desired product was purified by Combiflash flash chromatography using a 0-100% EtOAc in hexanes gradient 30 mL/min elution on a 12 g silica column. The desired product eluted at ~48% EtOAc. The fractions containing the product were combined and evaporated on a rotary evaporator. The residue was desiccated under high vacuum for 30 min to yield compound **21-Ts** as a colorless oil which spontaneously crystallized (19 mg, 62%). Full synthetic details, characterization, and related crystal structures of 9D-Ts and its precursors are found in Reference 1, pages 145-156.[1]

Two diastereomers **A**:**B**.
$^{1}$H NMR ($CDCl_3$, δ, 25 °C): **A** 7.72 (d, *J* = 8.3 Hz, 2H, Ts), 7.36-7.28 (m, 5H, H9-H11), 7.25 (d, *J* = 8.3 Hz, 2H, Ts), 5.63 (m, 1H, H4), 5.54 (m, 1H, H3), 5.03 (m, 1H, H2), 4.00 (d, *J* = 8.2 Hz, 1H, H7), 3.68 (dd, *J* = 14.8, 6.4 Hz, 1H, H6), 3.66 (s, 3H, H13), 2.69 (dd, *J* = 14.8, 4.6Hz, 1H, H6'), 2.40 (s, 3H, Ts), 1.71 (m, 1H, H5), 1.58 (ddd, *J* = 18.0, 6.4, 4.6 Hz, 1H, H5'). **B** 7.36-7.28 (m, 5H, H9-H11), 7.23 (d, *J* = 8.3 Hz, 2H, Ts), 7.07 (d, *J* = 8.3 Hz, 2H, Ts), 6.84 (m, 1H, H3), 5.79 (m, 1H, H4), 5.03 (m, 1H, H2), 3.80 (d, *J* = 9.8 Hz, 1H, H7), 3.69 (s, 3H, H13), 3.54 (dd, *J* = 14.8, 6.4 Hz, 1H, H6), 3.07 (ddd, *J* = 14.8, 12.0 Hz, 4.6 Hz, 1H, H6'), 2.35 (s, 3H, Ts), 2.06 (m, 1H, H5), 1.80 (ddd, *J* = 18.0, 6.4, 4.6 Hz, 1H, H5').

$^{13}$C NMR ($CDCl_3$, δ): **A** 171.9 (C12), 143.4 (Ts), 138.3 (Ts), 134.8 (C8), 129.7 (Ts), 129.7 (C09), 128.6 (C10), 128.0 (Ts), 127.2 (C11), 127.1 (C3), 125.0 (C4), 57.5 (C7), 55.3 (C2), 52.3 (C13), 38.8 (C6), 22.7 (C5), 21.6 (Ts). **B** 172.2 (C12), 143.1 (Ts), 137.4 (Ts), 135.6 (C8), 129.4 (C09), 129.0 (C10), 128.8 (Ts), 128.0 (Ts), 127.5 (C11), 127.3 (C4), 126.1 (C3), 56.7 (C7), 54.9 (C2), 52.4 (C13), 38.4 (C6), 22.9 (C5), 21.6 (Ts).

ESI-MS: obs'd (%), calc'd (%), ppm, (M+H)+: 386.1420 (100), 386.1421 (100), 0.2. (M+Na)+: 408.1242(100), 408.1240 (100), 0.5.

**Synthesis and characterization of (*N*-mesyl)-5-imidazole-2-(methyl-*α*-phenylacetate)-1,2,5,6-tetrahydropyridine (22)**

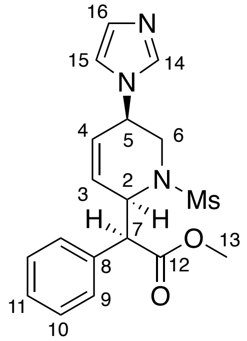


**16D** (62 mg, 0.071 mmol) was dissolved in 2 mL propionitrile in a test tube with a stir pea. In a separate test tube, DDQ (16 mg, 0.070 mmol) was dissolved in 2 mL propionitrile, and HOTf (32 mg, 0.211 mmol) was added. Both test tubes were chilled to -40 °C for 15 minutes. The DDQ/HOTf solution was added to the THP complex and allowed to stir for 1 minute. The solution was removed from the glove box and diluted with DCM (50 mL), and extracted with $Na_2CO_3$ (2 x 20 mL). The aqueous solution was back extracted with DCM (3 x 6 mL). The organic solution was dried with $Na_2SO_4$, filtered on a 15mL medium porosity frit, and rinsed with 5 mL diethyl ether. The solvent was removed, dissolved in minimal DCM, and added to stirring hexanes. The precipitate was filtered off using a 15 mL medium porosity frit and washed with 15 mL diethyl ether. The filtrate was reduced, and **22** (8 mg, 30% yield) was isolated.

$^1$H NMR ($(CD_3)_2CO$, δ, 25 °C): 7.70 (bs, 1H, H14), 7.48 (d, *J* = 7.5 Hz, 2H, H9), 7.39 (t, *J* = 7.2 Hz, 2H, H10), 7.34 (t, *J* = 7.6 Hz, 1H, H11), 7.20 (bs, 1H, H15), 6.98 (bs, 1H, H16), 6.2 (ddd, *J* = 2.5, 3.9, 6.4 Hz, 1H, H3), 6.01 (dd, *J* = 1.5, 10.5 Hz, 1H, H4), 5.1 (dd, *J* = 1.4, 10.4 Hz, 1H, H2), 5.05 (m, *J* = 2.1, 5.6, 8.1 Hz, 1H, H5), 4.17 (d, *J* = 10.6 Hz, 1H, H7), 3.88 (dd, *J* = 6.1, 14.2 Hz, 1H, H6), 3.72 (s, 3H, H13), 3.37 (dd, *J* = 10.5, 14.3 Hz, 1H, H6), 2.08 (s, 3H, Ms).

$^{13}$C NMR ($(CD_3)_2CO$, δ, 25 °C): 172.9 (C12), 137.7 (C8), 137.4 (C14), 130.9 (C3), 130.0 (C16), 129.9 (2C, C9), 129.7 (2C, C10), 129.3 (C4), 129.0 (C11), 118.5 (C15), 56.0 (C2), 55.4 (C7), 52.8 (C13), 51.3 (C5), 45.2 (C6), 39.8 (Ms).

SC-XRD data on S73.

**Synthesis and characterization of (*N*-mesyl)-5-pentyl sulfide-2-(methyl-*α*-phenylacetate)-1,2,5,6-tetrahydropyridine (23)**

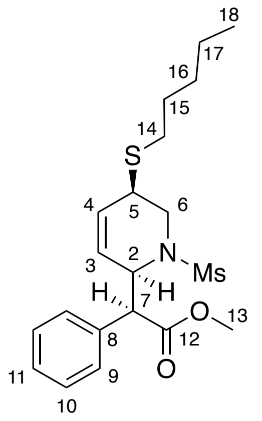


**19D** (88 mg, 0.096 mmol) was dissolved in 2 mL propionitrile in a test tube with a stir pea. In a separate test tube, DDQ (24 mg, 0.106 mmol) was dissolved in 2 mL propionitrile. The DDQ solution was added to the THP complex and allowed to stir for 10 minutes. The solution was removed from the glove box and packed onto a basic alumina column, **23** was collected after reducing the eluent from a 35% EtOAc/Hexanes solution (8 mg, 65% yield).

$^1$H NMR (($CDCl_3$), δ, 25 °C): 7.39 (d, *J* = 7.6 Hz, 2H, H9), 7.35 (t, *J* = 7.3 Hz, 2H, H10), 7.30 (t, *J* = 7.5 Hz, 1H, H11), 5.96 (ddd, *J* = 2.3, 4.1, 10.4 Hz, 1H, H3), 5.90 (ddd, *J* = 1.7, 3.3, 10.4 Hz, 1H, H4), 4.98 (dd, *J* = 1.9, 10.8 Hz, 1H, H2), 3.79 (dd, *J* = 6.0, 14.2 Hz, 1H, H6), 3.70 (s, 3H, H13), 3.69 (d, *J* = 10.8 Hz, 1H, H7), 3.54 (m, 1H, H5), 2.94 (dd, J = 11.3, 14.0 Hz, 1H, H6), 2.58 (dt, *J* = 2.9, 7.1 Hz, 2H, H14), 1.91 (s, 3H, Ms), 1.60 (q, *J* = 7.4, 14.8 Hz, 2H, H15), 1.36 (m, 2H, H16), 1.33 (m, 2H, H17), 0.90 (t, *J* = 7.51 Hz, 3H, H18).

$^{13}$C NMR (($CDCl_3$), δ, 25 °C): 171.77 (C14), 136.39 (C13), 130.53 (C4), 129.06 (C11), 128.86 (C10), 128.45 (C12), 128.12 (C3), 55.76 (C8), 55.44 (C2), 52.56 (C9), 45.07 (C6), 39.85 (C7), 38.70 (C5), 31.15 (C17), 30.66 (C15), 29.86 (C16), 22.38 (C18), 14.08 (C19).

HRMS (APCI) m/z: $[M]^+$ Calcd for $C_{20}H_{30}NO_4S_2^+$ 412.1611; Found 412.1614.

**Synthesis and characterization of (*N*-mesyl)-5-malononitrile-2-(methyl-*α*-phenylacetate)-1,2,5,6-tetrahydropyridine (24)**

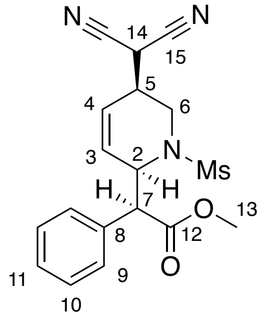


**12D** (134 mg, 0.153 mmol) was dissolved in 5 mL of acetone in a vial with a stir pea. Ammonium Cerium(IV) Nitrate (CAN) (345 mg, 0.629 mmol) was added to the solution and allowed to react for 4 hours. The solution was diluted with DCM (50 mL) and extracted with $H_2O$ (3 x 300 mL). The aqueous solution was back extracted with DCM (3 x 6 mL). The organic solution was dried with $Na_2SO_4$, filtered, and rinsed with DCM. The solvent was removed and packed onto a basic alumina column rinsed with 40% EtOAc/Hexanes solution. **24** (30 mg, 53% yield) was isolated.

$^1$H NMR (($CDCl_3$), δ, 25 °C): 7.46 (d, *J* = 8.03 Hz, 2H, H9), 7.40 (t, *J* = 7.42 Hz, 2H, H10), 7.35 (t, *J* = 7.42 Hz, 1H, H11), 6.29 (ddd, *J* = 2.34, 4.16, 6.38 Hz, 1H, H3), 5.98 (dd, *J* = 3.28, 10.41 Hz, 1H, H4), 5.03 (d, *J* = 10.82 Hz, 1H, H2), 4.79 (d, *J* = 5.14 Hz, 1H, H14), 3.93 (dd, *J* = 4.93, 13.08 Hz, 1H, H6), 3.87 (d, *J* = 10.72 Hz, 1H, H7), 3.70 (s, 3H, H13), 3.26 (bs, 1H, H5), 3.22 (dd, *J* = 11.00, 13.40 Hz, 1H, H6), 2.12 (s, 3H, Ms).

$^{13}$C NMR (($CDCl_3$), δ, 25 °C): 172.6 (C12), 137.3 (C8), 132.7 (C3), 129.8 (4C, C9/C10), 129.1 (C11), 126.3 (C4), 113.0 (2C, C15), 56.4 (C7), 56.2 (C2), 52.8 (C13), 41.9 (C6), 40.0 (Ms), 35.8 (C5), 26.2 (C14).

HRMS (APCI) m/z: $[M]^+$ Calcd for $C_{18}H_{20}N_3O_4S^+$ 374.1169; Found 374.1166.

**Synthesis and characterization of (*N*-mesyl)-5-dimethyl malonate-2-(methyl-*α*-phenylacetate)-1,2,5,6-tetrahydropyridine (25)**

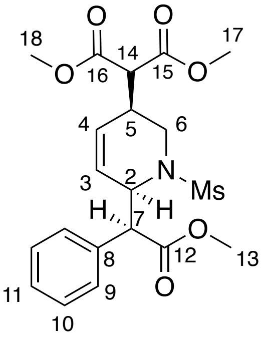


**10D** (62 mg, 0.066 mmol) was dissolved in 2ml of acetone in a vial with a stir pea. DDQ (38 mg, 0.167 mmol) was added to the solution and allowed to react for 3 hours. The solution was diluted with DCM (50ml) and extracted with $H_2O$ (3 x 100 mL). The aqueous solution was back extracted with DCM (3 x 6 mL). The organic solution was dried with $Na_2SO_4$, filtered, and rinsed with DCM. The solvent was removed, redissolved in minimal DCM, and precipitated in hexanes. The precipitate was rinsed with ether, and the filtrate was reduced. **25** (20 mg, 69% yield) was isolated.

$^1$H NMR (($CDCl_3$), δ, 25 °C): 7.42 (d, *J* = 7.59 Hz, 2H, H9), 7.37 (t, *J* = 7.59 Hz, 2H, H10), 7.32 (t, *J* = 7.26 Hz, 1H, H11), 5.98 (ddd, *J* = 2.64, 4.12, 10.54 Hz, 1H, H3), 5.83 (dd, *J* = 1.45, 10.47 Hz, 1H, H4), 4.93 (d, *J* = 10.83, 1H, H2), 3.87 (d, *J* = 11.09, 1H, H7), 3.74-3.75 (m, 7H, H6/17/18), 3.68 (s, 3H, H13), 3.57 (d, *J* = 7.51 Hz, 1H, H14), 3.21 (dd, *J* = 11.07, 14.11 Hz, 1H, H6), 3.13 (m, 1H, H5), 2.06 (s, 3H, Ms).

$^{13}$C NMR (($CDCl_3$), δ, 25 °C): 172.80 (C12), 168.97/168.94 (2C, C15/16), 137.58 (C8), 129.80 (2C, C9), 129.65 (2C, C10), 129.55 (C11), 129.13 (C4), 128.93 (C3), 56.40 (C7), 56.08 (C2), 54.13 (C14), 52.99/52.97 (2C, C17/18), 52.68 (C13), 41.94 (C6), 39.92 (Ms), 34.63 (C5).

HRMS (APCI) m/z: $[M]^+$ Calcd for $C_{20}H_{26}NO_8S^+$ 440.1374; Found 440.1380.

**Synthesis and characterization of *erythro*-(*N*-mesyl)-methylphenidate (26)**

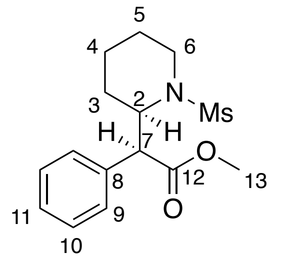

**21** (21 mg, 0.068 mmol) was dissolved in ethyl acetate (3 mL) and added to a 4-dram vial. This solution was then circulated through a ThalesNano H-Cube flow hydrogenator for 5 hours. The temperature was set to 50 °C, the $H_2$ pressure to 25 bars, and the catalyst cartridge used contained 5%Pd on carbon. After circulation, the solution was collected and reduced to dryness (17 mg 78% yield).

$^{1}$H NMR (($CD_3$)$_2$CO), δ, 25 °C): 7.52 (d, *J* = 7.2, 2H, H9), 7.36 (tt, *J* = 1.3, 7.2 Hz, 2H, H10), 7.31 (tt, *J* = 1.3, 7.3 Hz, 1H, H11), 4.75 (dd, *J* = 5.1, 11.8 Hz, 1H, H2), 4,32 (d, *J* = 11.8 Hz, 1H, H7), 3.66 (s, 3H, H13), 3.47 (dd, *J* = 3.3, 14.2 Hz, 1H, H6), 3.11 (ddd, *J* = 2.8, 13.0, 14.2 Hz, 1H, H6), 1.91 (s, 3H, Ms), 1.81-1.88 (m, 1H, H4), 1.73-1.78 (m, 1H, H3), 1.70-1.73 (m, 1H, H3), 1.67-1.70 (m, 1H, H4), 1.61-1.65 (m, 1H, H5), 1.48-1.55 (m, 1H, H5).

$^{13}$C NMR (($CD_3$)$_2$CO), δ, 25 °C): 173.2 (C12), 138.0 (C8), 129.7 (C9), 129.5 (C10), 128.7 (C11), 56.2 (C2), 52.5 (C7), 51.4 (C13), 41.3 (C6), 39.7 (Ms), 29.0 (C3), 26.0 (C5), 19.5 (C4).

SC-XRD data on S74.

**Synthesis and characterization of *erythro*-(*N*-mesyl)-methylphenidate (27)**

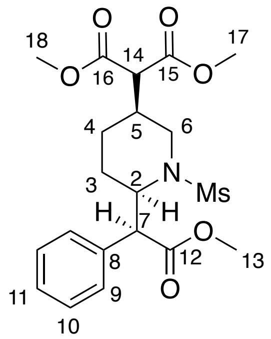


**25** (27mg, 0.061 mmol) was dissolved in ethyl acetate (3 mL) and added to a 4-dram vial. This solution was then circulated through a ThalesNano H-Cube flow hydrogenator for 5 hours. The temperature was set to 50 °C, the $H_2$ pressure to 25 bars, and the catalyst cartridge used contained 5%Pd on carbon. After circulation, the solution was collected and reduced to dryness (17 mg, 65% yield)

$^1$H NMR ($CD_2Cl_2$), δ, 25 °C): 7.42 (d, $J$ = 7.5 Hz, 2H, H9), 7.35 (t, $J$ = 7.5 Hz, 2H, H10), 7.30 (t, $J$ = 7.4 Hz, 1H, H11), 4.74 (dd, $J$ = 5.0, 11.5 Hz, 1H, H2), 4.05 (d, $J$ = 11.8 Hz, 1H, H7), 3.75 (s, 6H, H17/18), 3.66 (s, 3H, H13), 3.54 (dd, $J$ = 3.5, 13.7 Hz, 1H, H6), 3.29 (d, $J$ = 7.70 Hz, 1H, H14), 2.84 (dd, $J$ = 11.8, 13.7 Hz, 1H, H6), 2.25-2.31 (m, 1H, H5), 1.92 (s, 3H, Ms), 1.84-1.90 (m, 1H,H3), 1.73-1.79 (m, 2H, H3/4), 1.67 (dq, $J$ = 3.2, 12.8 Hz, 1H, H4).

$^{13}$C NMR ($CD_2Cl_2$), δ, 25 °C): 172.5 (C12), 168.5 (C15/16), 136.8 (C8), 129.2 (2C, C9/10), 129.1 (2C, C9/10), 128.6 (C11), 55.3 (C2), 55.1 (C14), 52.9 (2C, C17/18), 52.7 (C13), 51.2, (C7), 43.5 (C6), 39.9 (Ms), 36.2 (C5), 28.5 (C3), 23.4 (C4).

SC-XRD data on S75.

**NMR Spectroscopy**:

**Figure S1**: $^1$H NMR, 800 MHz, $CD_2Cl_2$, 25 °C, Compound **5**

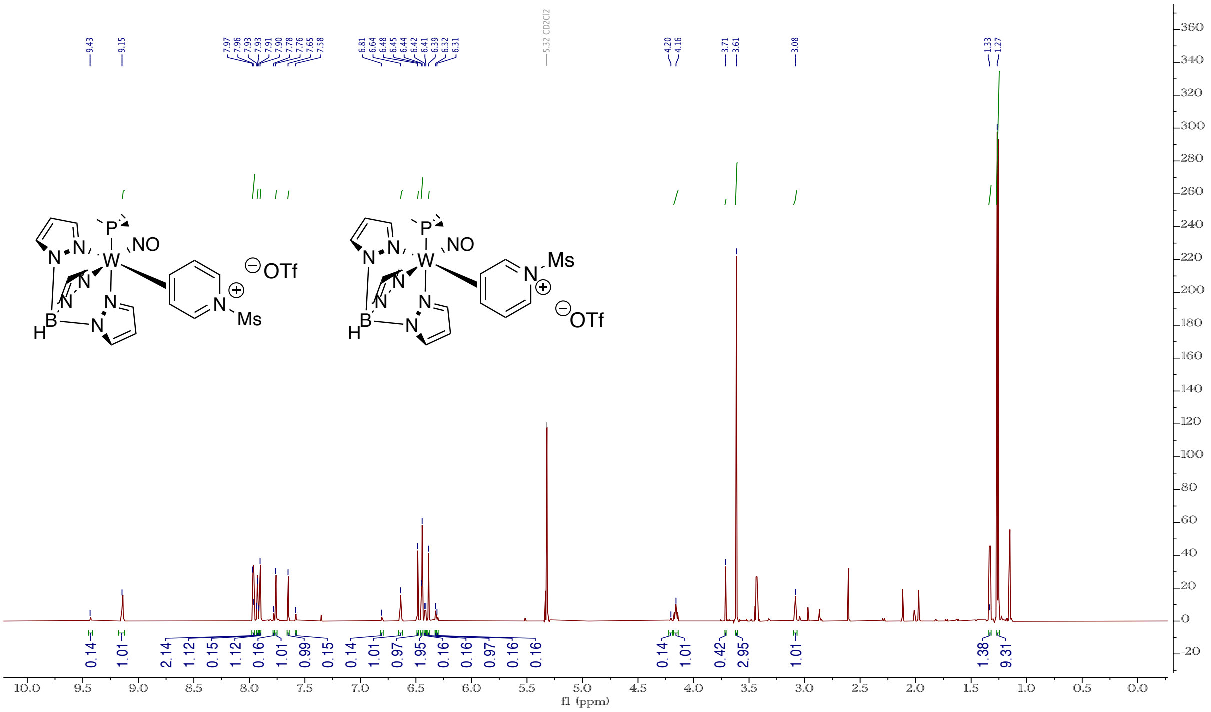

**Figure S2**: $^1$H NMR, 800 MHz, $(CD_3)_2CO$, 25 °C, Compound **5D**

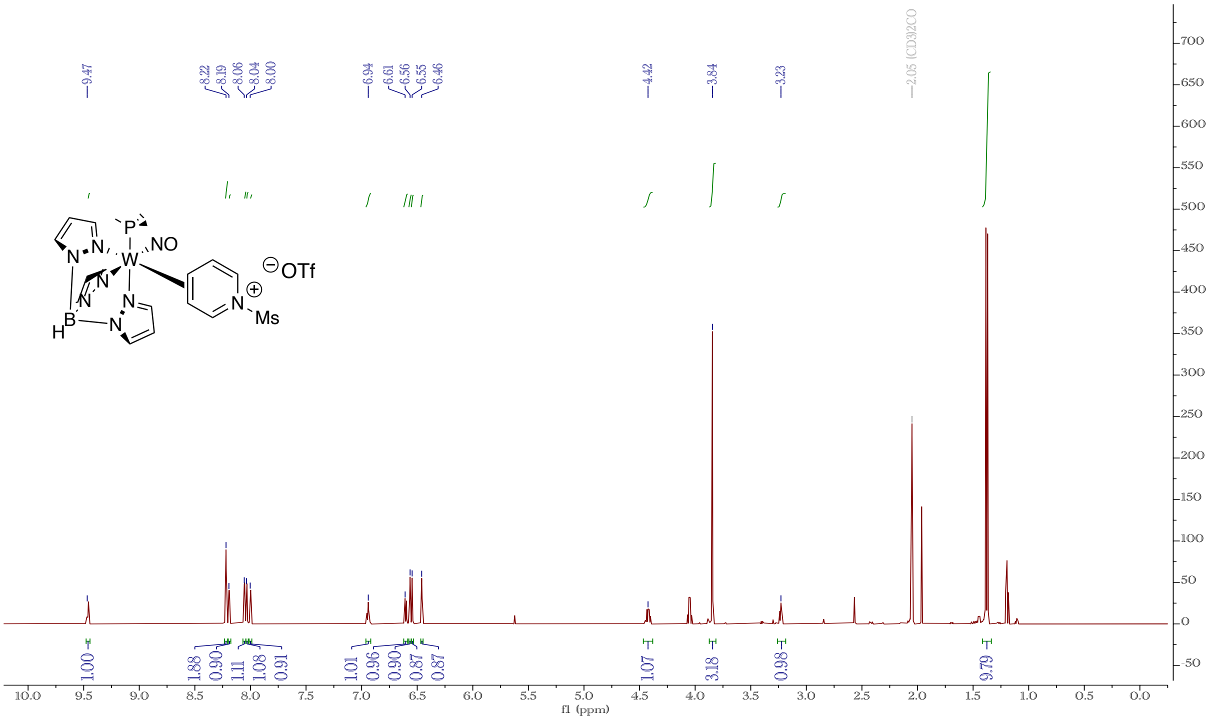

**Figure S3**: $^{13}C$ NMR, 200 MHz, $CD_2Cl_2$, 25 °C, Compound **5**

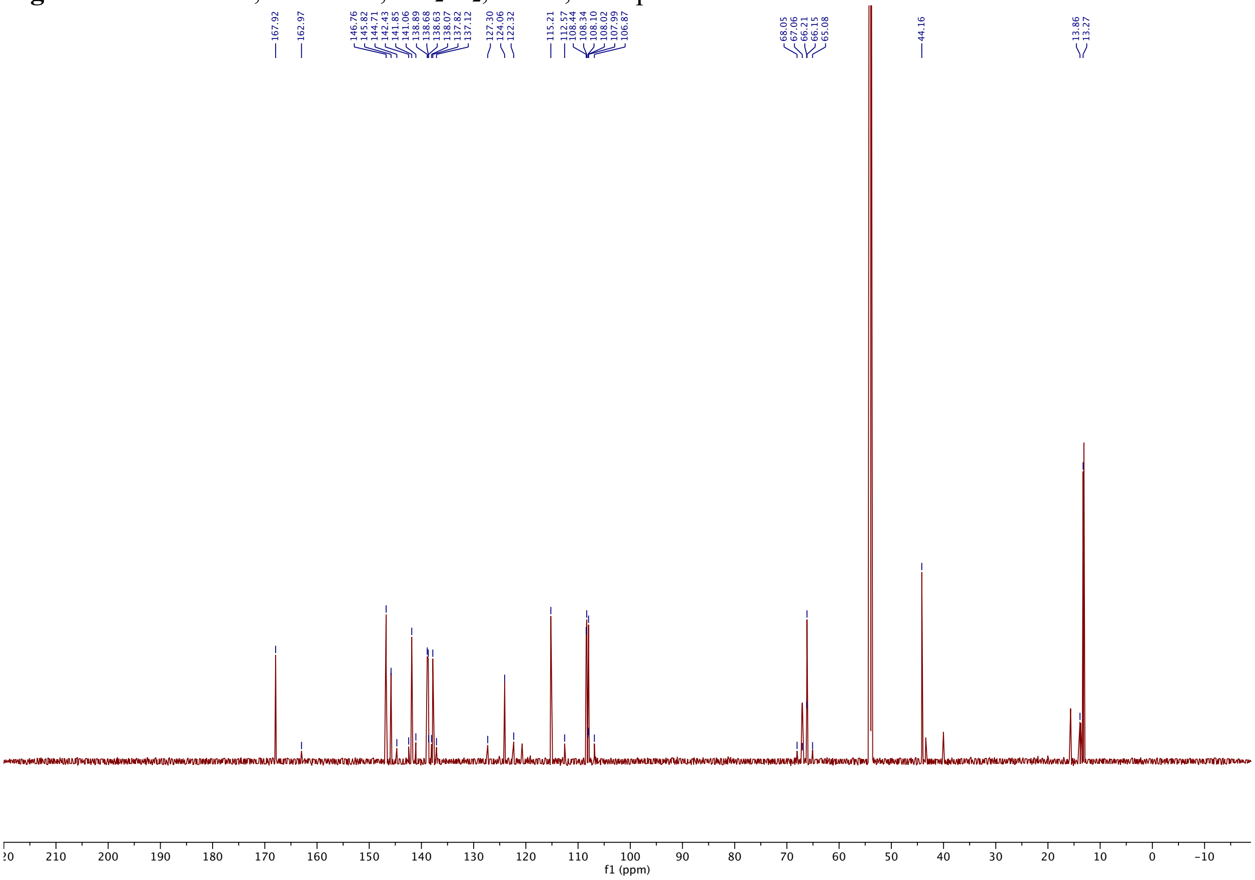

**Figure S4**: $^{1}H$ NMR, 800 MHz, $CD_2Cl_2$, 25 °C, Compound **6D**

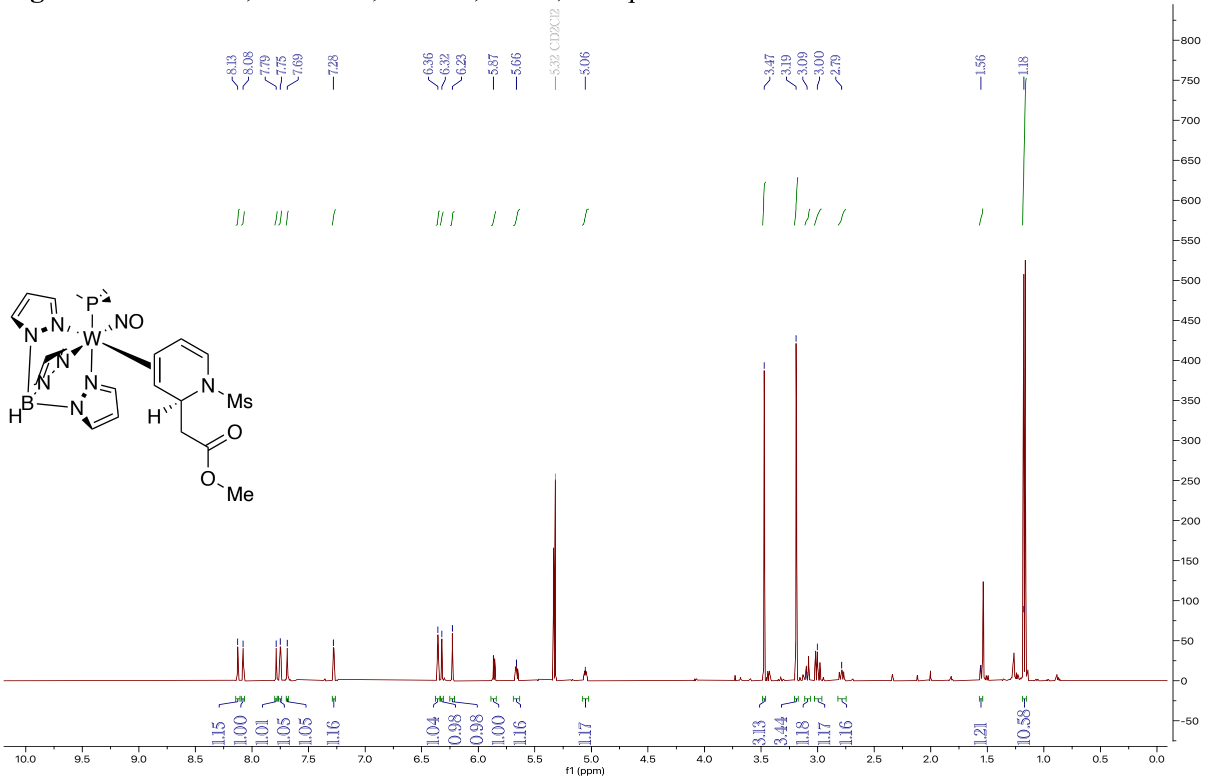


**Figure S5**: $^{13}C$ NMR, 200 MHz, $CD_2Cl_2$, 25 °C, Compound **6D**

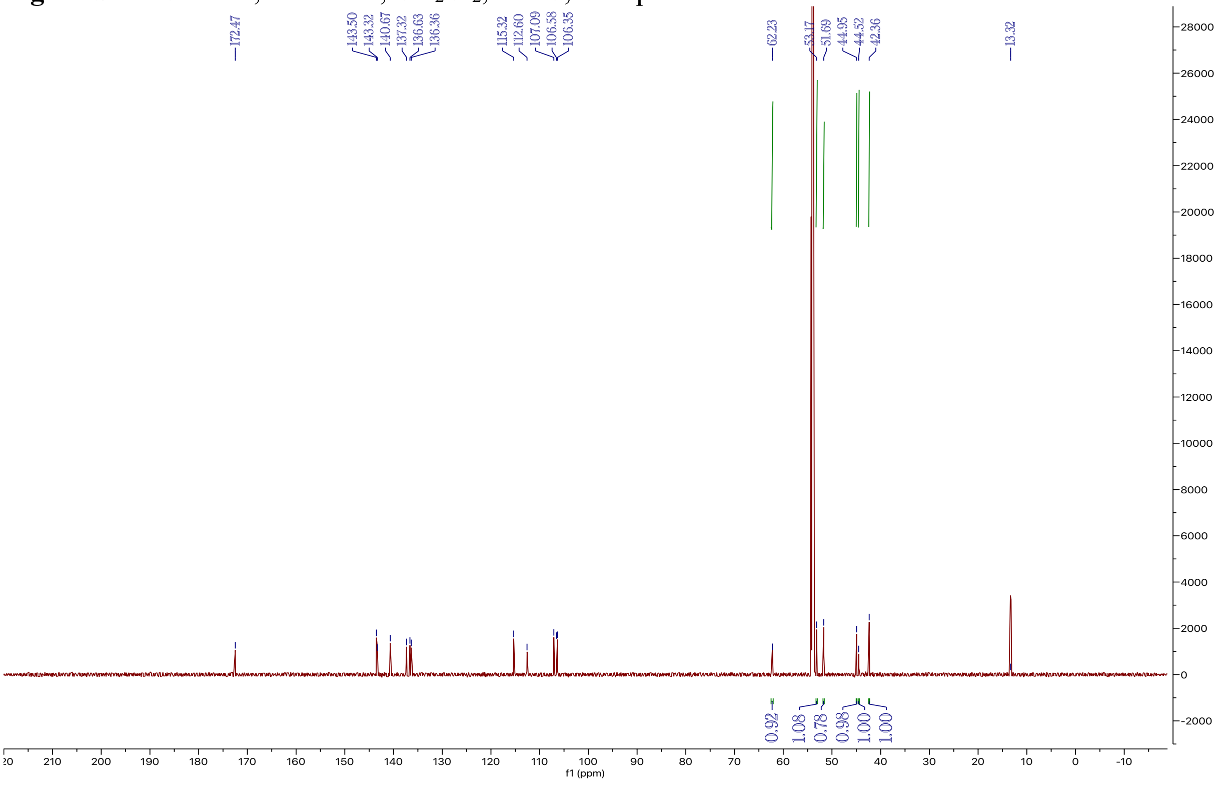

**Figure S6**: $^{1}H$ NMR, 800 MHz, $CD_2Cl_2$, 25 °C, Compound **7D**

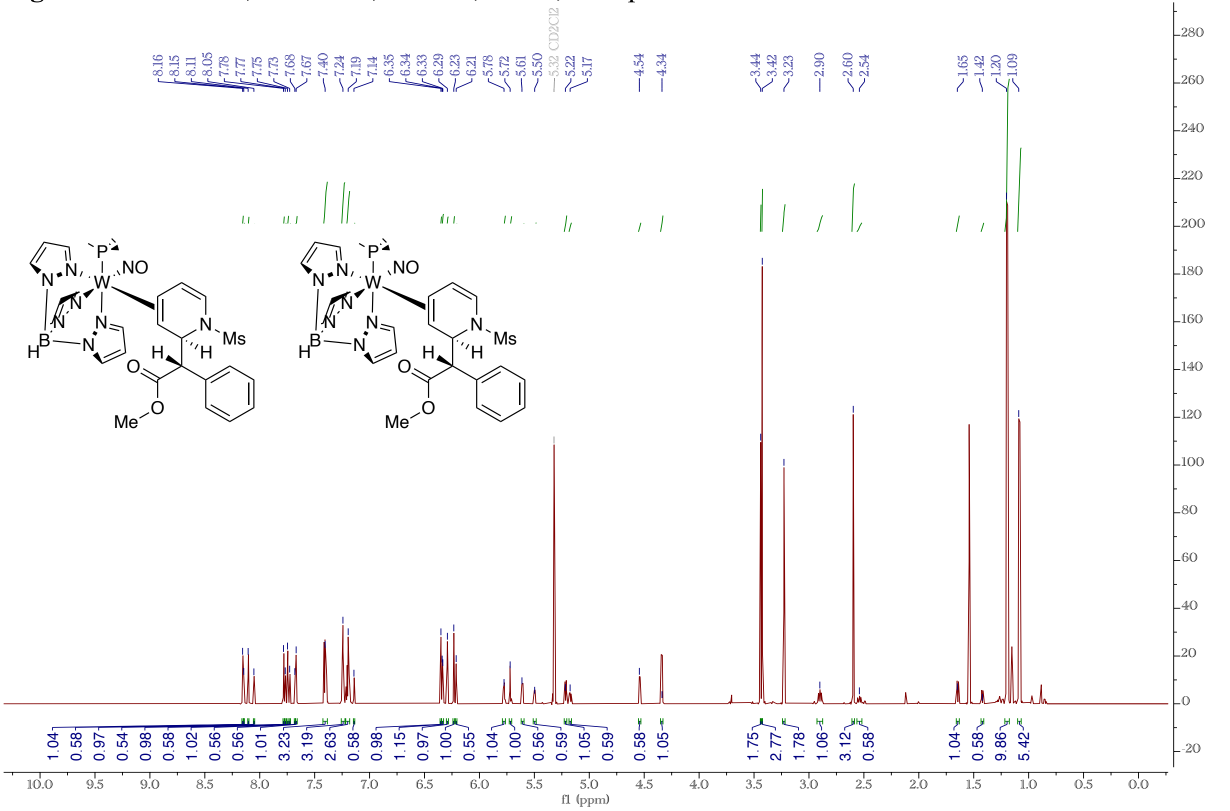

**Figure S7**: $^{13}C$ NMR, 200 MHz, $CD_2Cl_2$, 25 °C, Compound **7D**

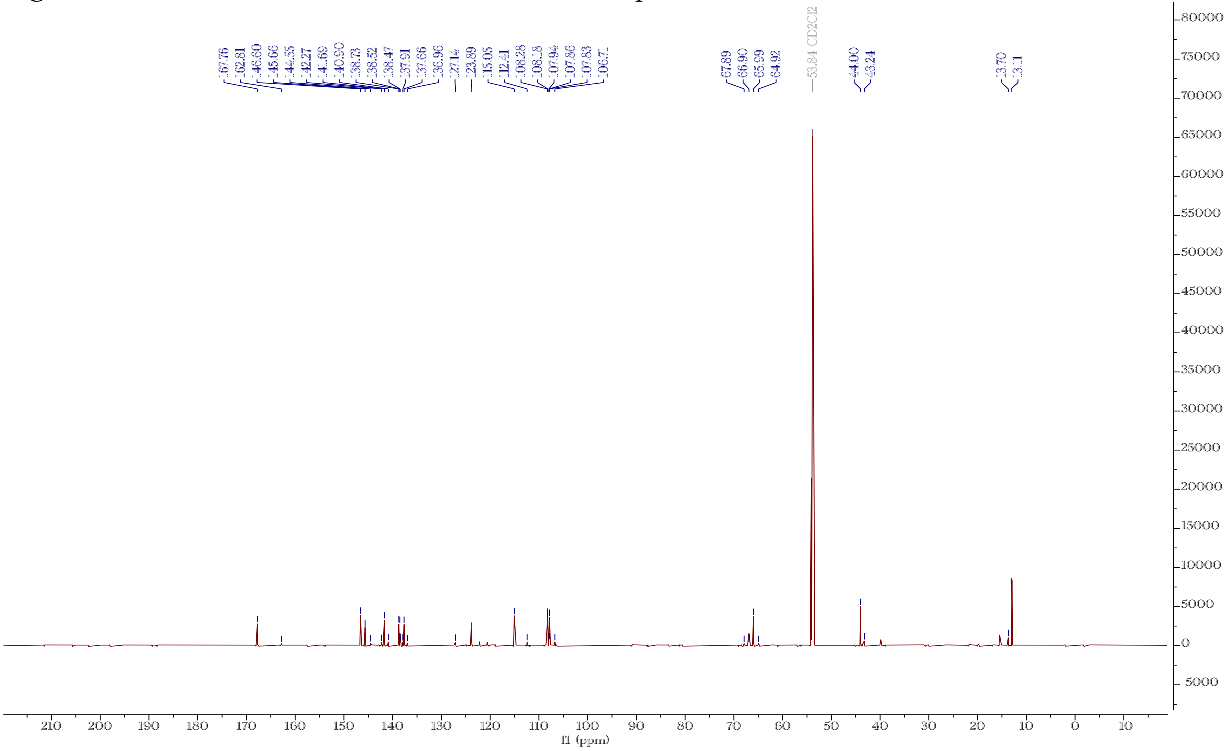

**Figure S8**: $^{1}H$ NMR, 800 MHz, $CD_2Cl_2$, 25 °C, Highly Enriched Compound ***erythro*-7D**

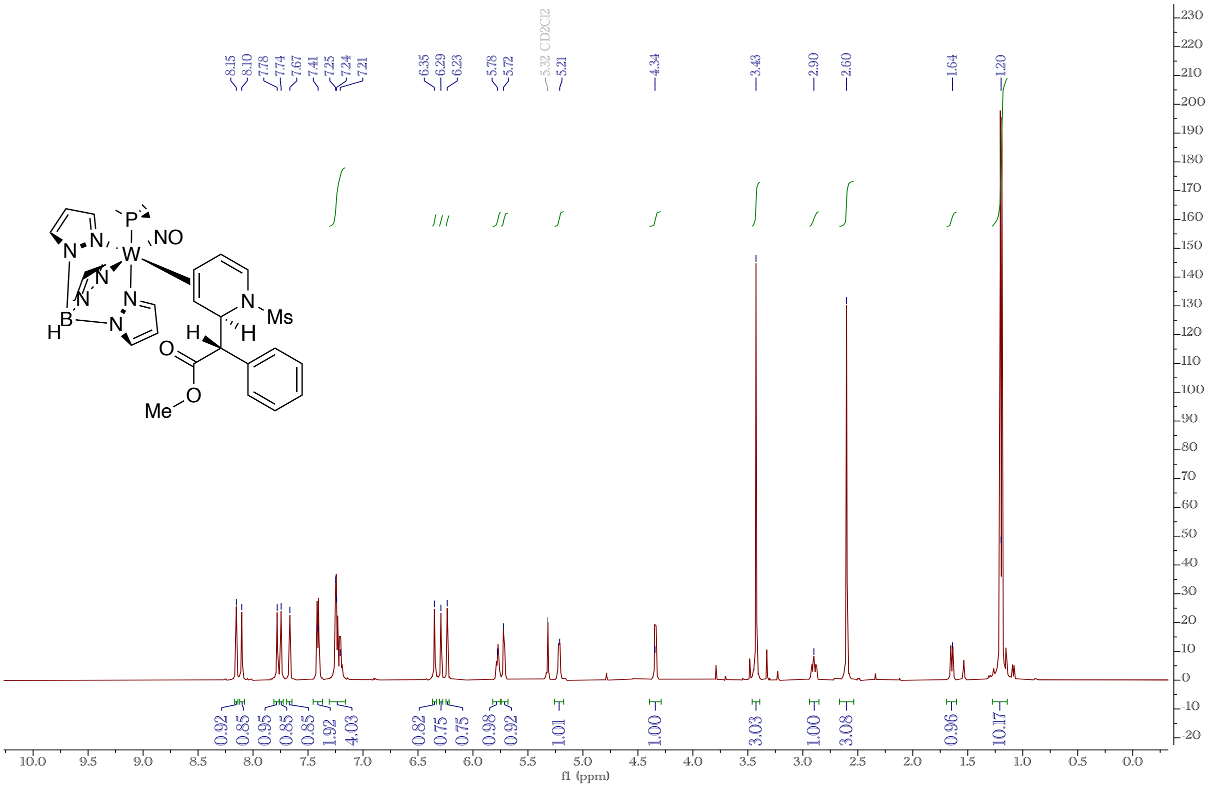


**Figure S9**: $^{13}C$ NMR, 200 MHz, $CD_2Cl_2$, 25 °C, Compound ***erythro*-7D**

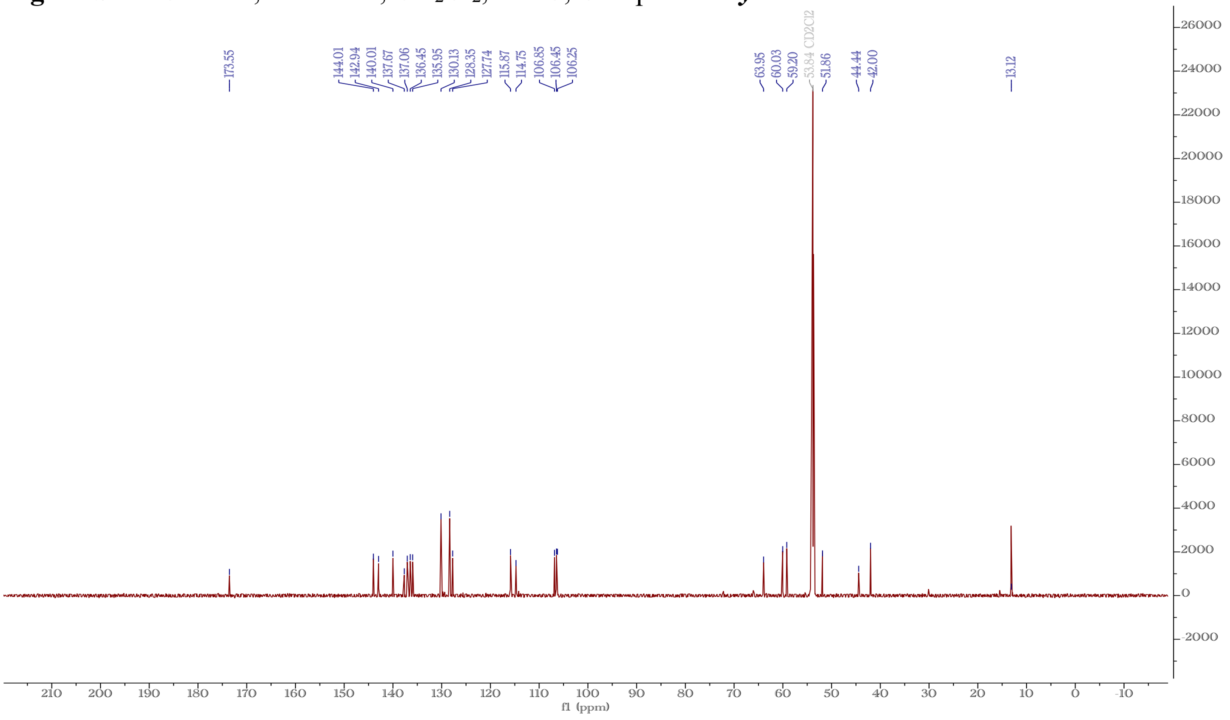

**Figure S10**: $^{1}$H NMR, 800 MHz, $CD_2Cl_2$, 25 °C, Compound **8**

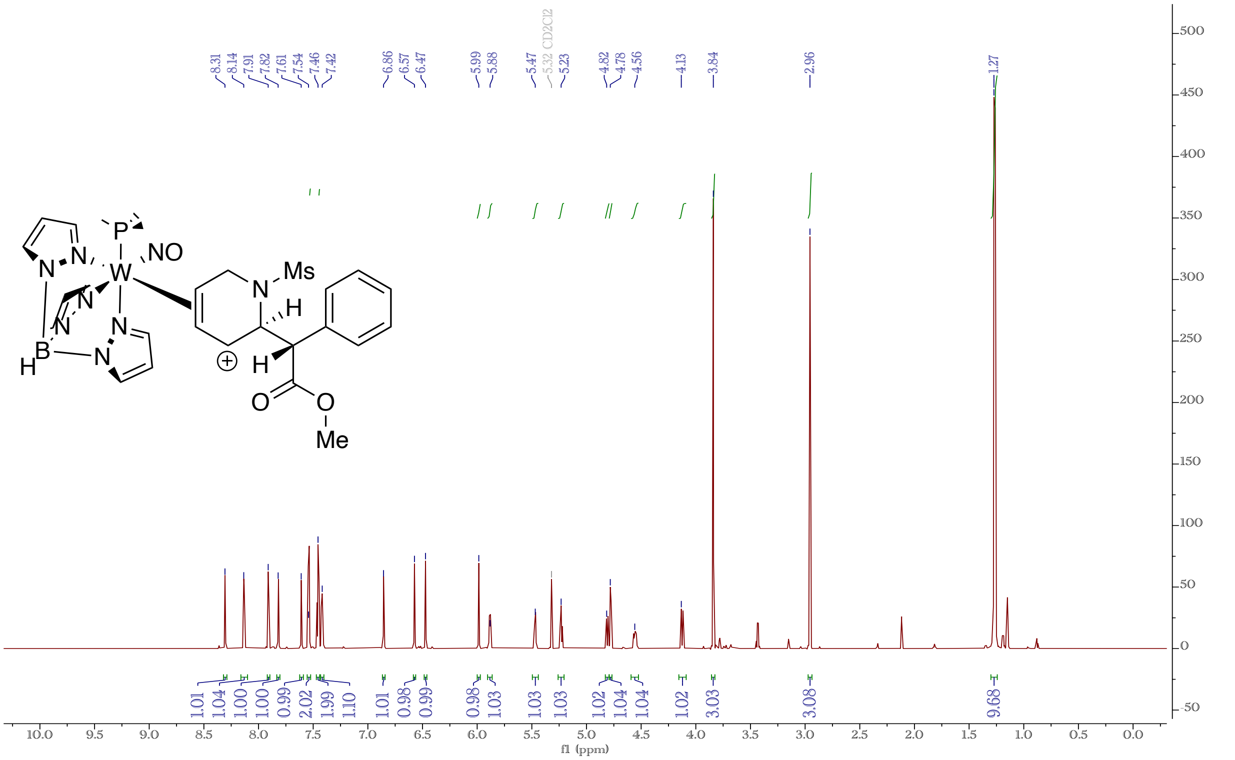


**Figure S11**: $^{13}$C NMR, 200 MHz, $CD_2Cl_2$, 25 °C, Compound **8**

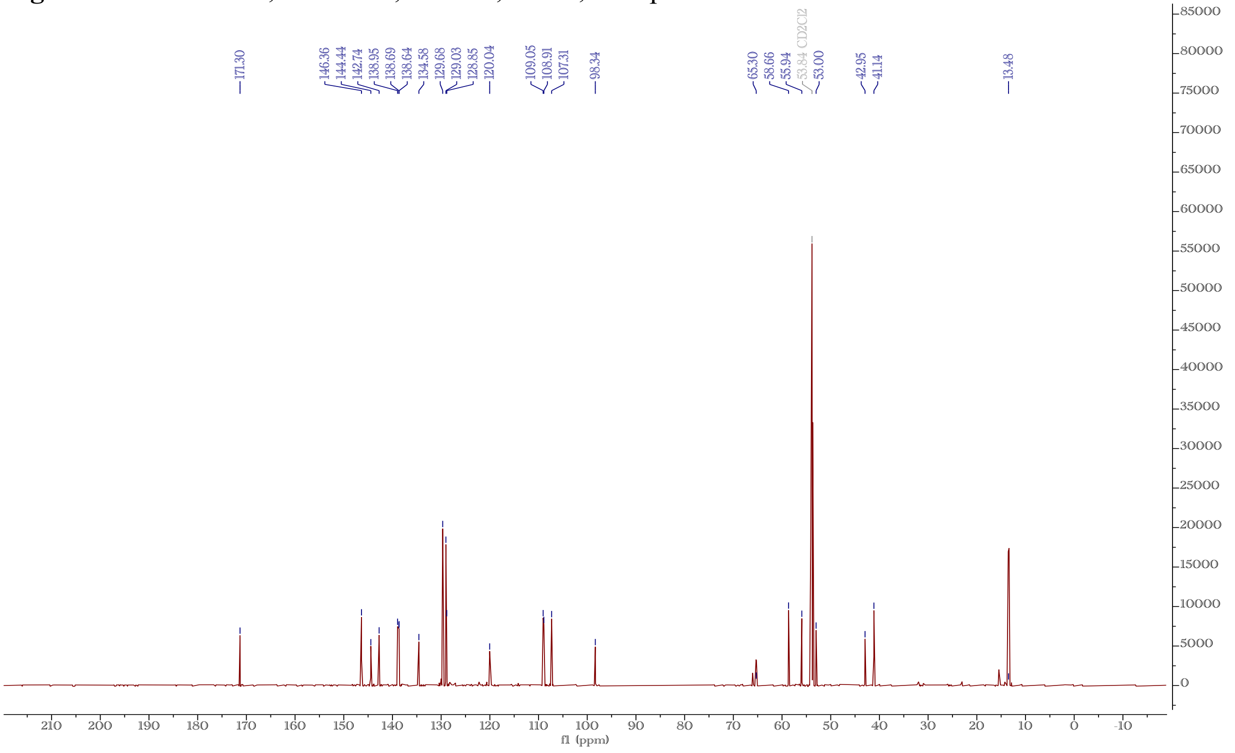

**Figure S12**: $^{1}$H NMR, 800 MHz, $(CD_3)_2CO$, 25 °C, Compound **9D**

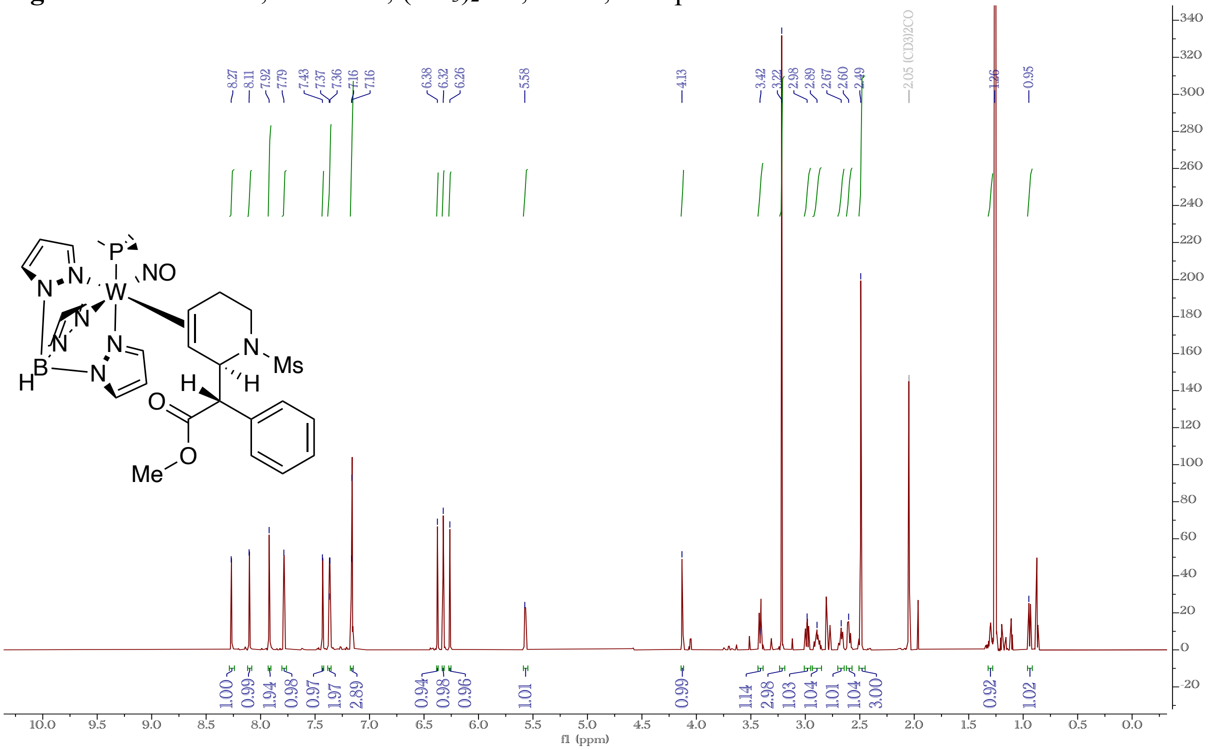


**Figure S13**: $^{13}$C NMR, 200 MHz, $(CD_3)_2CO$, 25 °C, Compound **9D**

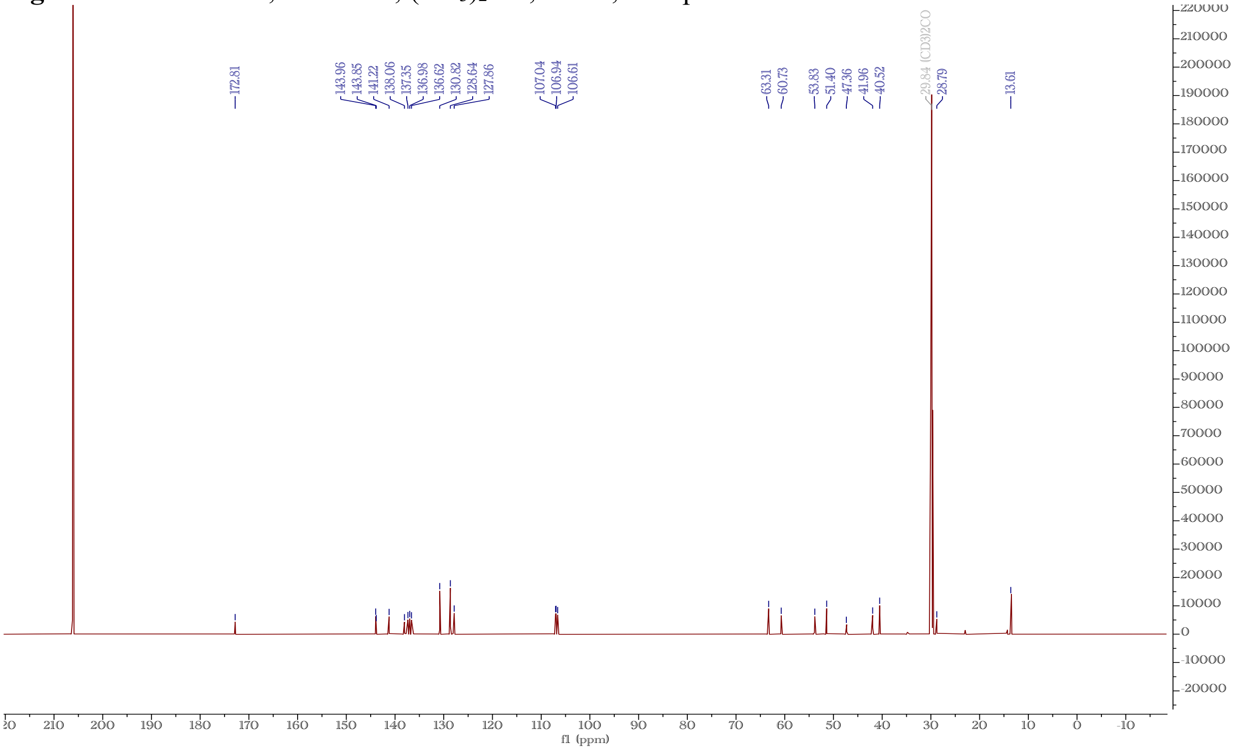

**Figure S14**: $^{1}$H NMR, 800 MHz, $CD_2Cl_2$, 25 °C, Compound **10D**

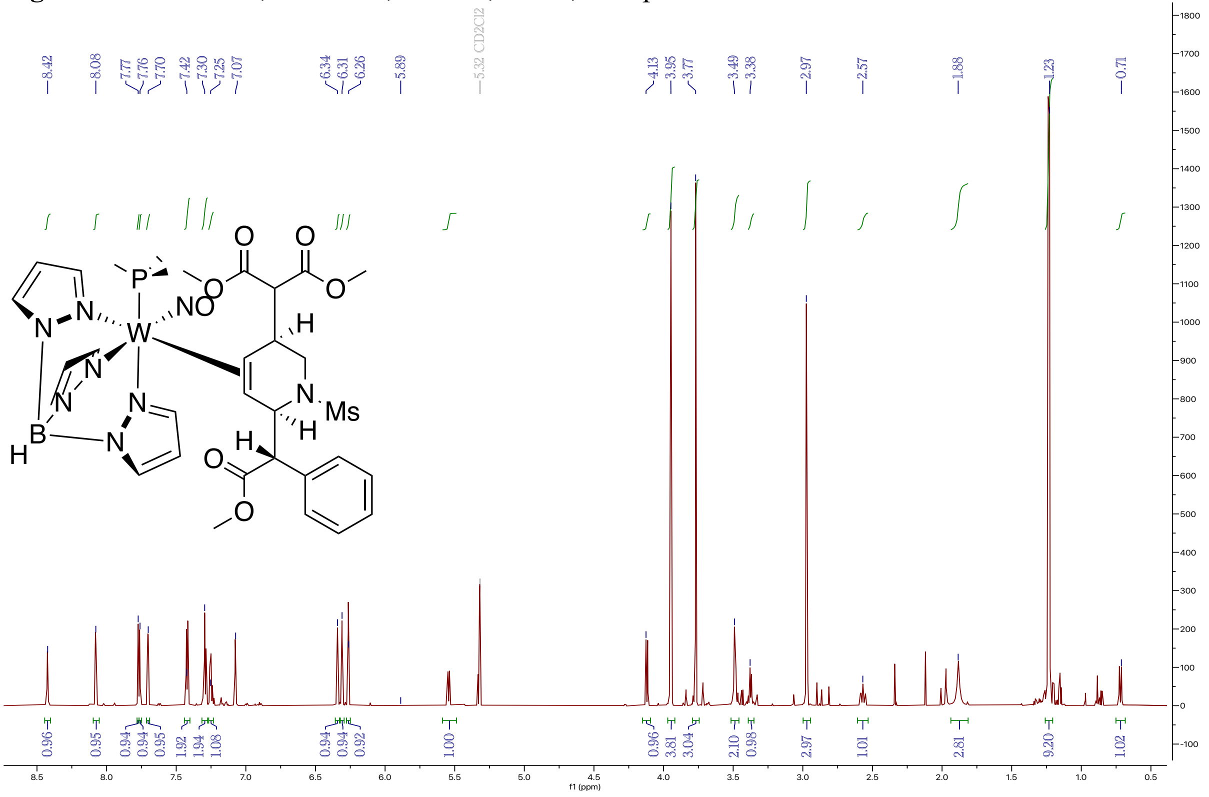


**Figure S15**: $^{13}$C NMR, 200 MHz, $CD_2Cl_2$, 25 °C, Compound **10D**

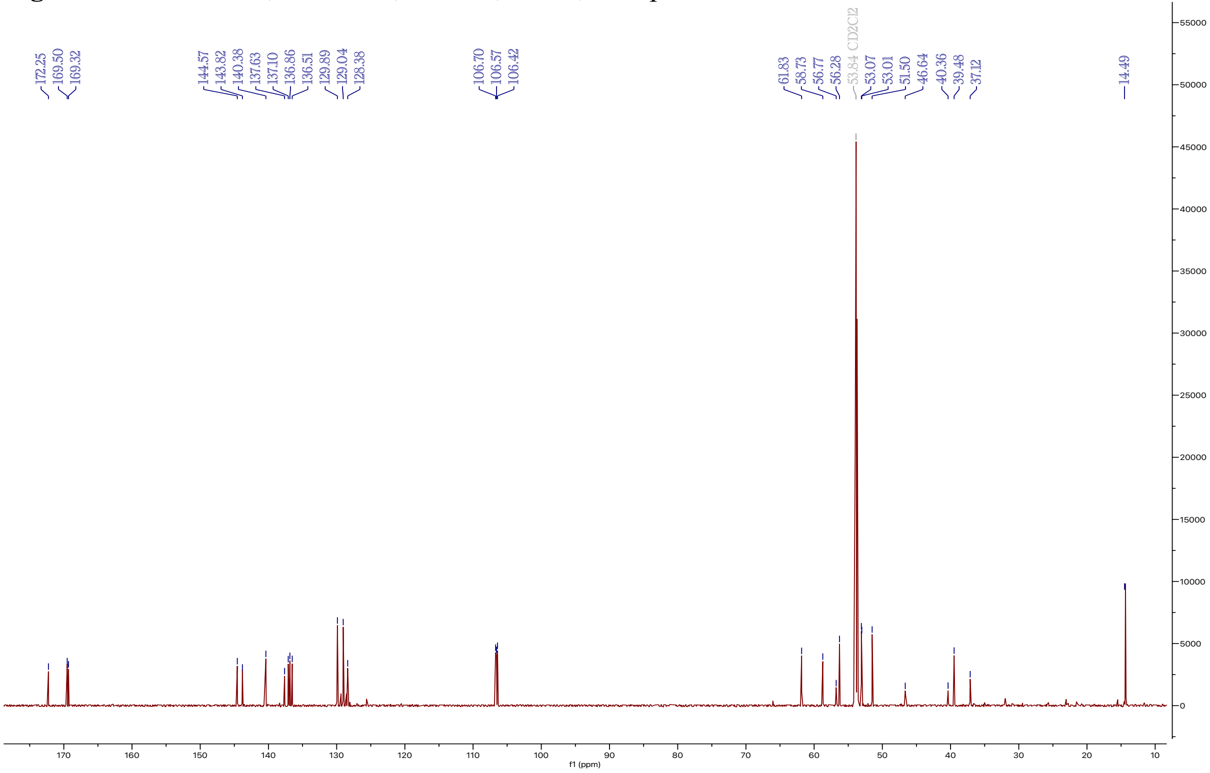

**Figure S16**: $^{1}H$ NMR, 800 MHz, $CD_2Cl_2$, 25 °C, Compound **11D**

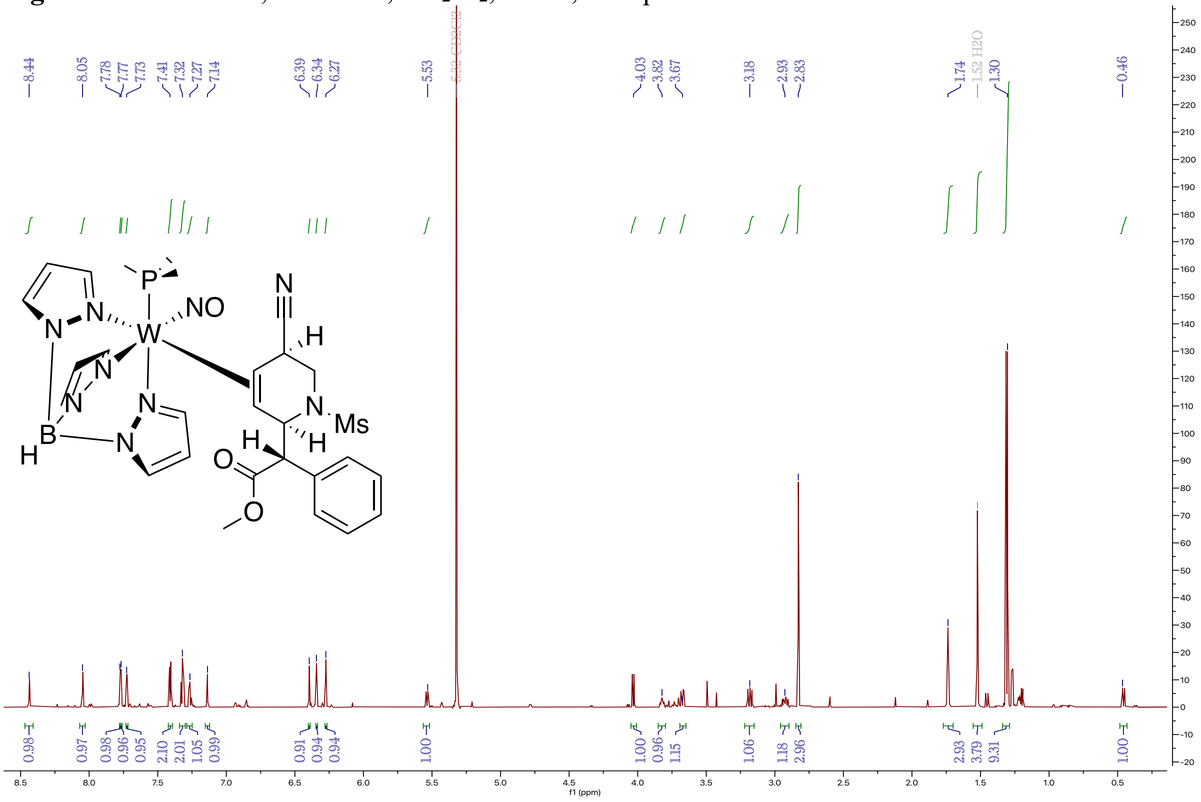


**Figure S17**: $^{13}C$ NMR, 200 MHz, $CD_2Cl_2$, 25 °C, Compound **11D**

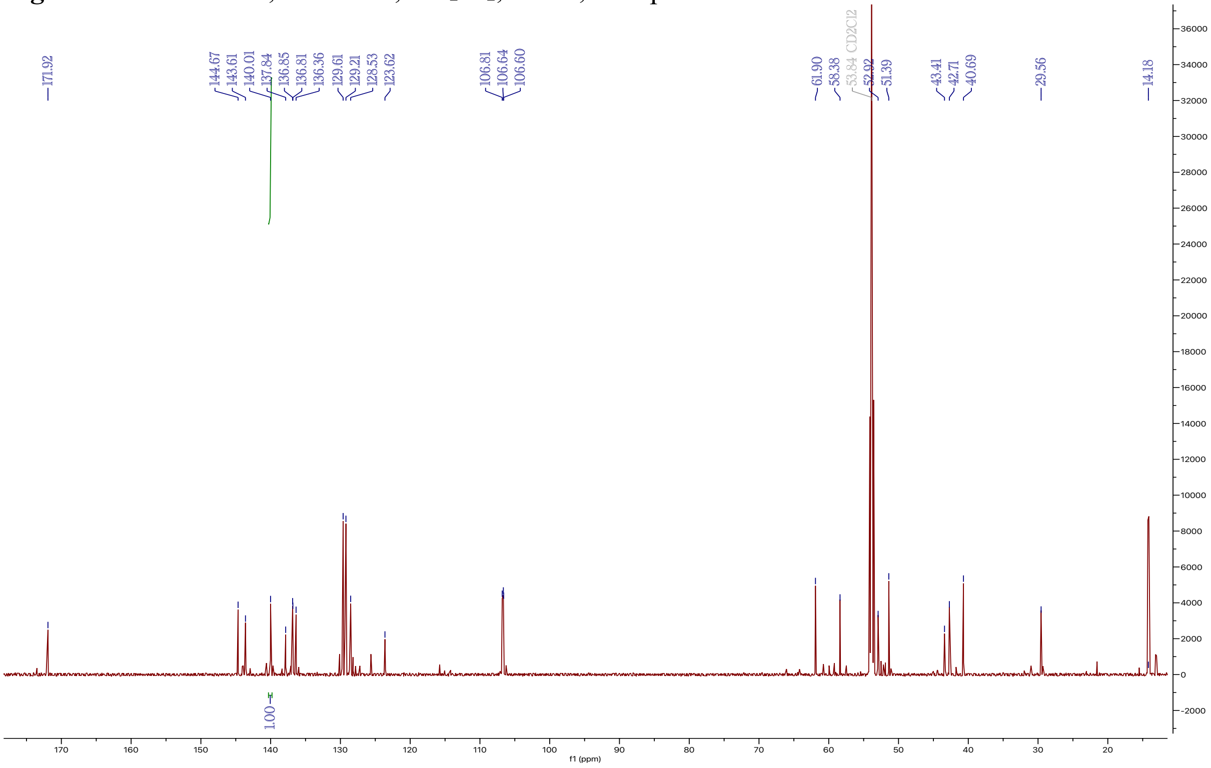

**Figure S18**: $^{1}H$ NMR, 800 MHz, $(CD_3)_2CO$, 25 °C, Compound **12D**

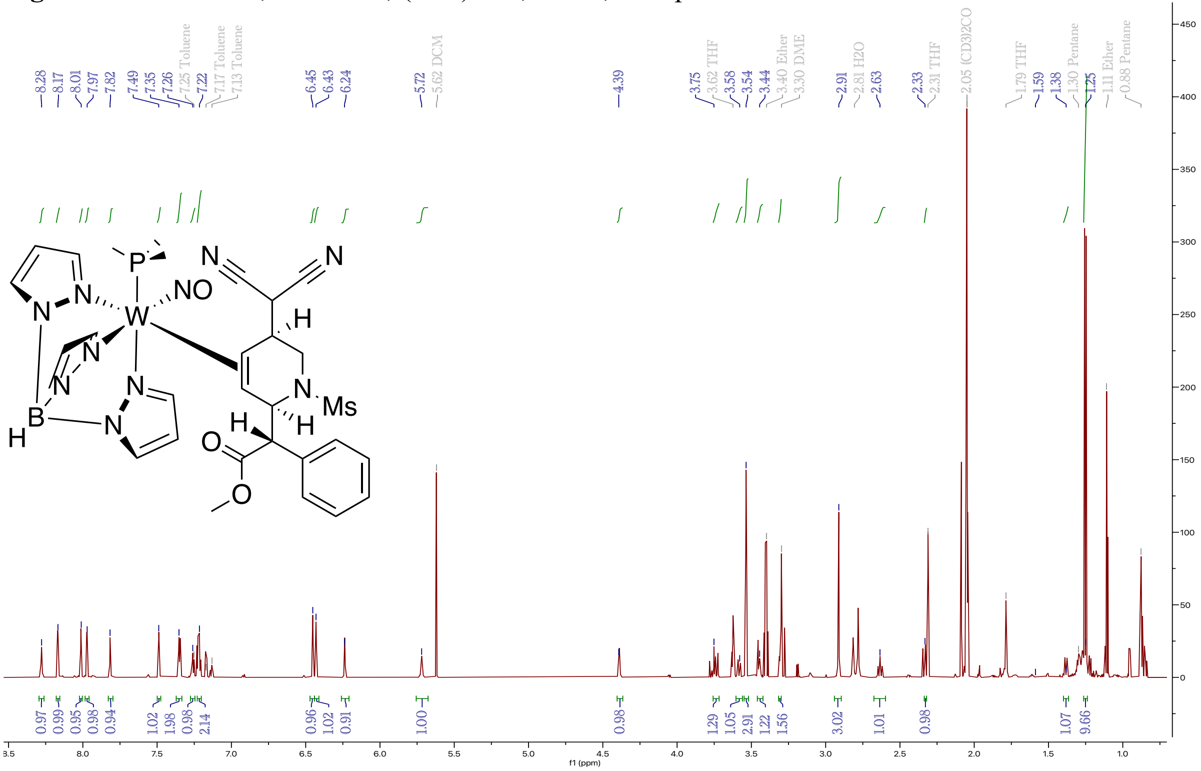

**Figure S19**: $^{13}C$ NMR, 200 MHz, $(CD_3)_2CO$, 25 °C, Compound **12D**

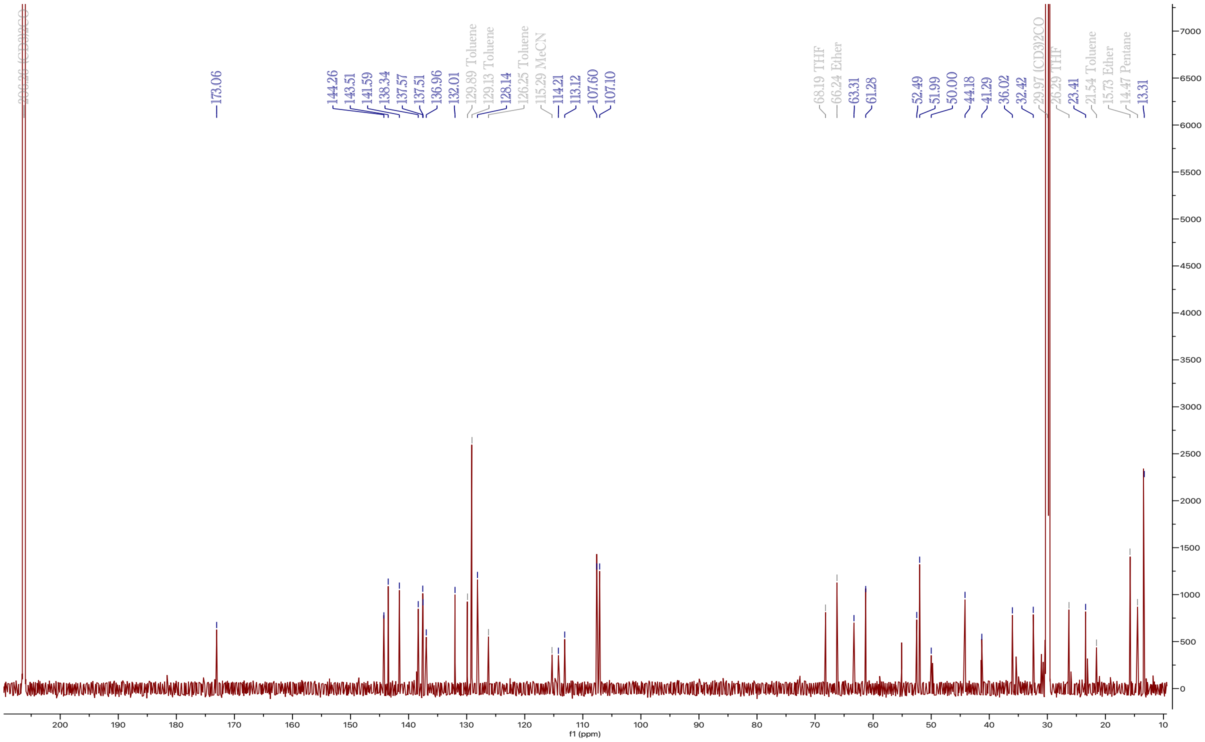

**Figure S20**: $^{1}$H NMR, 800 MHz, $CD_3CN$, 25 °C, Compound **13D**

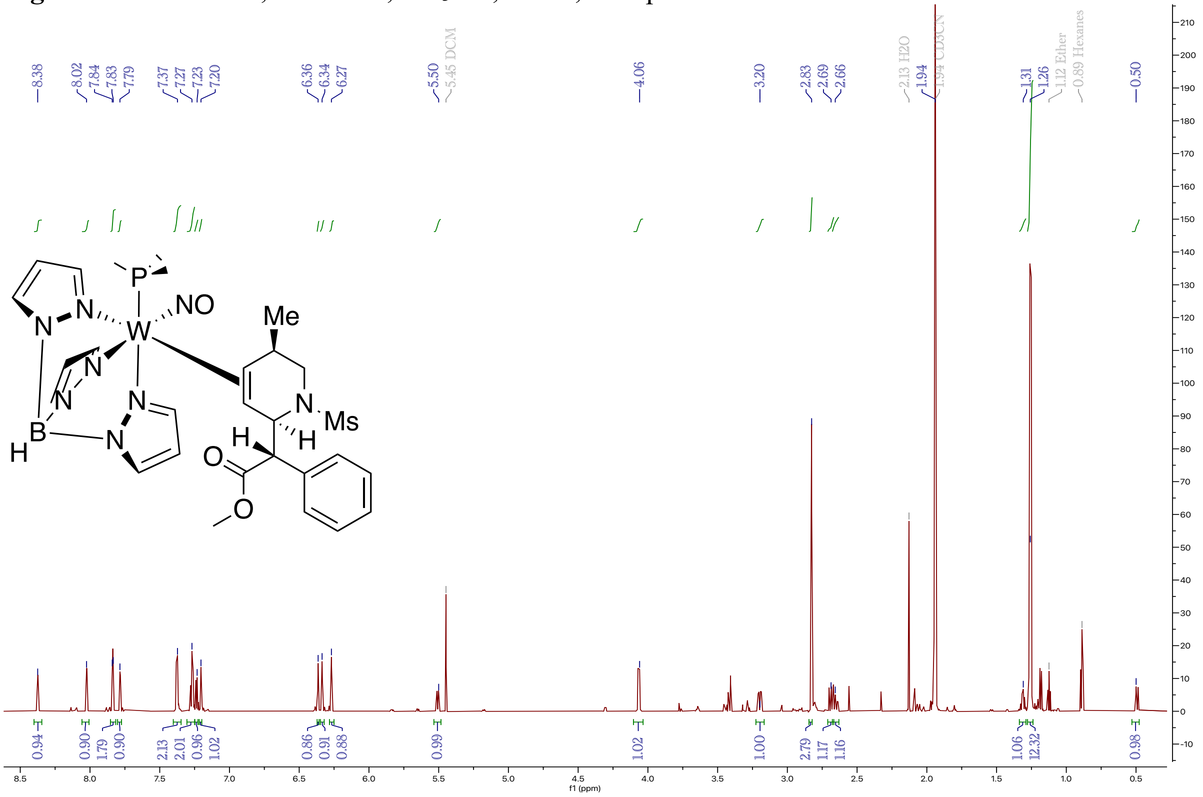


**Figure S21**: $^{13}$C NMR, 200 MHz, $CD_3CN$, 25 °C, Compound **13D**

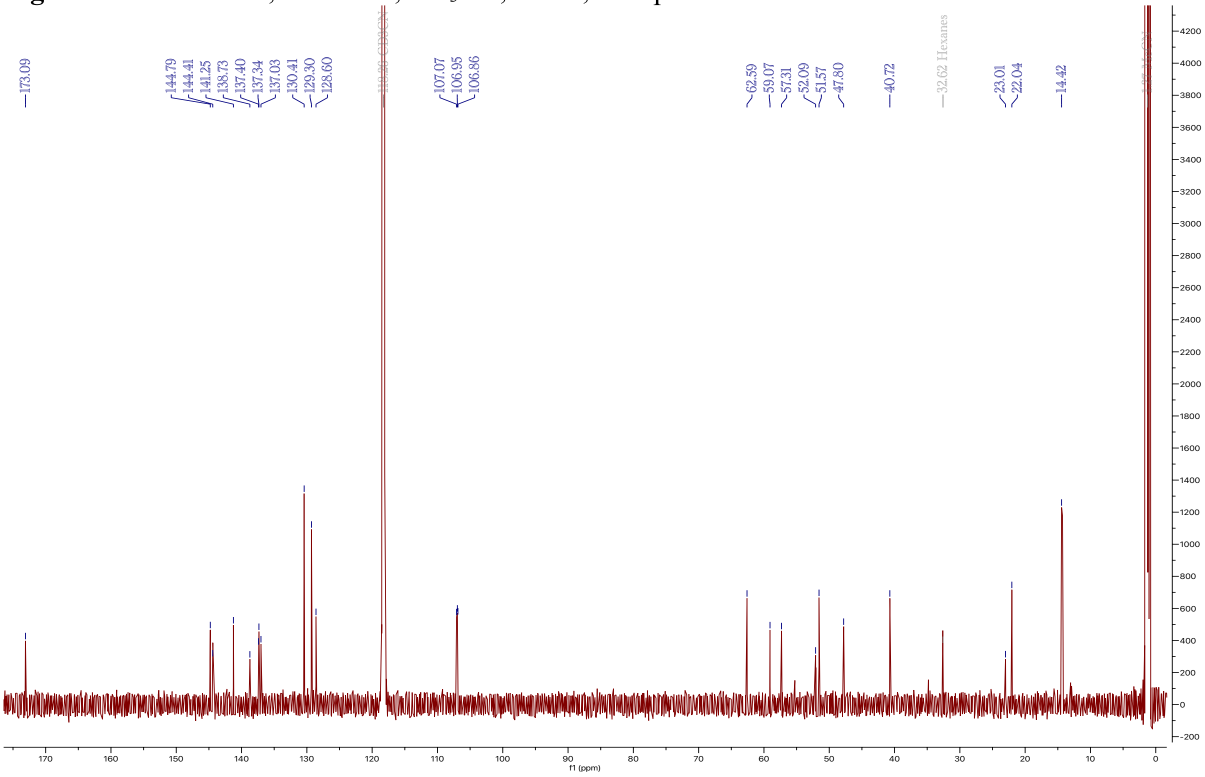

**Figure S22**: $^{1}H$ NMR, 800 MHz, $CD_3CN$, 25 °C, Compound **14D**

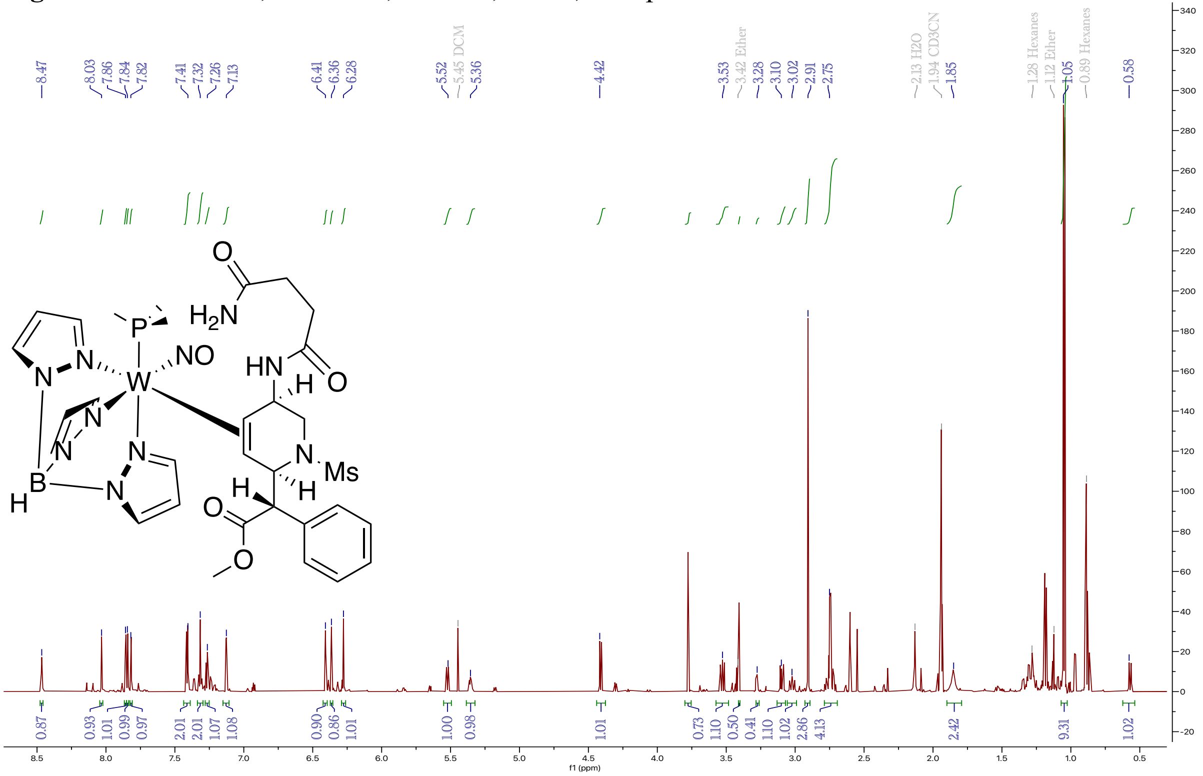


**Figure S23**: $^{13}C$ NMR, 200 MHz, $CD_3CN$, 25 °C, Compound **14D**

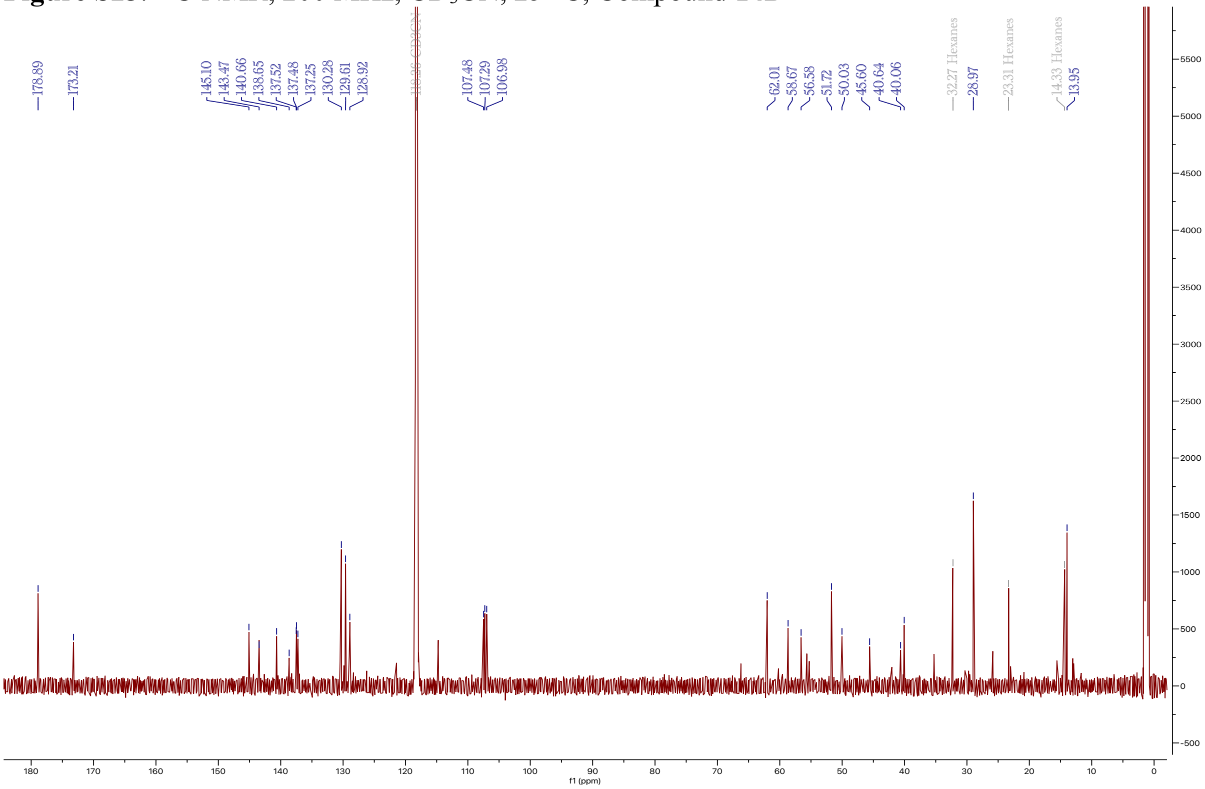

**Figure S24**: $^{1}H$ NMR, 800 MHz, $CD_2Cl_2$, 25 °C, Compound **15D**

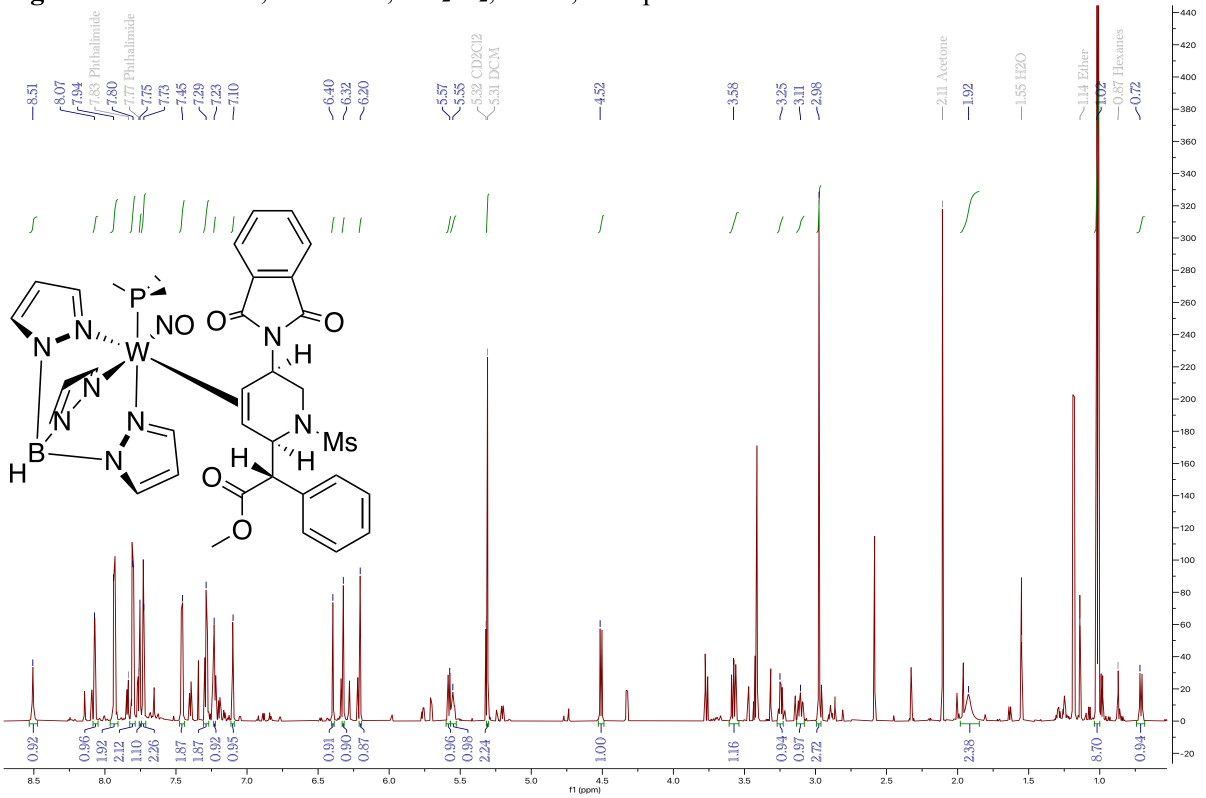

**Figure S25**: $^{13}C$ NMR, 200 MHz, $CD_2Cl_2$ , 25 °C, Compound **15D**

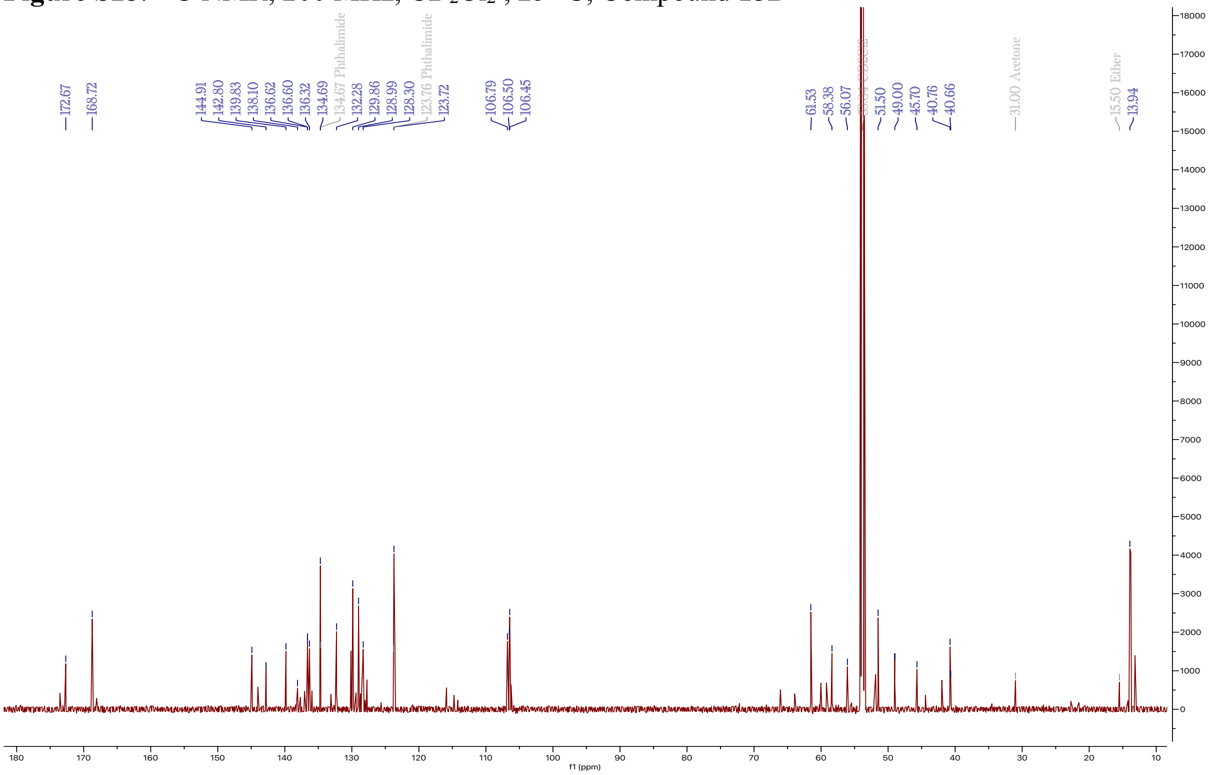

**Figure S26**: $^{1}$H NMR, 800 MHz, $CD_2Cl_2$, 25 °C, Compound **16D**

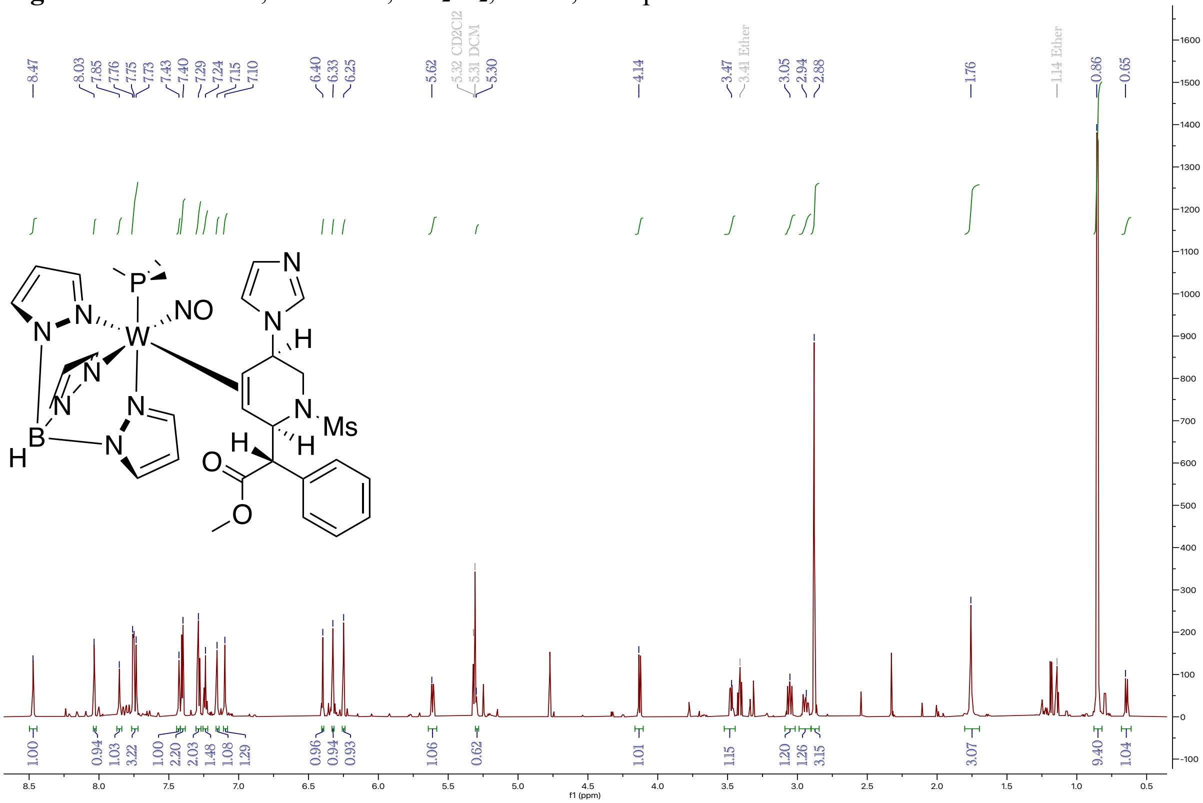


**Figure S27**: $^{13}$C NMR, 200 MHz, $CD_2Cl_2$, 25 °C, Compound **16D**

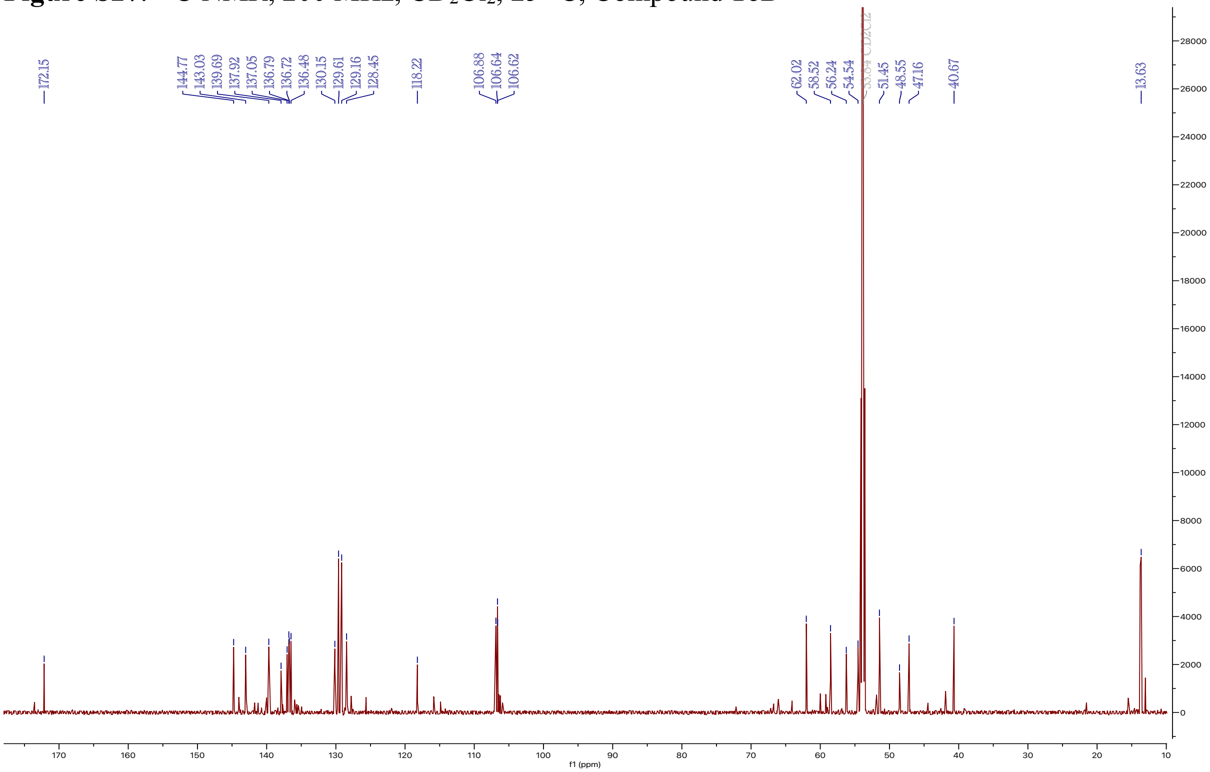

**Figure S28**: $^{1}$H NMR, 800 MHz, $CD_2Cl_2$, 25 °C, Compound **17D**

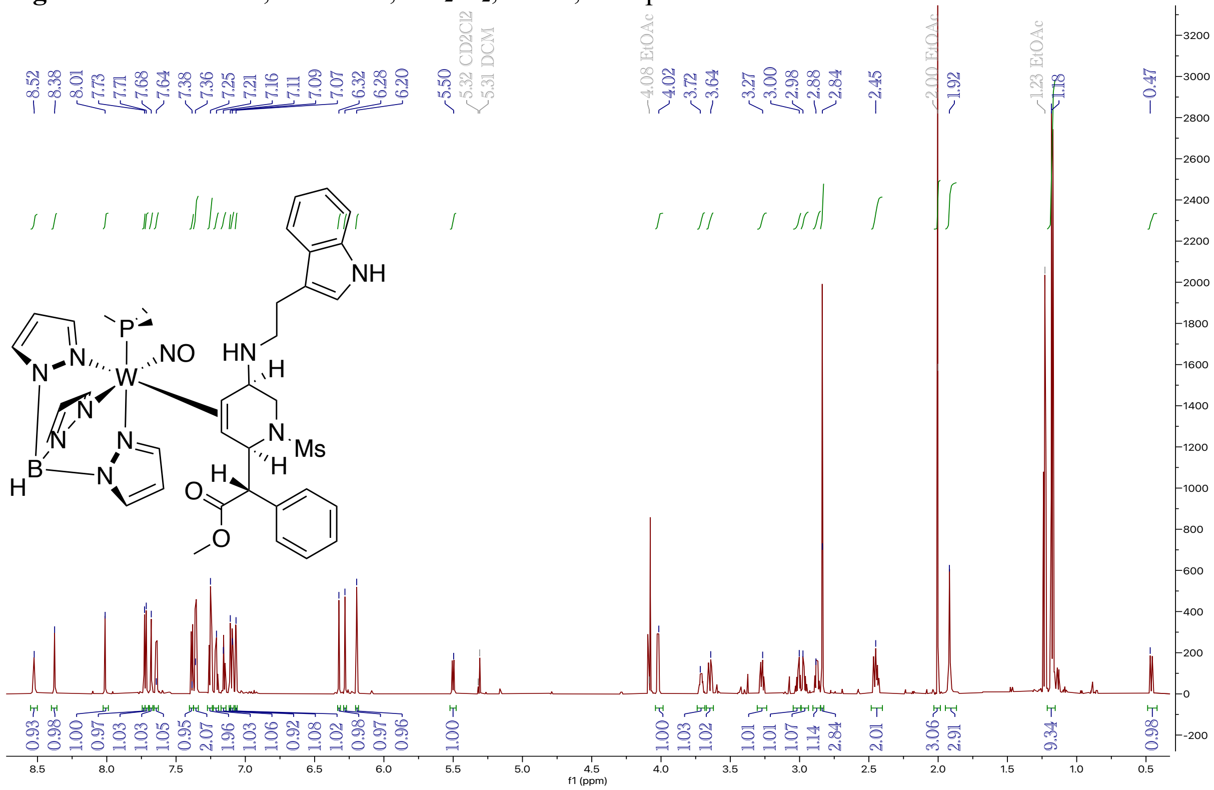


**Figure S29**: $^{13}$C NMR, 200 MHz, $CD_2Cl_2$, 25 °C, Compound **17D**

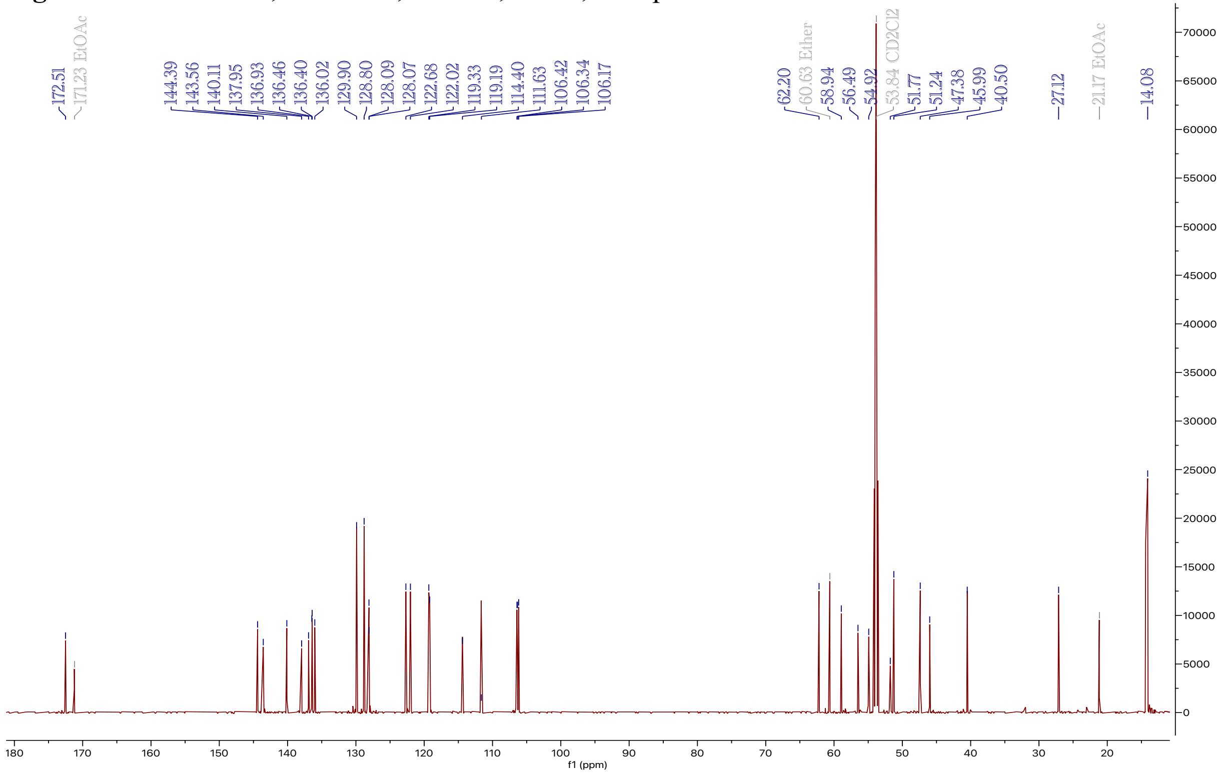

**Figure S30**: $^{1}$H NMR, 800 MHz, $CD_2Cl_2$, 25 °C, Compound **18D**

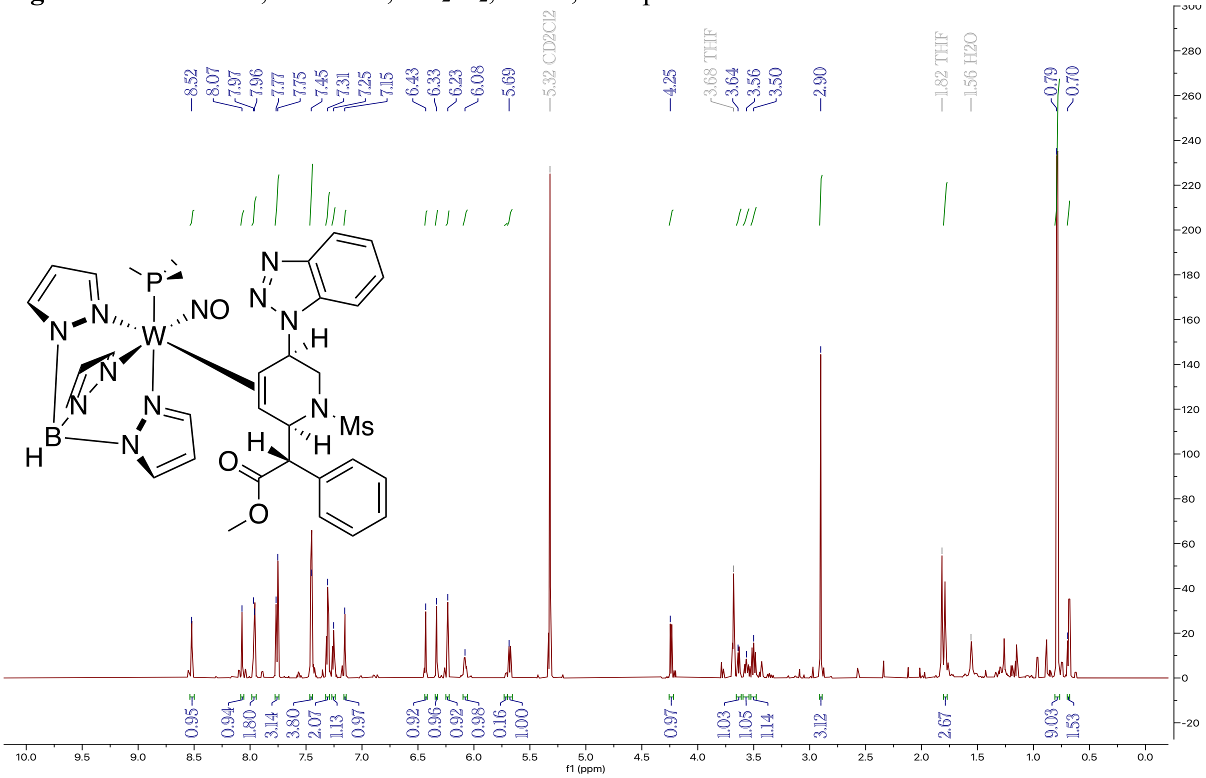

**Figure S31**: $^{13}$C NMR, 200 MHz, $CD_2Cl_2$, 25 °C, Compound **18D**

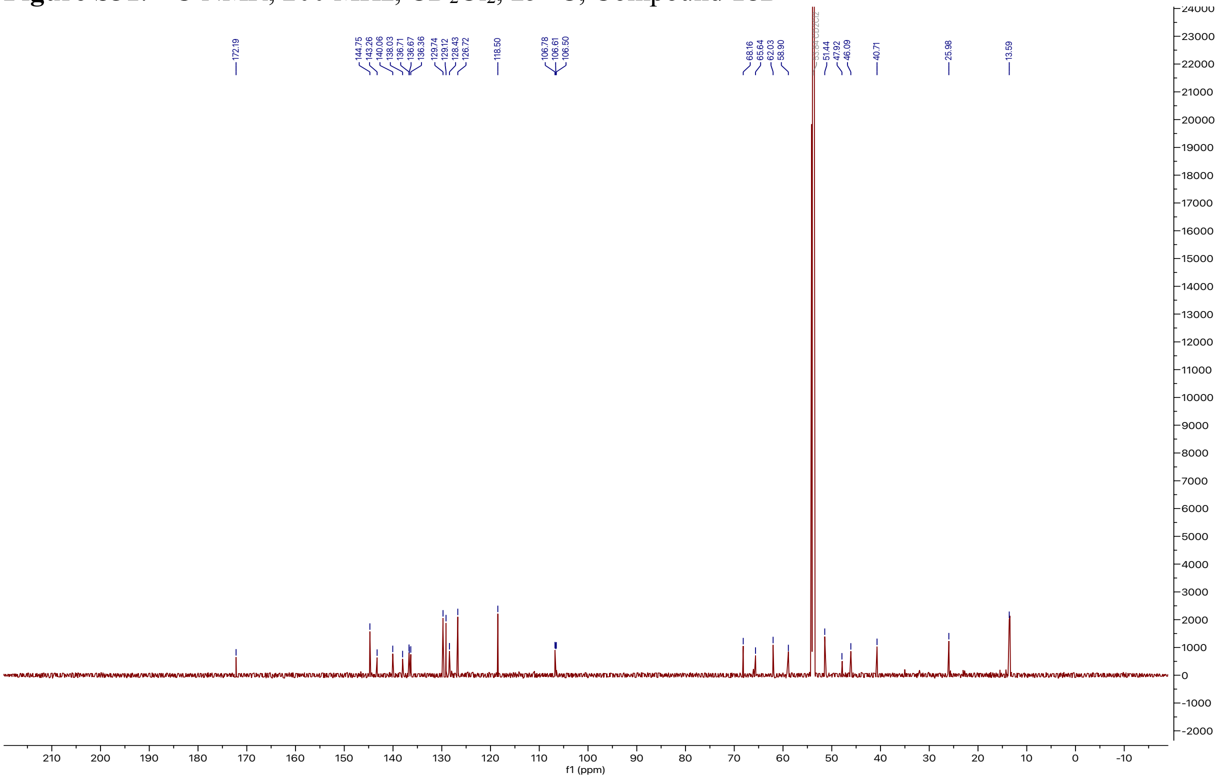

**Figure S32**: $^{1}$H NMR, 800 MHz, $(CD_3)_2CO$, 25 °C, Compound **19D**

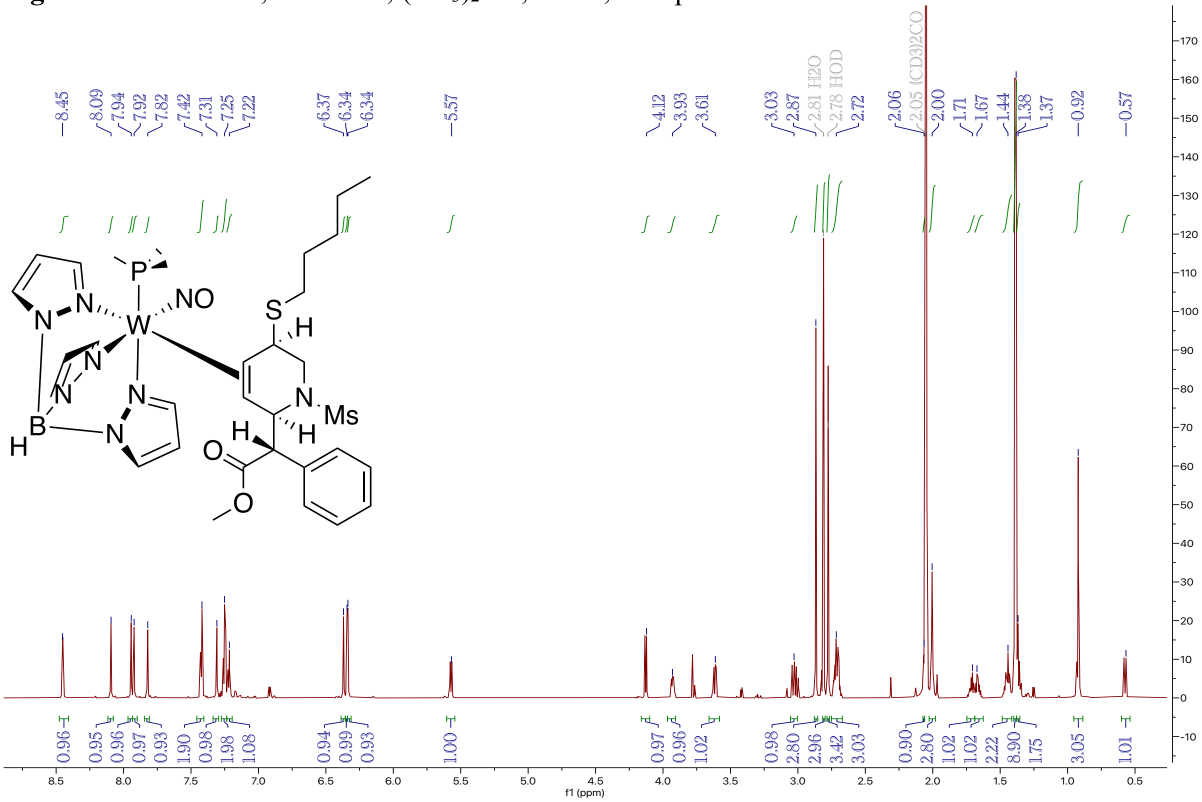

**Figure S33**: $^{13}$C NMR, 200 MHz, $(CD_3)_2CO$, 25 °C, Compound **19D**

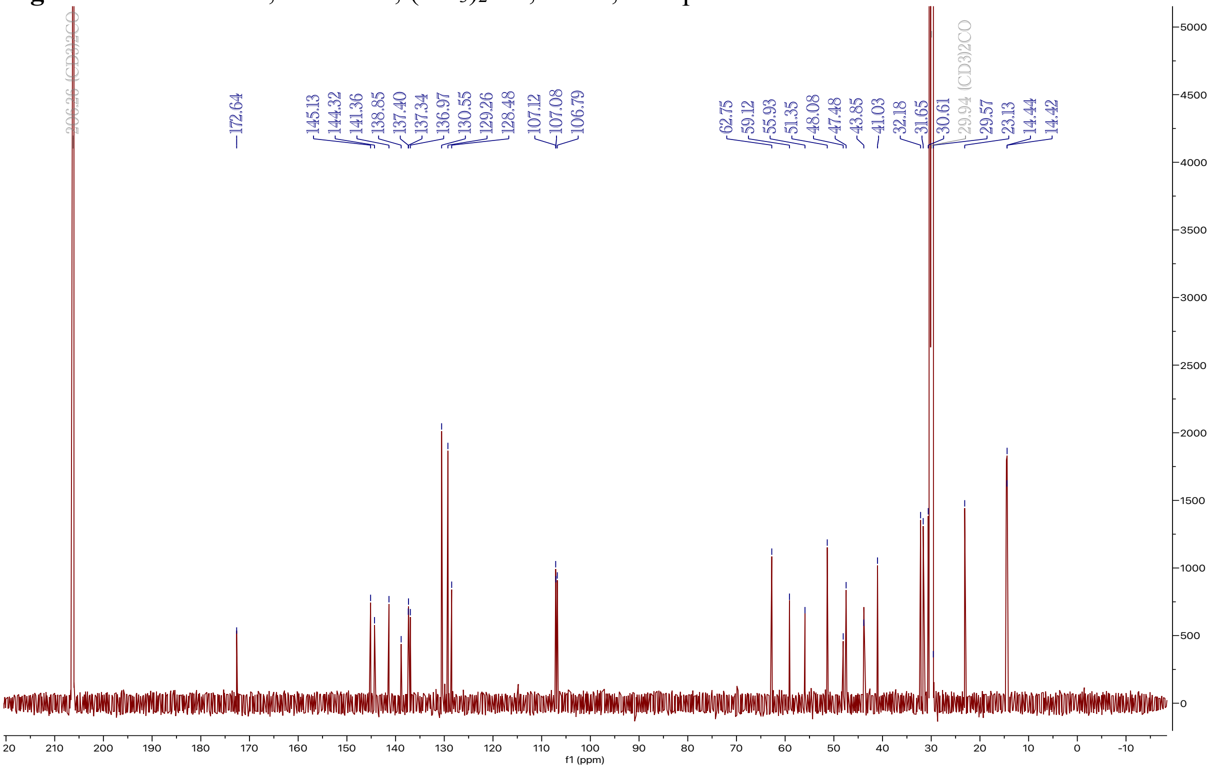

**Figure S34**: $^{1}H$ NMR, 800 MHz, $(CD_3)_2CO$ , 25 °C, Compound **20D**

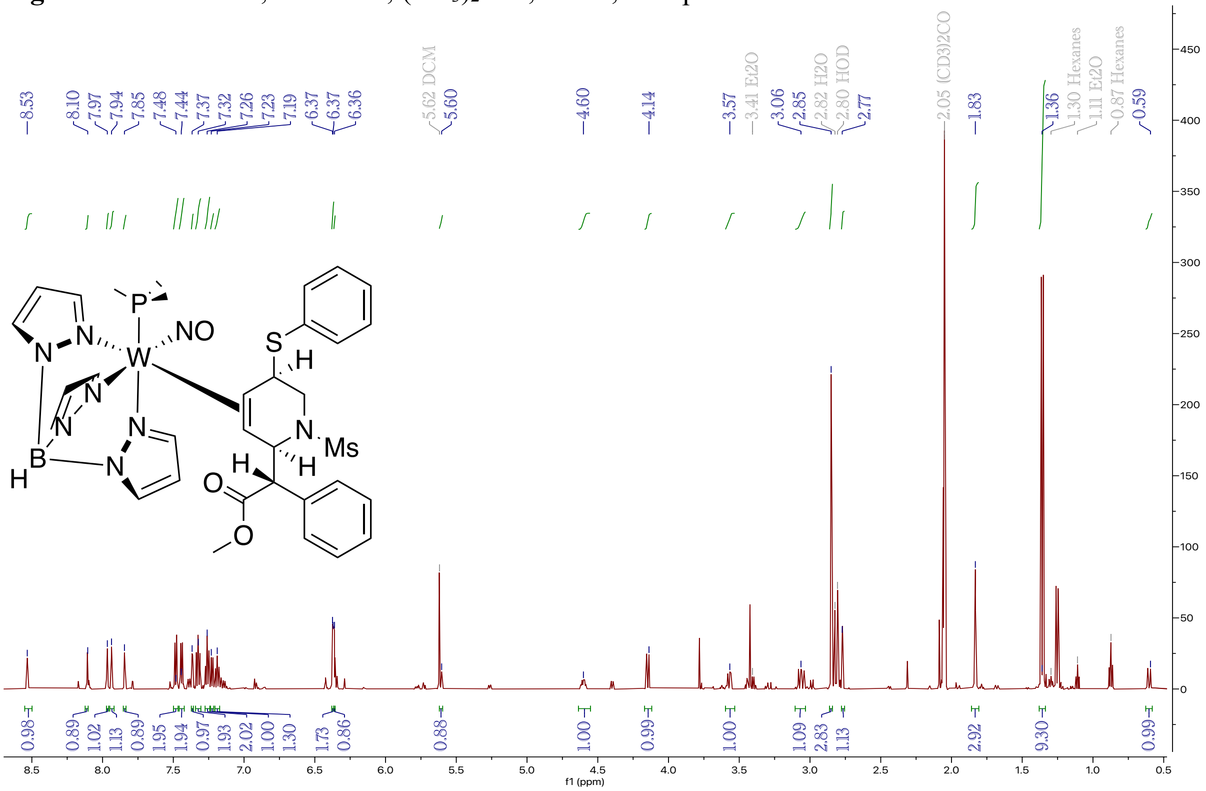


**Figure S35**: $^{13}C$ NMR, 200 MHz, $CD_2Cl_2$, 25 °C, Compound **20D**

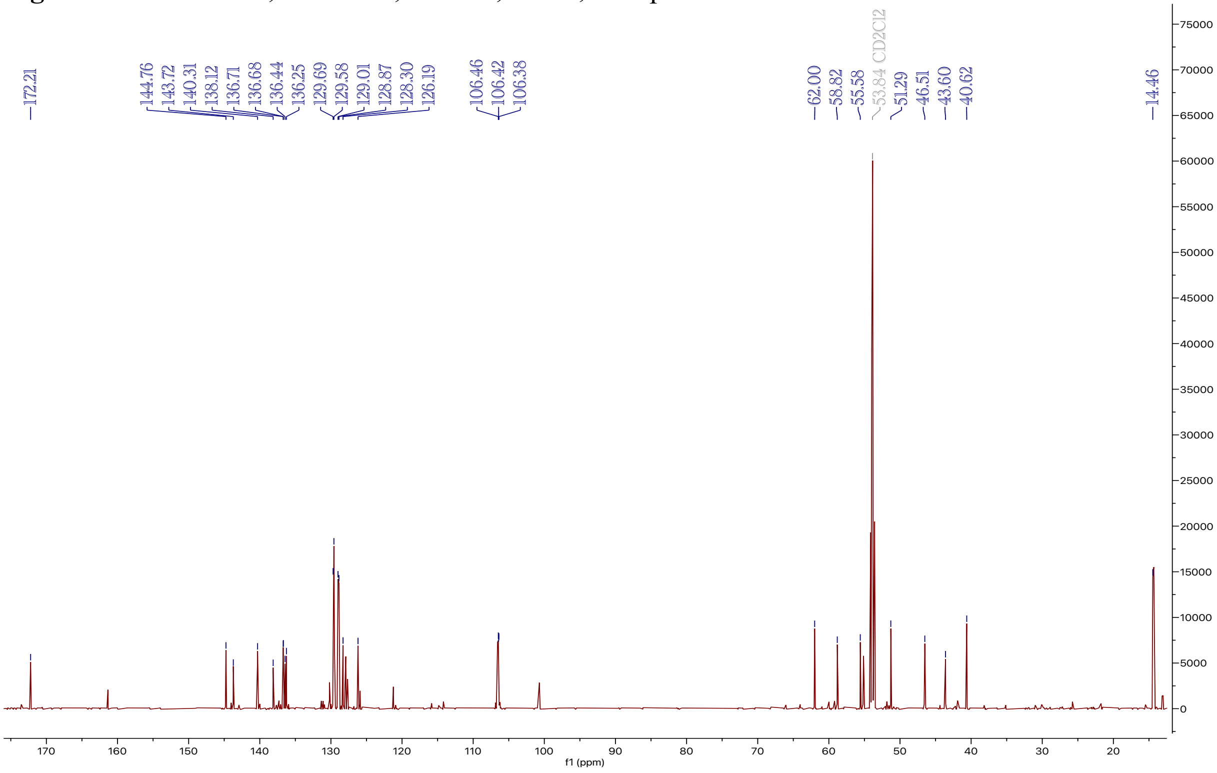

**Figure S36**: $^{1}$H NMR, 800 MHz, $(CD_3)_2CO$, 25 °C, Compound **21-Ms**

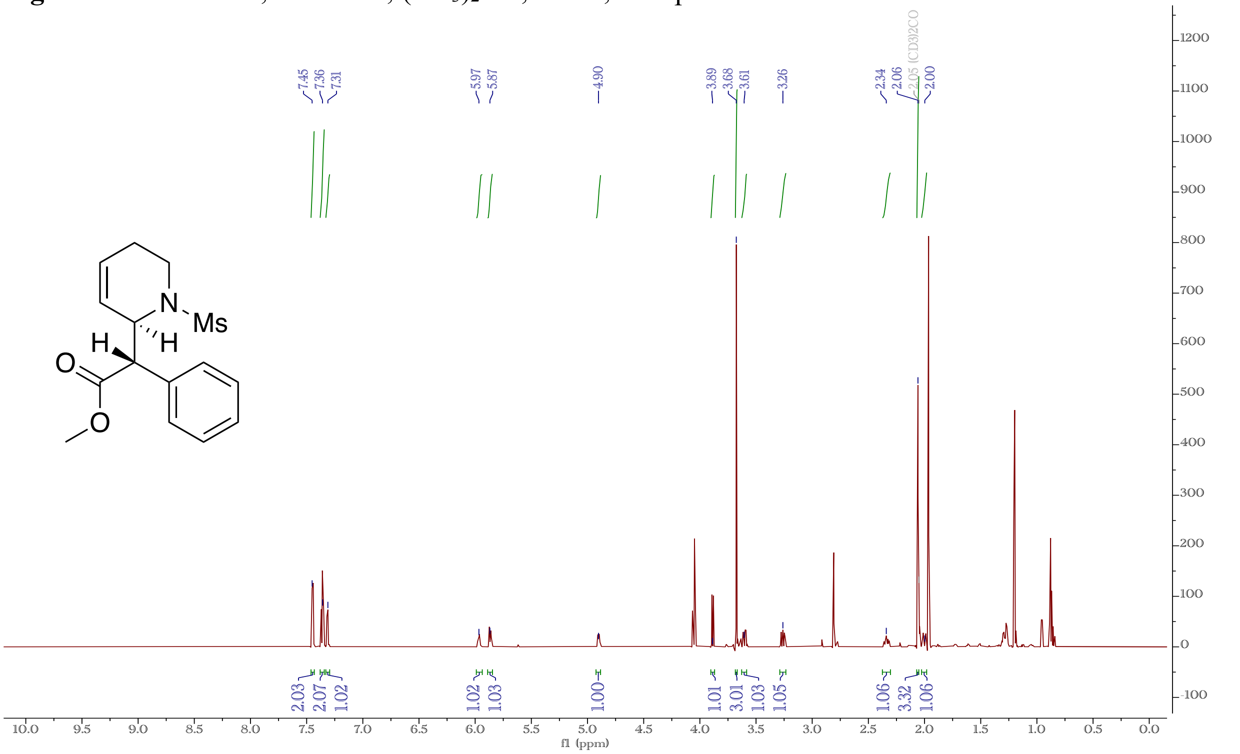


**Figure S37**: $^{13}$C NMR, 200 MHz, $(CD_3)_2CO$, 25 °C, Compound **21-Ms**

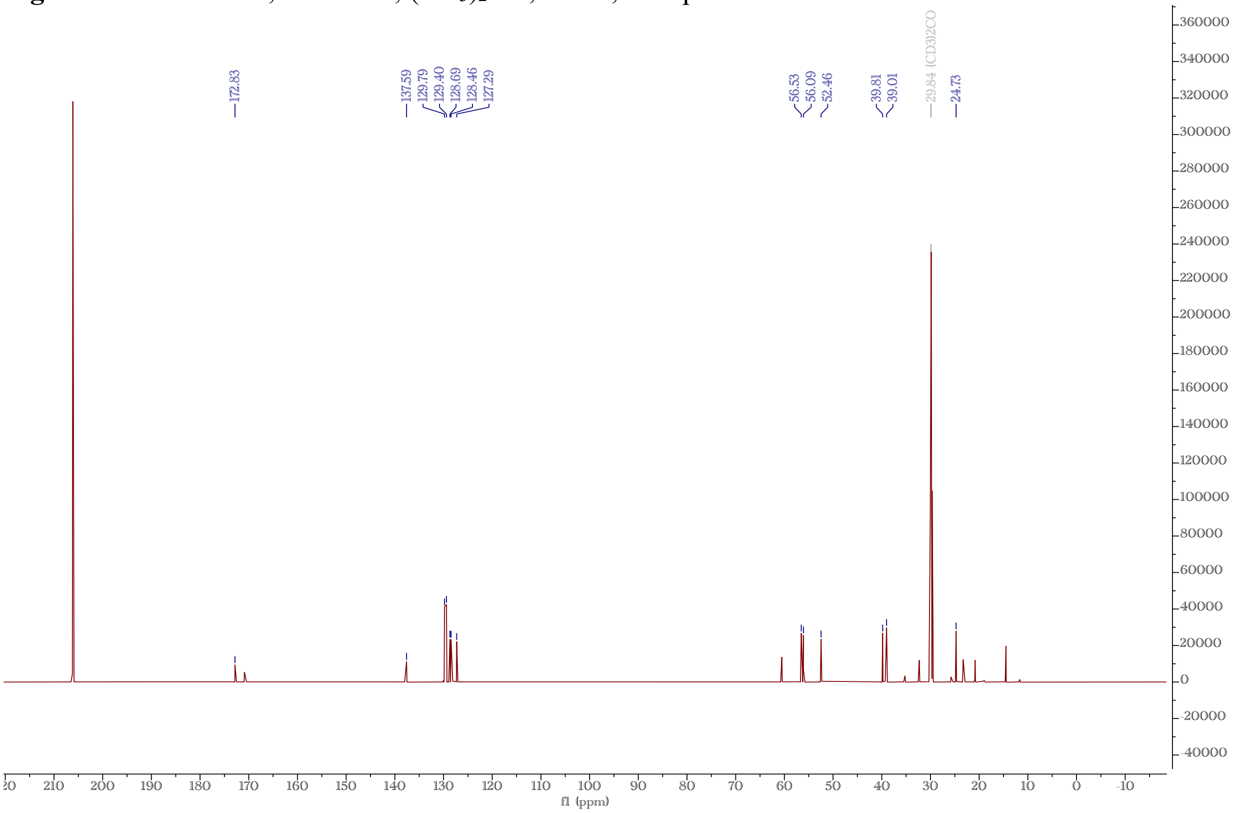

**Figure S38**: $^1$H NMR, 800 MHz, $CDCl_3$, 25 °C, Compound **21-Ac**

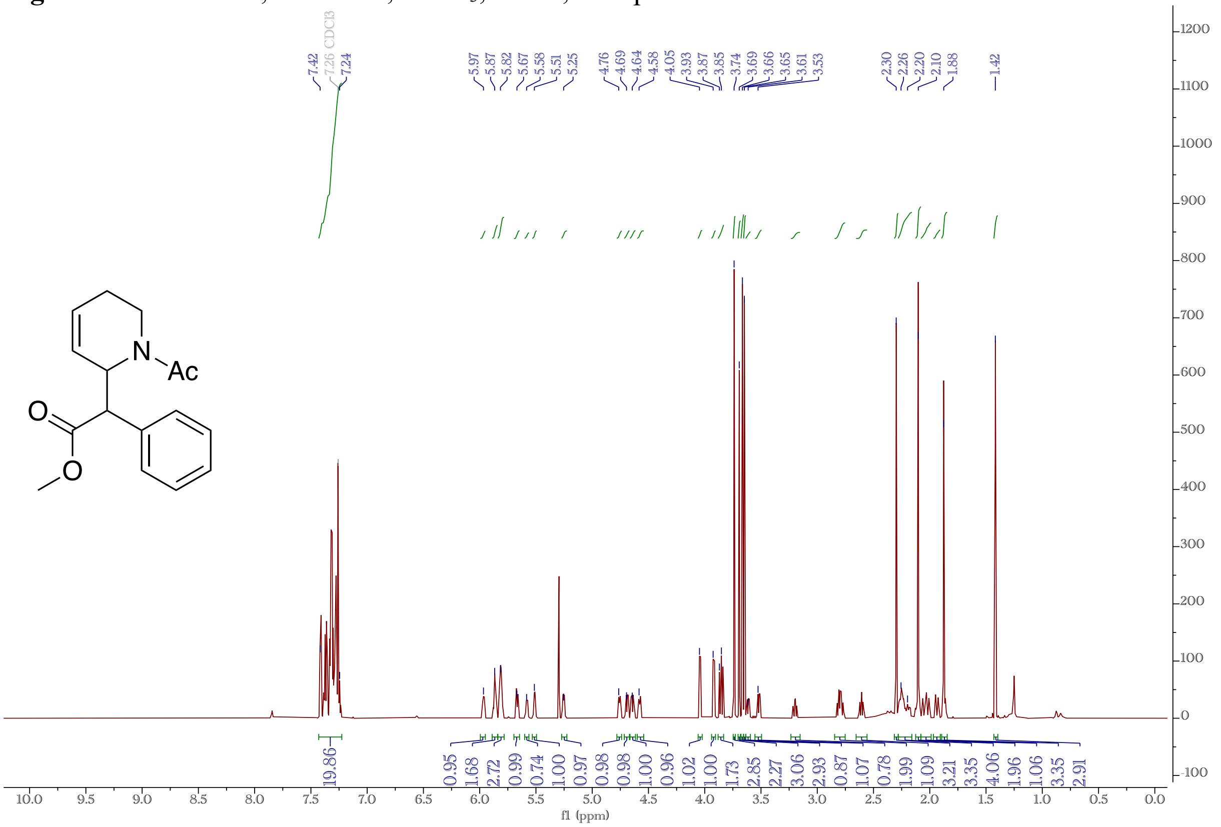


**Figure S39**: $^{13}$C NMR, 200 MHz, $CDCl_3$, 25 °C, Compound **21-Ac**

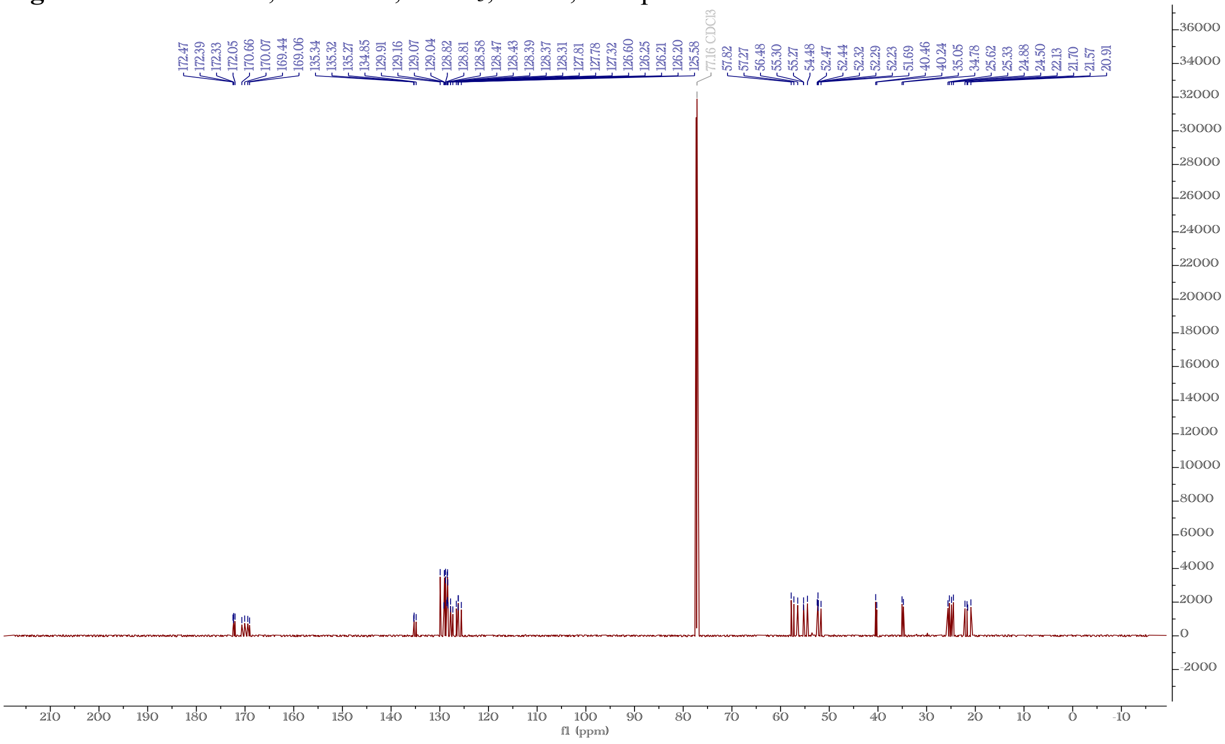

**Figure S40**: $^{1}$H NMR, 800 MHz, $CDCl_3$, 25 °C, Compound **21-Ts**

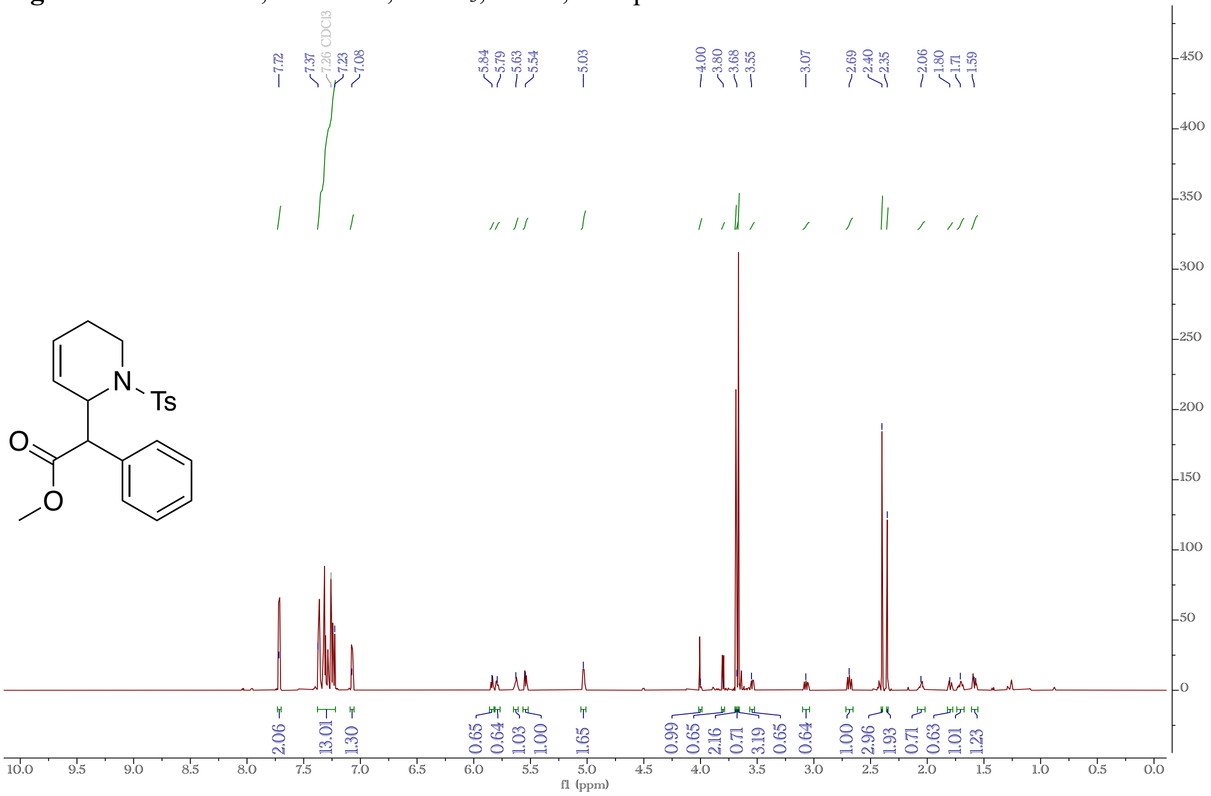


**Figure S41**: $^{13}$C NMR, 200 MHz, $CDCl_3$, 25 °C, Compound **21-Ts**

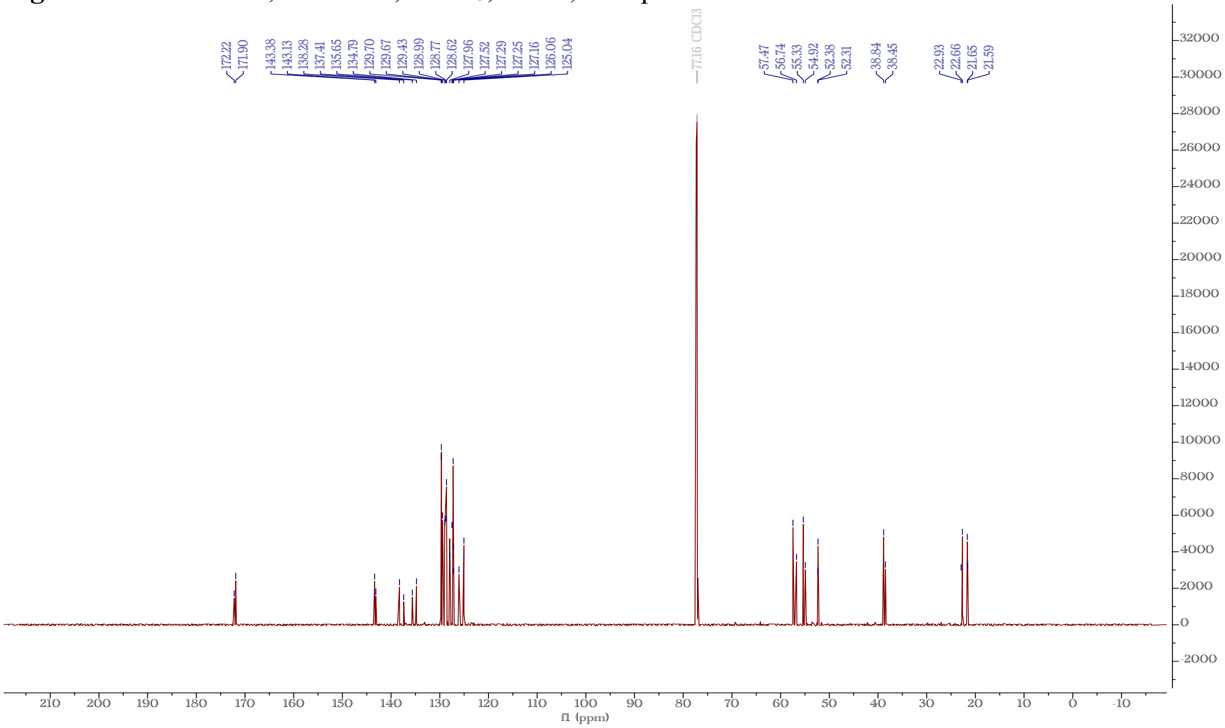

**Figure S42**: $^{1}$H NMR, 800 MHz, $(CD_3)_2CO$, 25 °C, Compound **22**

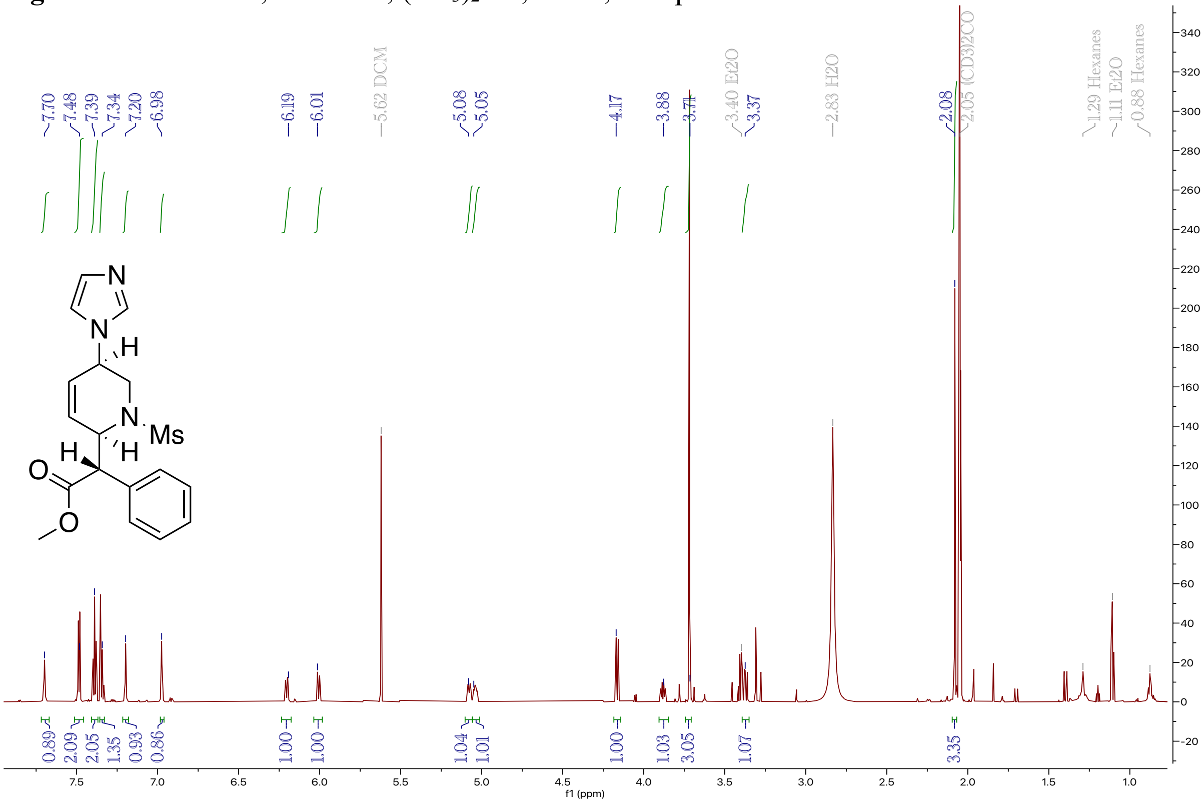


**Figure S43**: $^{13}$C NMR, 200 MHz, $(CD_3)_2CO$, 25 °C, Compound **22**

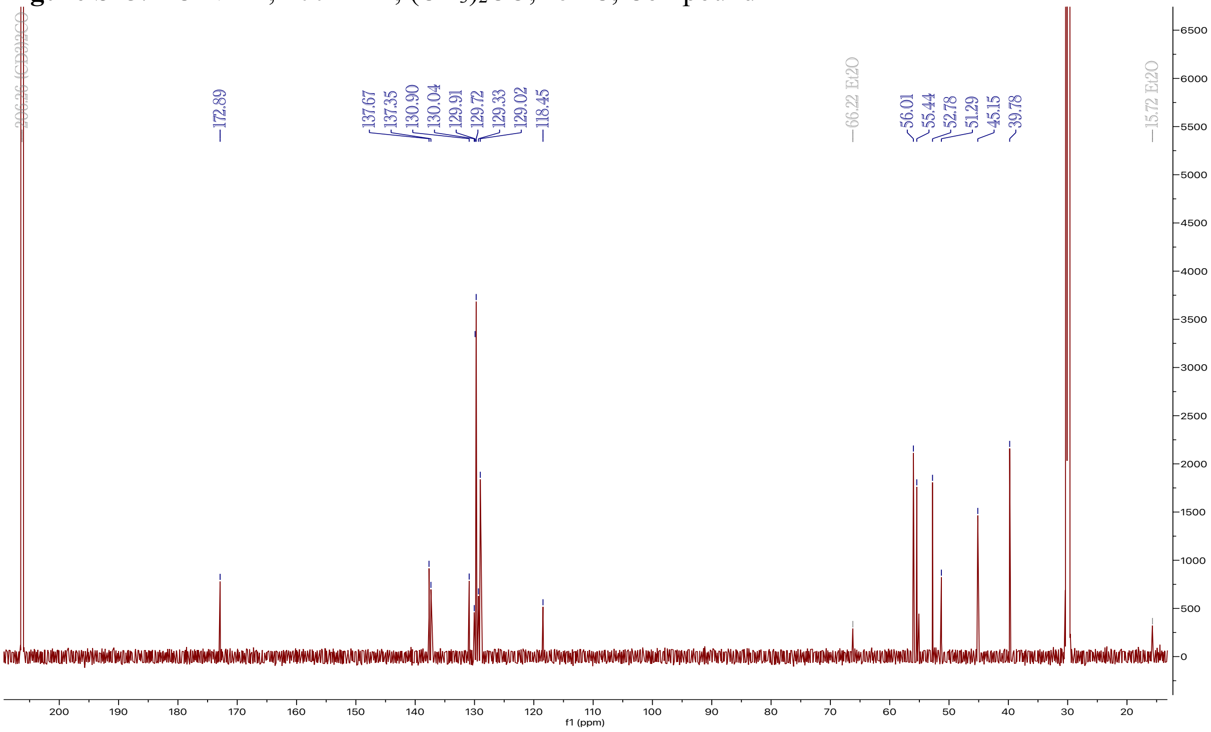

**Figure S44**: $^{1}$H NMR, 800 MHz, $CDCl_3$, 25 °C, Compound **23**

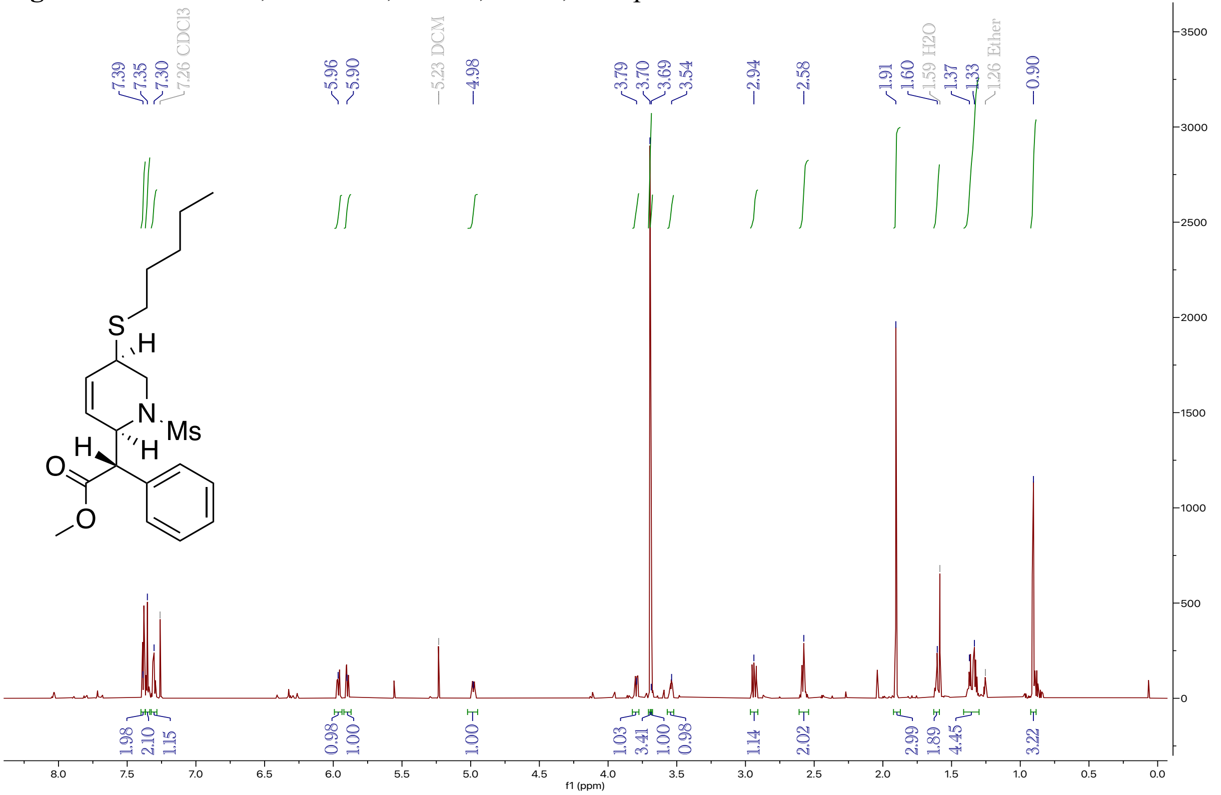

**Figure S45**: $^{13}$C NMR, 200 MHz, $CDCl_3$, 25 °C, Compound **23**

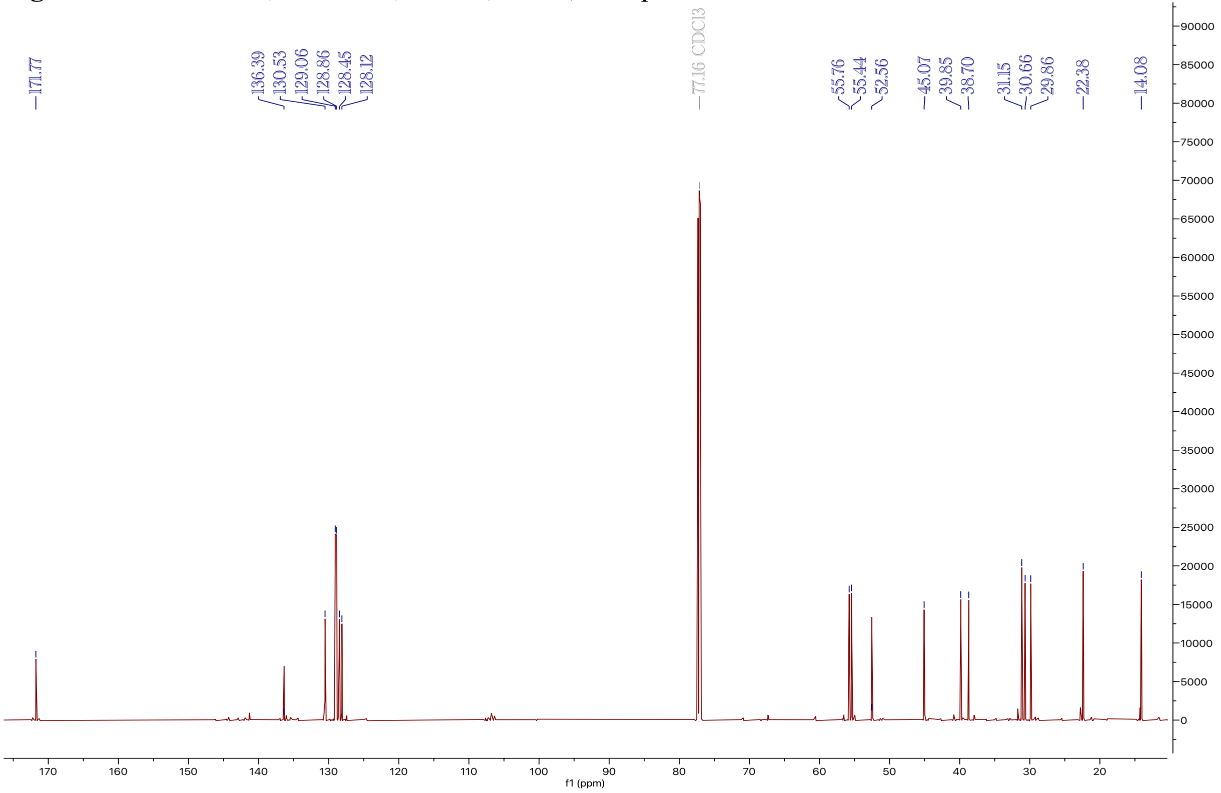

**Figure S46**: $^{1}$H NMR, 800 MHz, $(CD_3)_2CO$, 25 °C, Compound **24**

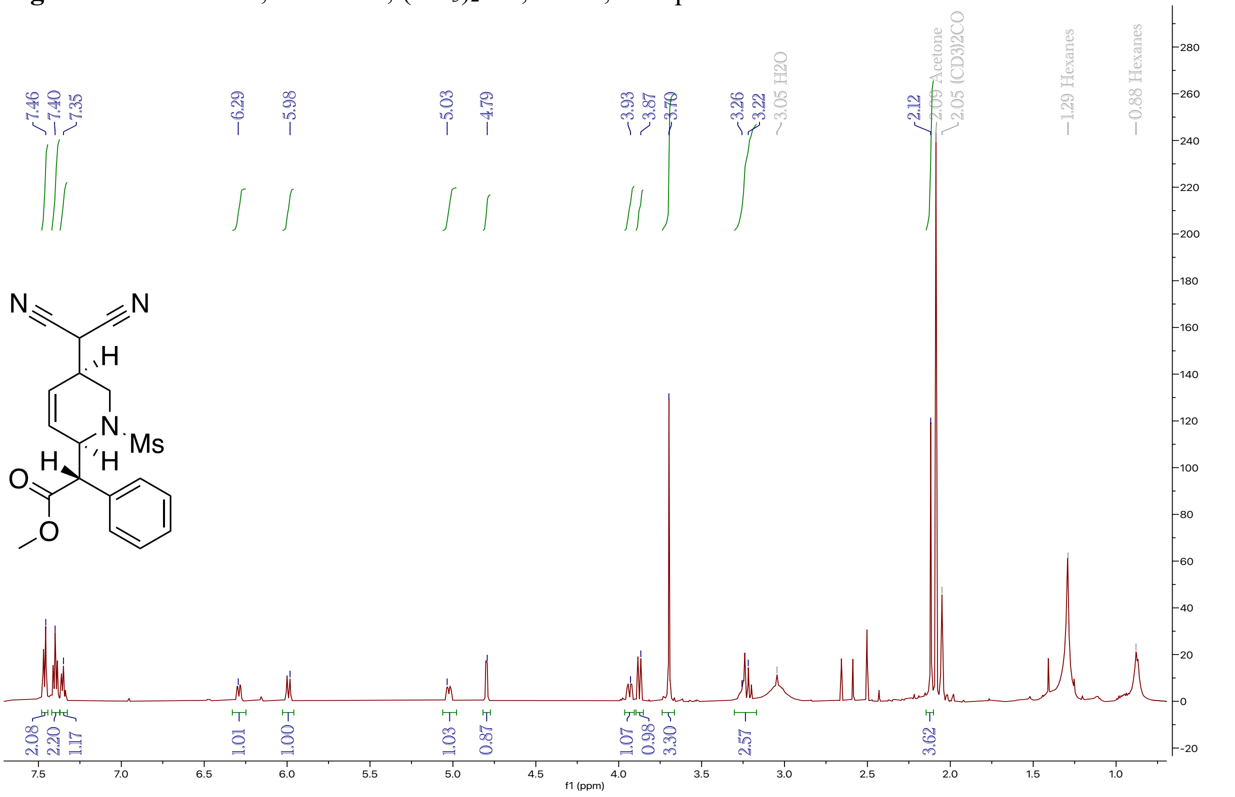

**Figure S47**: $^{13}$C NMR, 200 MHz, $(CD_3)_2CO$, 25 °C, Compound **24**

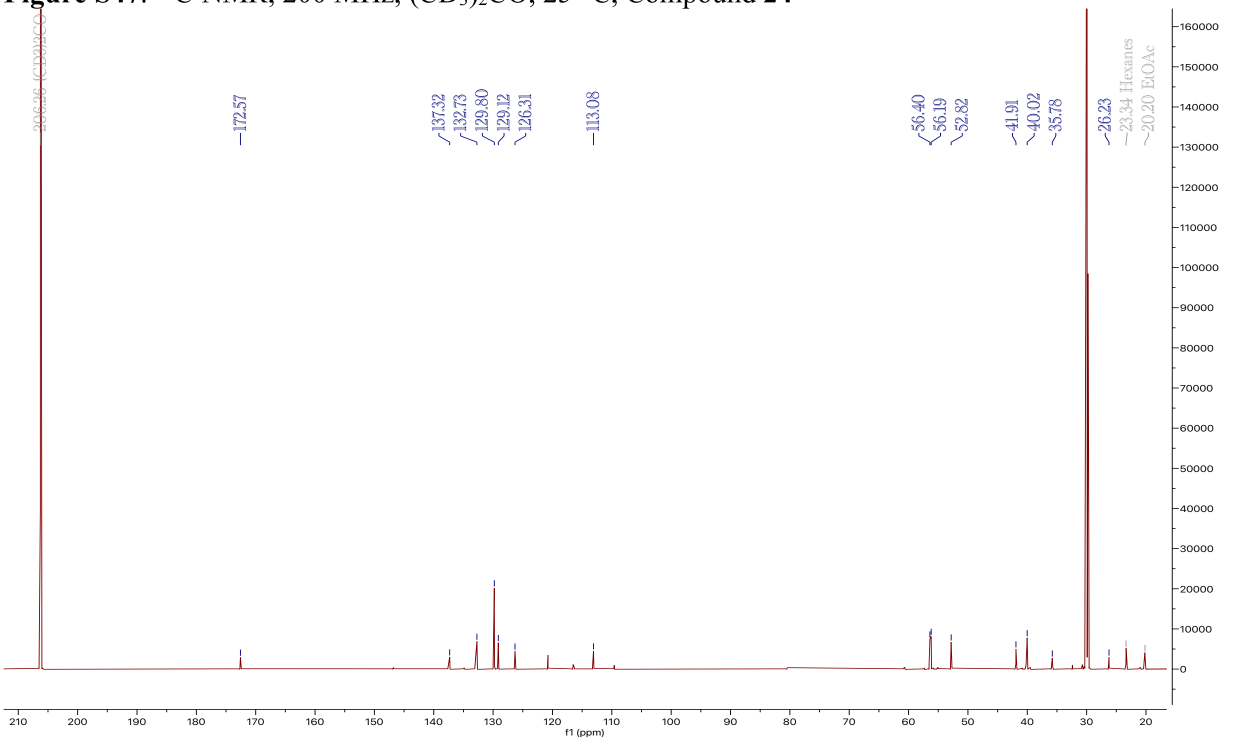

**Figure S48**: $^{1}H$ NMR, 800 MHz, $(CD_3)_2CO$, 25 °C, Compound **25**

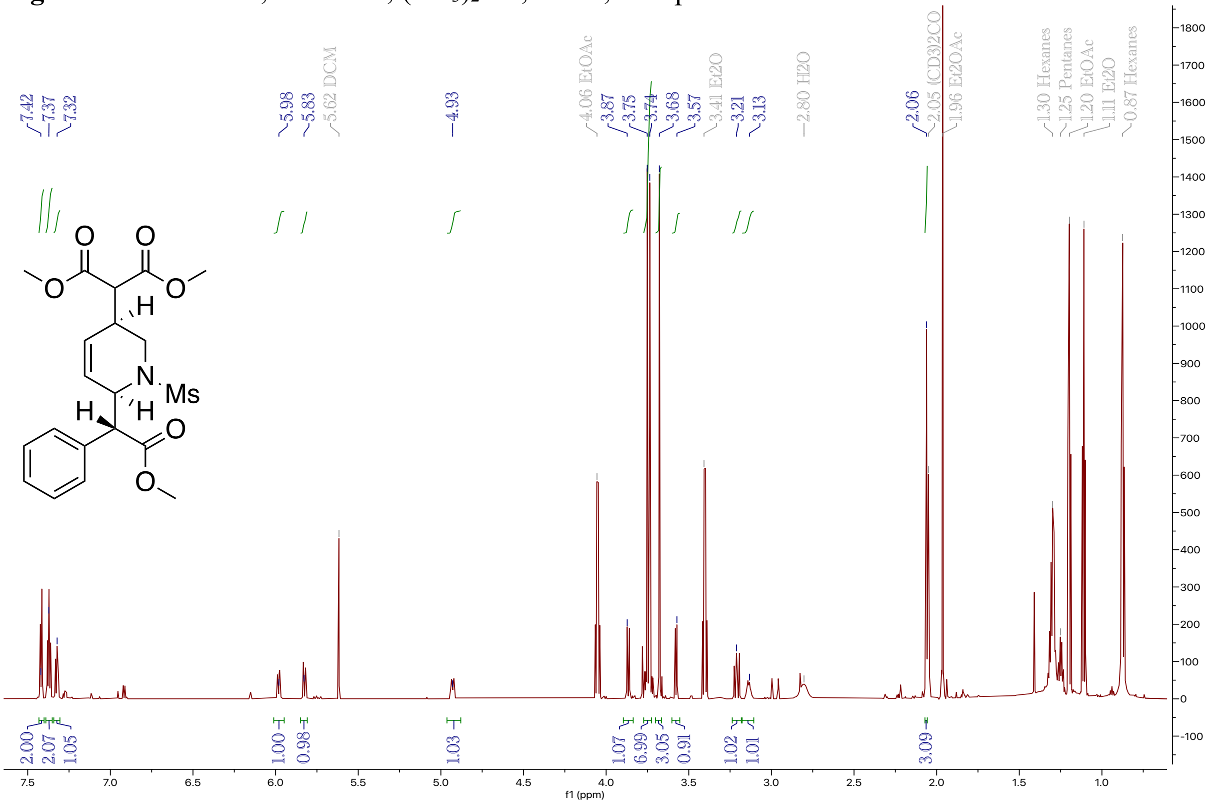


**Figure S49**: $^{13}C$ NMR, 200 MHz, $(CD_3)_2CO$, 25 °C, Compound **25**

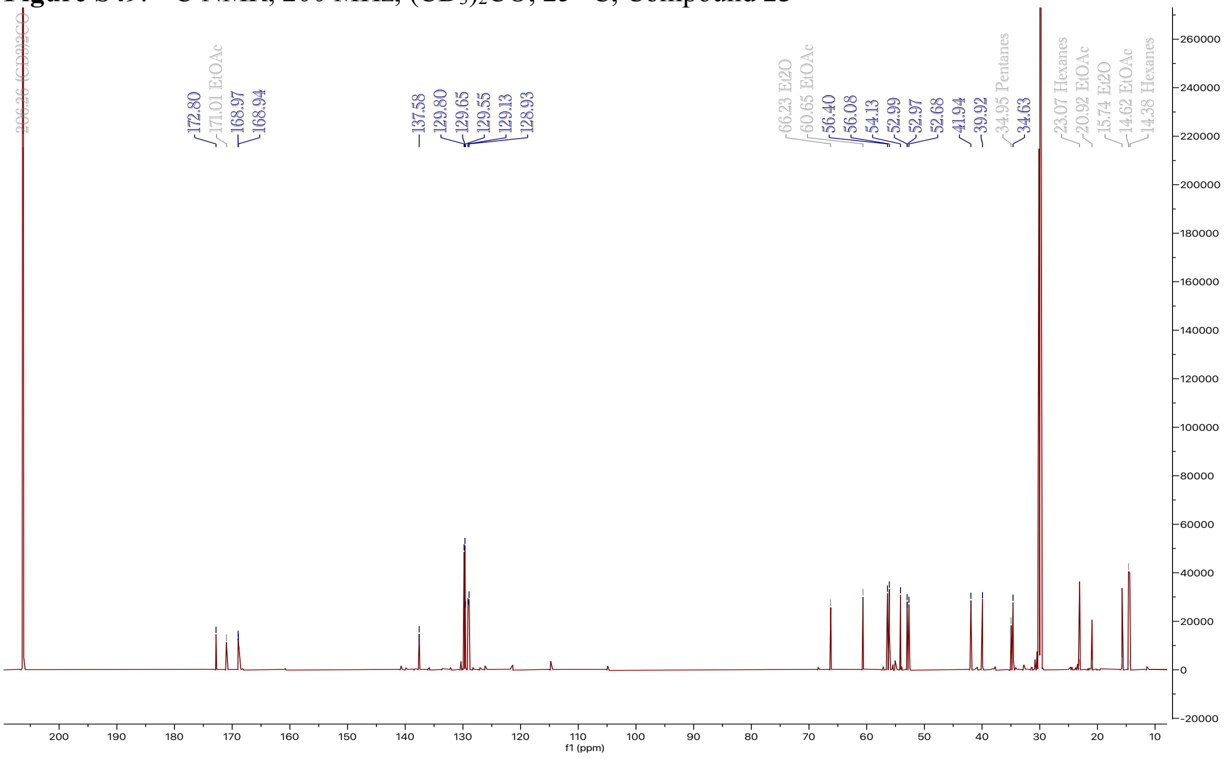

**Figure S50**: $^{1}$H NMR, 800 MHz, $(CD_3)_2CO$, 25 °C, Compound **26**

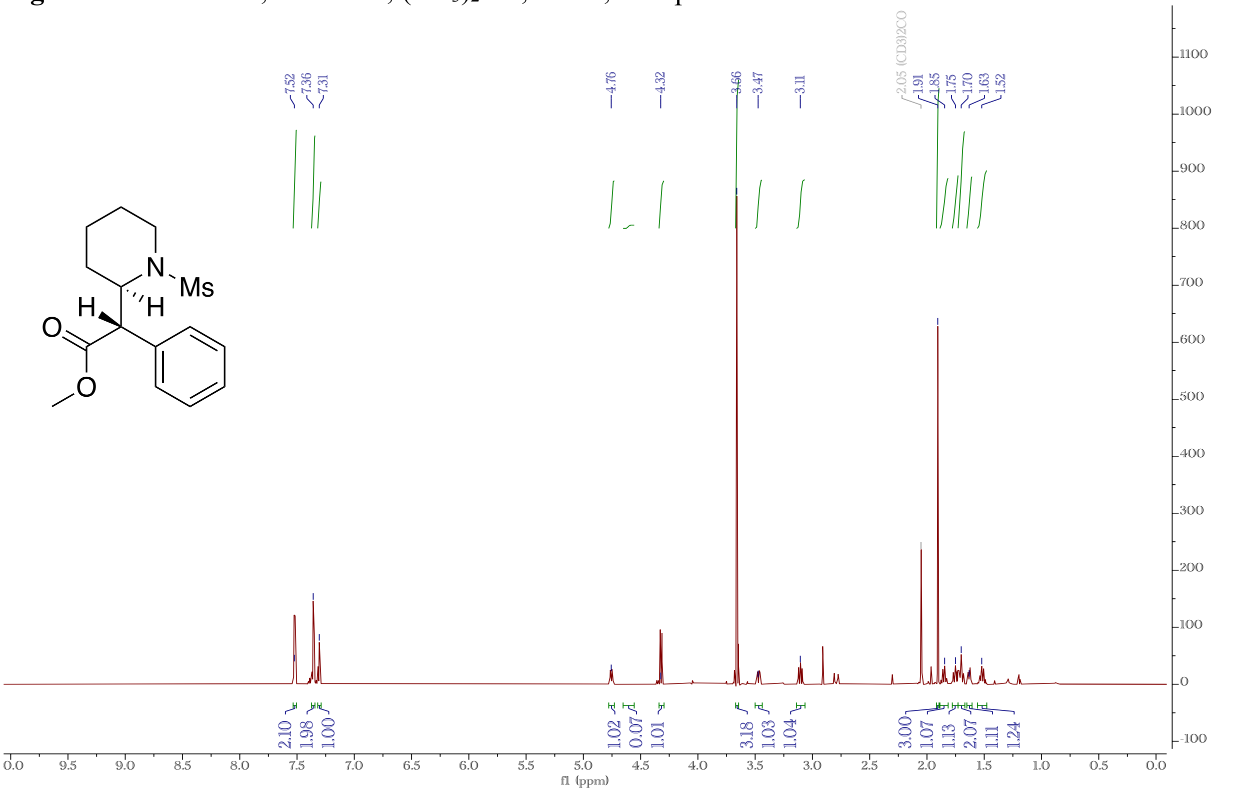


**Figure S51**: $^{13}$C NMR, 200 MHz, $(CD_3)_2CO$, 25 °C, Compound **26**

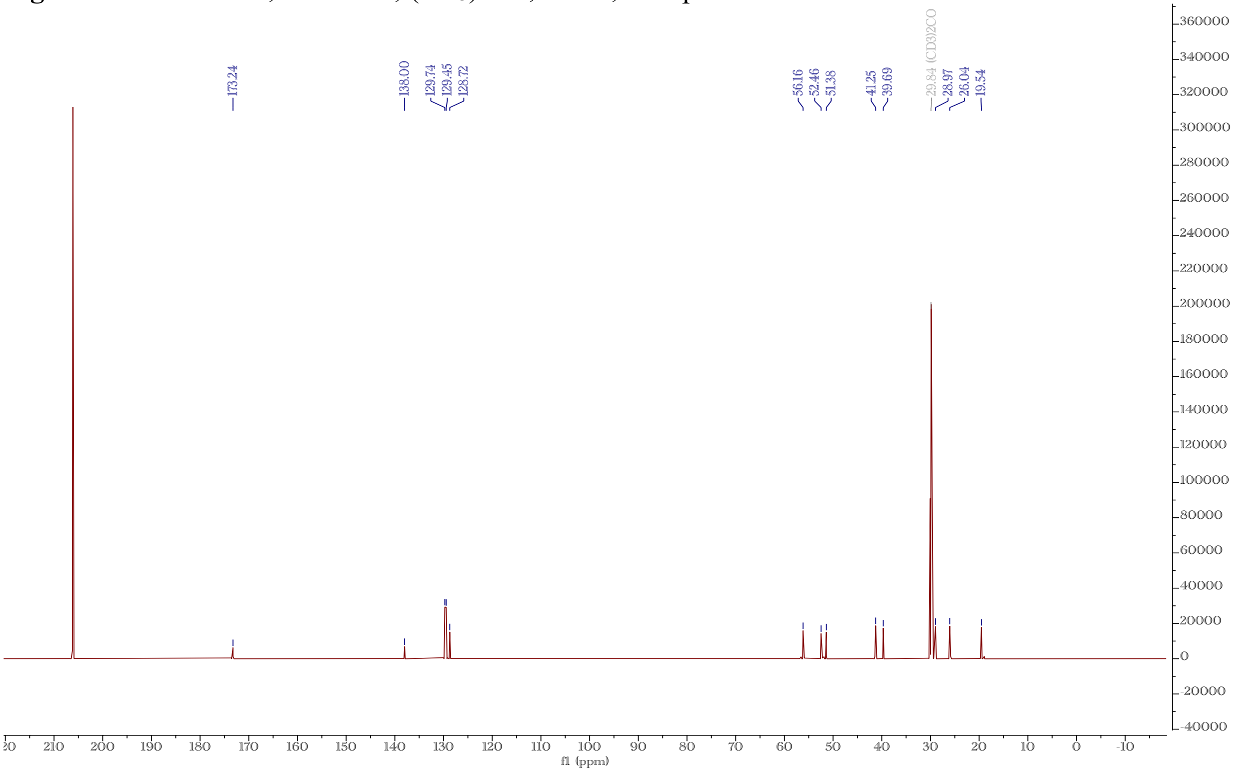

**Figure S52**: $^{1}$H NMR, 800 MHz, $CD_2Cl_2$, 25 °C, 3:1 ratio of Compound **26** to Compound **21'.**

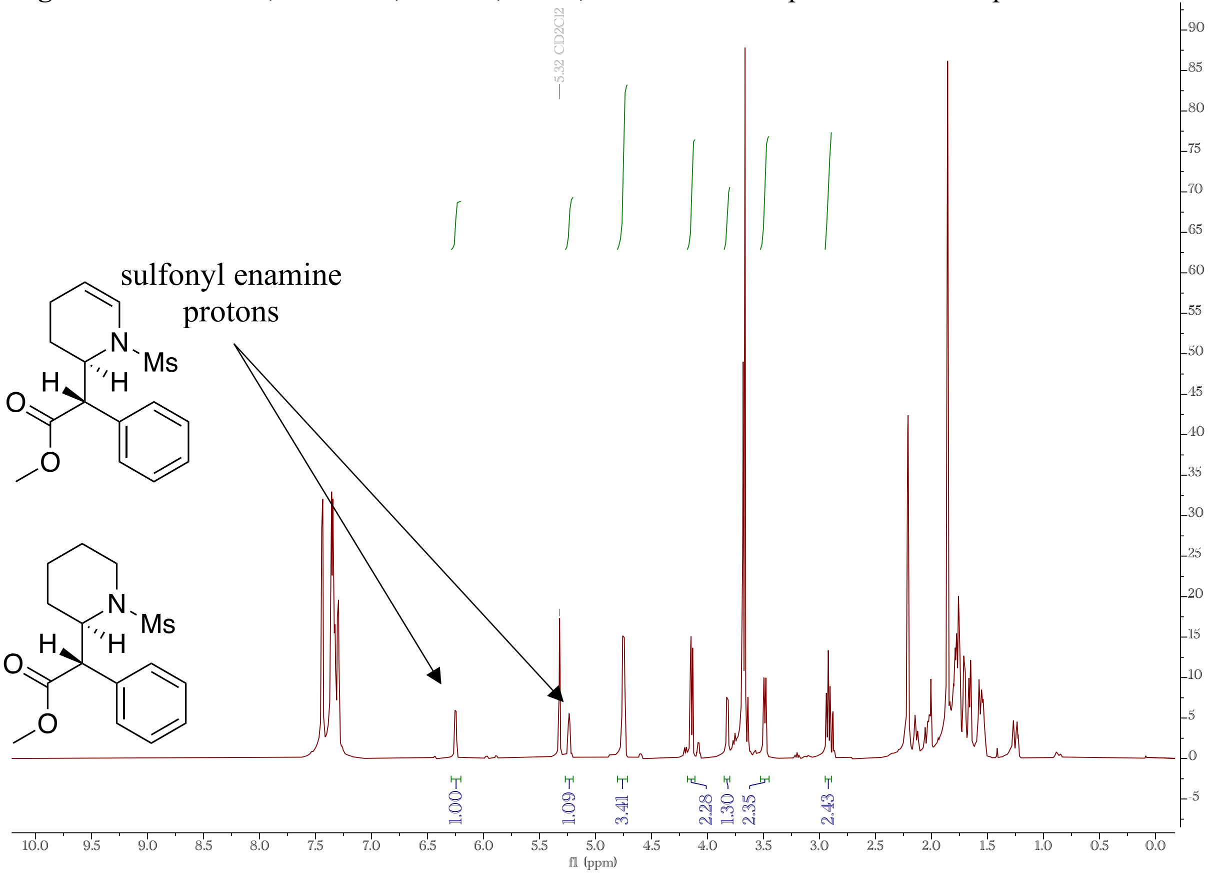

**Figure S53**: $^{1}$H NMR, 800 MHz, $CD_2Cl_2$, 25 °C, Compound **27**

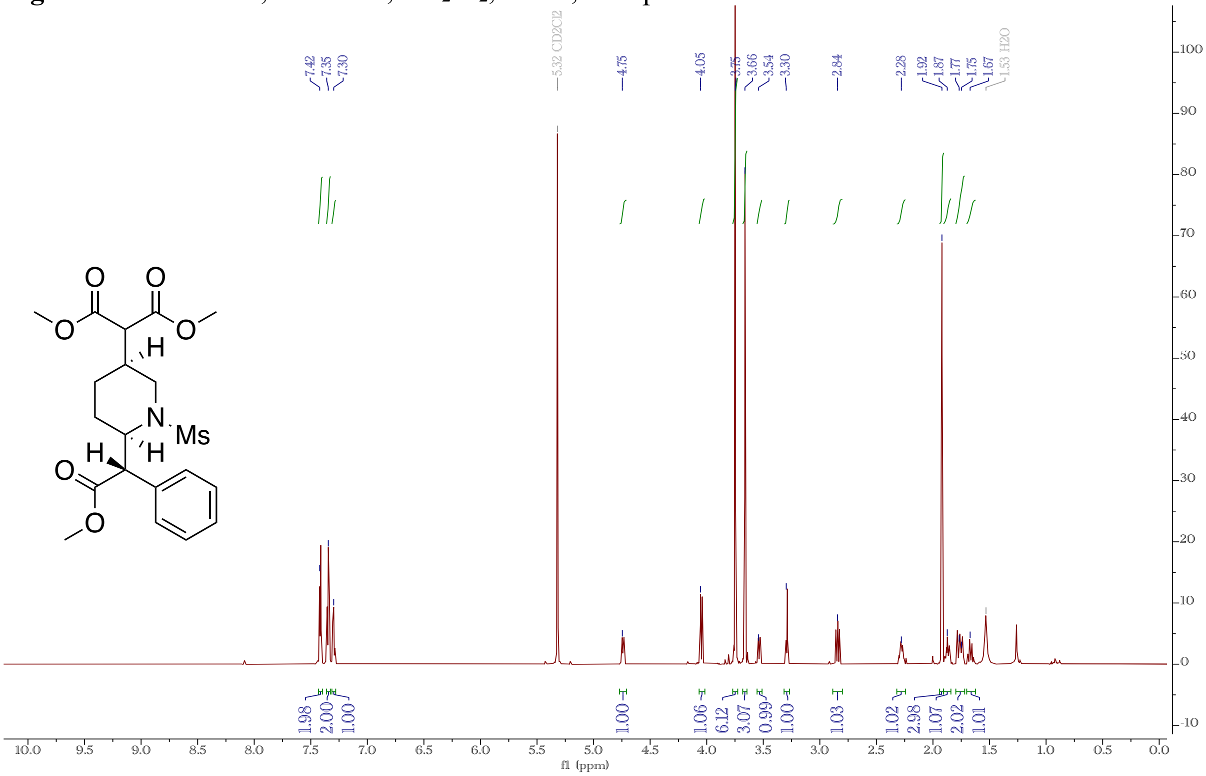


**Figure S54**: $^{13}$C NMR, 200 MHz, $CD_2Cl_2$, 25 °C, Compound **27**

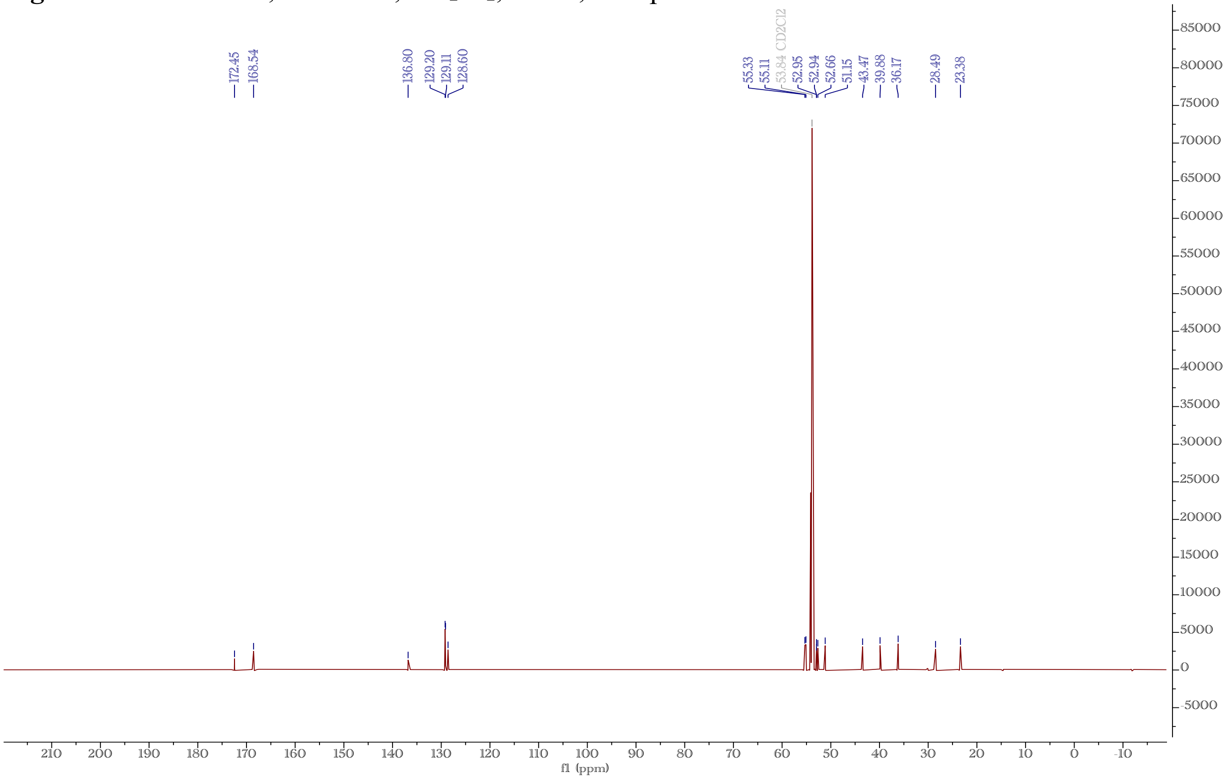

**Computational Methods and DFT Analysis for 7D.**

Ground-state structures were optimized at the M06 level of theory using the 6-31G** [LANL2DZ for W] basis set in Gaussian 16. Conformational analysis was performed by optimizing multiple conformations and determining the lowest energy conformation. Previous literature demonstrates that this functional and basis set choice accurately corroborates experimental results. Vibrational frequency analysis verified that optimized structures were minima, and rigid-rotor-harmonic-oscillator thermochemical chemical corrections were applied at 298 K and 1 atm utilizing Gaussian's default implementation. Bond lengths were used to determine the likelihood of spatial interactions. 2D NMR analysis was performed on a 3 erythro : 1 threo sample using $CD_2Cl_2$ as the solvent.

**Table S1: DFT Geometry Optimization, Energies and Coordinates for *erythro*-7D.**
Electronic Energy: -2695.646348 Hartree
Electronic Energy + Free Energy Correction: -2695.095613 Hartree

| | | | |
|---|---|---|---|
| W | -1.277300 | -0.358800 | -0.127400 |
| S | 3.133200 | -2.164600 | 0.838600 |
| O | 2.402400 | 3.114100 | -1.759800 |
| O | 3.555700 | -1.287700 | 1.921500 |
| O | -0.812300 | -3.271500 | 0.209300 |
| O | 2.635700 | 3.087700 | 0.473200 |
| O | 4.080800 | -2.982800 | 0.095900 |
| N | -0.871400 | -2.065200 | 0.113500 |
| N | 2.298600 | -1.207900 | -0.256700 |
| C | 0.651400 | 0.573900 | -0.489800 |
| C | 0.228400 | 0.027500 | -1.762900 |
| H | -0.113300 | 0.720500 | -2.537900 |
| C | 0.987300 | -1.135400 | -2.253000 |
| H | 0.785800 | -1.536700 | -3.245700 |
| C | 2.041200 | 0.209100 | -0.012300 |
| H | 2.184200 | 0.408700 | 1.055800 |
| C | 1.935100 | -1.733100 | -1.511100 |
| H | 2.494700 | -2.608600 | -1.830900 |
| C | 3.089800 | 1.073700 | -0.787400 |
| H | 2.969000 | 0.837000 | -1.852100 |
| C | 4.511300 | 0.775800 | -0.376900 |
| C | 2.711200 | 2.518500 | -0.592800 |
| C | 5.255800 | -0.117200 | -1.147900 |
| H | 4.811100 | -0.543800 | -2.047000 |
| C | 6.364900 | 0.939900 | 1.162800 |
| H | 6.794700 | 1.353000 | 2.072900 |
| C | 1.897900 | -3.265800 | 1.488300 |
| H | 2.418700 | -3.970700 | 2.141200 |
| H | 1.397000 | -3.781400 | 0.666400 |
| H | 1.163100 | -2.683100 | 2.049300 |
| C | 6.539700 | -0.488000 | -0.764800 |
| H | 7.099500 | -1.198500 | -1.368400 |
| C | 1.950200 | 4.457200 | -1.636400 |
| H | 2.704700 | 5.082200 | -1.149100 |
| H | 1.031500 | 4.500000 | -1.039300 |
| H | 1.761700 | 4.810000 | -2.651600 |
| C | 5.078400 | 1.302700 | 0.785800 |
| H | 4.498100 | 1.989300 | 1.397900 |
| C | 7.098100 | 0.042300 | 0.392200 |
| H | 8.101200 | -0.247700 | 0.697200 |
| H | 0.457400 | 1.644000 | -0.352400 |
| P | -2.574400 | -1.049400 | -2.165200 |
| C | -4.395800 | -0.820100 | -2.039600 |
| H | -4.806900 | -1.422700 | -1.224600 |
| H | -4.890100 | -1.094100 | -2.978900 |
| H | -4.600100 | 0.234700 | -1.816200 |
| C | -2.286100 | -0.253900 | -3.796200 |

| | | | |
|---|---|---|---|
| H | -2.493400 | 0.819800 | -3.730900 |
| H | -2.956700 | -0.694700 | -4.543000 |
| H | -1.251100 | -0.394100 | -4.120500 |
| C | -2.380700 | -2.820400 | -2.577300 |
| H | -1.330300 | -3.002500 | -2.830300 |
| H | -3.024800 | -3.108200 | -3.415800 |
| H | -2.604500 | -3.436000 | -1.700600 |
| N | -3.204800 | -0.755100 | 0.932500 |
| N | -2.171100 | 1.714000 | -0.475400 |
| N | -0.939400 | 0.492500 | 1.920100 |
| N | -1.874400 | 1.292800 | 2.493200 |
| N | -3.907700 | 0.208700 | 1.567100 |
| N | -2.977800 | 2.273300 | 0.455700 |
| C | 0.055300 | 0.365800 | 2.801400 |
| H | 0.926200 | -0.234800 | 2.562400 |
| C | -1.459400 | 1.660900 | 3.722300 |
| H | -2.079100 | 2.305500 | 4.332200 |
| C | -3.850700 | -1.908100 | 1.125800 |
| H | -3.436100 | -2.828200 | 0.727100 |
| C | -2.071300 | 2.597500 | -1.475200 |
| H | -1.444100 | 2.377300 | -2.332200 |
| C | -0.226100 | 1.089400 | 3.963500 |
| H | 0.388400 | 1.184600 | 4.846400 |
| C | -2.824300 | 3.740300 | -1.198700 |
| H | -2.940900 | 4.623800 | -1.809200 |
| B | -3.230700 | 1.577200 | 1.812100 |
| C | -3.380200 | 3.488300 | 0.043100 |
| H | -4.028200 | 4.087700 | 0.669400 |
| C | -4.993200 | -0.334700 | 2.150600 |
| H | -5.667800 | 0.284500 | 2.727700 |
| C | -5.006200 | -1.692100 | 1.882200 |
| H | -5.735700 | -2.421500 | 2.202900 |
| H | -3.921100 | 2.272800 | 2.509700 |

**Table S2: DFT Geometry Optimization, Energies and Coordinates for *threo*-7D.**
Electronic Energy: -2695.644006 Hartree
Electronic Energy + Free Energy Correction: -2695.092530 Hartree

| | | | |
|---|---|---|---|
| O | -0.556500 | 0.328100 | 3.092900 |
| N | -0.688300 | 0.185300 | 1.893500 |
| W | -1.227500 | -0.106900 | 0.235500 |
| C | 0.399200 | -1.512200 | -0.449400 |
| C | 0.721600 | -0.130500 | -0.720900 |
| C | 1.973600 | 0.501200 | -0.126500 |
| H | 0.065600 | -2.108100 | -1.303900 |
| H | 0.575400 | 0.181700 | -1.760500 |
| H | 1.767200 | 1.511900 | 0.242900 |
| C | 3.063300 | 0.647000 | -1.246800 |
| N | 2.453000 | -0.257900 | 1.039500 |
| S | 3.390000 | 0.477700 | 2.221800 |
| O | 4.606200 | -0.303000 | 2.406400 |
| O | 3.450500 | 1.892200 | 1.875700 |
| C | 2.428500 | 0.305100 | 3.705100 |
| H | 2.231300 | -0.756100 | 3.877400 |
| H | 3.035600 | 0.716100 | 4.515500 |
| H | 1.483500 | 0.839000 | 3.583900 |
| C | 3.636400 | -0.676200 | -1.674900 |
| C | 4.639400 | -1.321400 | -0.944200 |
| C | 3.100700 | -1.321300 | -2.791800 |
| C | 5.073400 | -2.588000 | -1.318300 |
| H | 5.079900 | -0.831000 | -0.080700 |
| C | 3.531900 | -2.590200 | -3.163100 |
| H | 2.328000 | -0.820800 | -3.376300 |
| C | 4.519600 | -3.229800 | -2.421600 |
| H | 5.853600 | -3.077000 | -0.738900 |
| H | 3.101200 | -3.075100 | -4.037000 |
| H | 4.863300 | -4.221600 | -2.708400 |
| C | 4.091400 | 1.697400 | -0.875600 |
| O | 5.258200 | 1.524400 | -0.629200 |
| O | 3.506500 | 2.912400 | -0.885500 |
| C | 4.331900 | 3.968000 | -0.401400 |
| H | 3.736200 | 4.879200 | -0.479200 |
| H | 4.606000 | 3.774600 | 0.640100 |
| H | 5.242200 | 4.055400 | -1.002500 |
| H | 2.515000 | 1.089200 | -2.092700 |
| C | 1.258300 | -2.251600 | 0.482500 |
| C | 2.234600 | -1.640900 | 1.163000 |
| H | 1.167100 | -3.332600 | 0.572800 |
| H | 2.938200 | -2.167000 | 1.803900 |
| N | -1.287600 | 1.962700 | -0.709900 |
| N | -2.422200 | 2.396000 | -1.320700 |
| N | -2.343800 | -0.569900 | -1.722000 |
| N | -3.368300 | 0.221500 | -2.121500 |
| N | -3.204500 | 0.705500 | 0.898400 |
| N | -4.126200 | 1.231000 | 0.063000 |

| | | | |
|---|---|---|---|
| C | -2.223000 | 3.625800 | -1.832200 |
| C | -0.929000 | 4.017000 | -1.552100 |
| C | -0.386000 | 2.938800 | -0.850300 |
| C | -3.916900 | -0.268100 | -3.247000 |
| C | -3.239900 | -1.419900 | -3.604300 |
| C | -2.262700 | -1.561400 | -2.618300 |
| C | -5.182700 | 1.670100 | 0.773200 |
| C | -4.949100 | 1.415600 | 2.113100 |
| C | -3.684200 | 0.821200 | 2.139800 |
| B | -3.702800 | 1.538500 | -1.386500 |
| H | -4.577200 | 2.123700 | -1.970400 |
| H | -3.023700 | 4.128100 | -2.359700 |
| H | -0.443800 | 4.945400 | -1.814800 |
| H | 0.614200 | 2.831700 | -0.449900 |
| H | -4.746200 | 0.246700 | -3.714700 |
| H | -3.419000 | -2.056700 | -4.458100 |
| H | -1.499300 | -2.323600 | -2.520900 |
| H | -6.016200 | 2.145100 | 0.271800 |
| H | -5.593300 | 1.642700 | 2.950000 |
| H | -3.081600 | 0.489700 | 2.979800 |
| C | -3.952300 | -2.389300 | 1.202300 |
| H | -4.268300 | -3.397000 | 1.496900 |
| H | -4.303400 | -1.667700 | 1.945400 |
| H | -4.410700 | -2.132800 | 0.238500 |
| C | -1.499900 | -2.764000 | 2.677100 |
| H | -1.681700 | -1.937000 | 3.372000 |
| H | -1.969500 | -3.682500 | 3.046600 |
| H | -0.413300 | -2.892100 | 2.609400 |
| P | -2.122900 | -2.307100 | 1.021400 |
| C | -1.866800 | -3.844700 | 0.048100 |
| H | -2.266100 | -4.701500 | 0.603400 |
| H | -2.402200 | -3.766800 | -0.904400 |
| H | -0.806100 | -4.013600 | -0.155400 |

**Figure S55**: H-H Bond Lengths and NOESY Interactions.

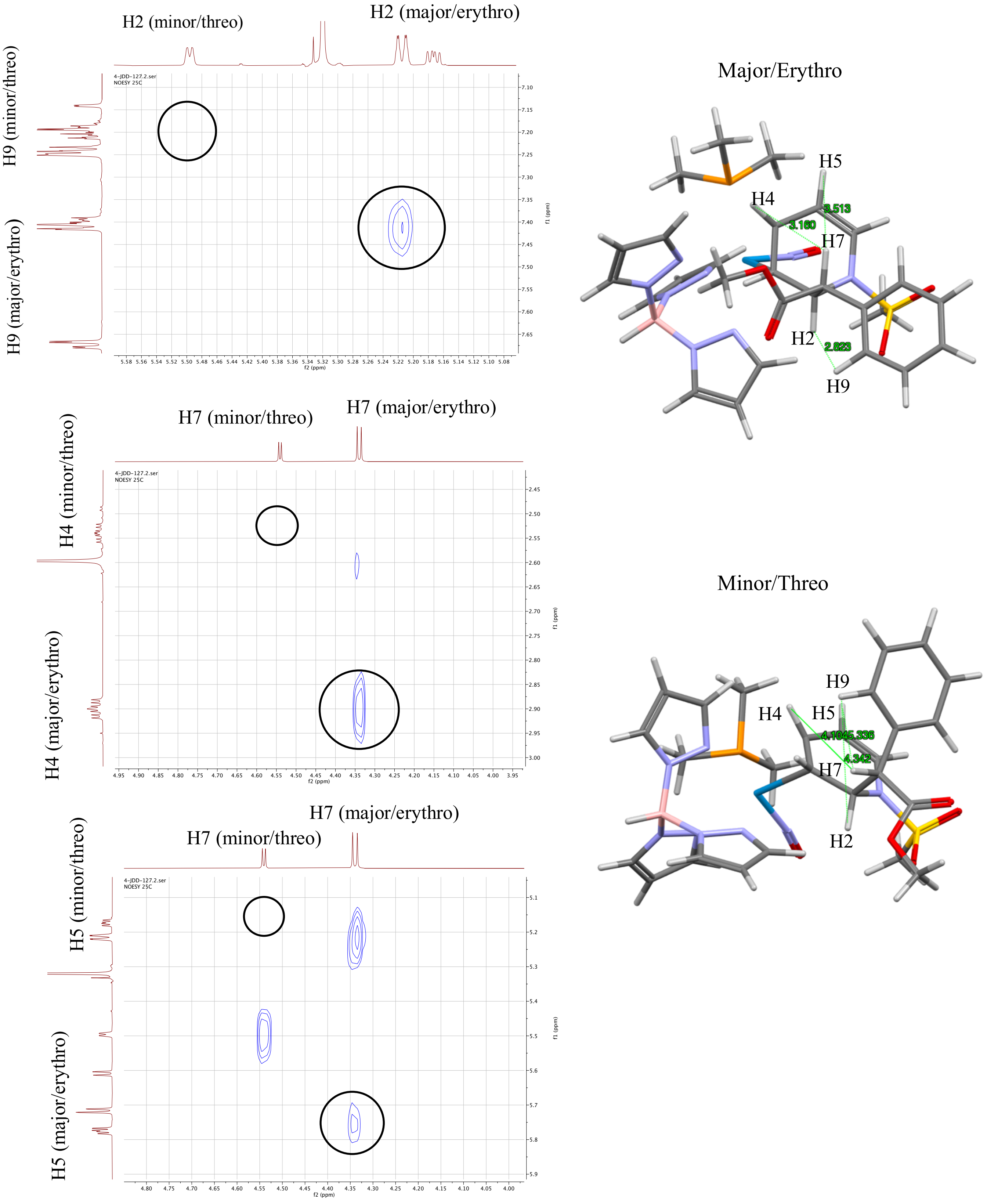

**Chiral HPLC Parameters**:
Equipment: Agilent 1260 Infinity II Flexible Pump (G7104C), Agilent 1260 Vialsampler (G7129C), Agilent 1260 DAD HS (G7117C), Agilent 1260 RID (G7162A), Daicel IC-3 column, 3 um particle size, 4.6 mm x 250 mm

Method: 80/20 Mixed Hexanes (HPLC Grade)/Ethyl Acetate (HPLC Grade) isocratic, 1 ml/min for 35 minutes, 40 °C, 2.50 μL injection volume

DAD: 254 nm, 265 nm with a bandwidth of 4 nm; Reference 390 nm with a bandwidth of 20 nm

**Figure S56**: Chiral HPLC Spectrum for 3:1 ± ***erythro/threo* 21-Ms**

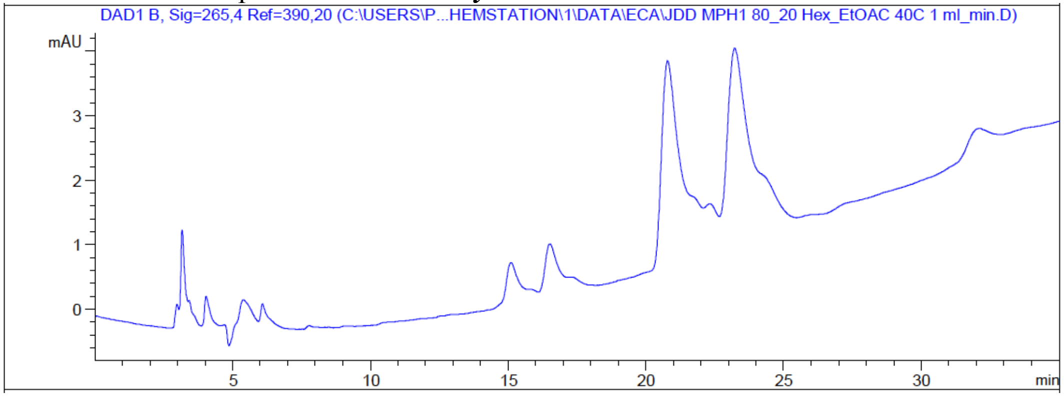


**Figure S57**: Chiral HPLC Spectrum for ± ***erythro* 21-Ms**

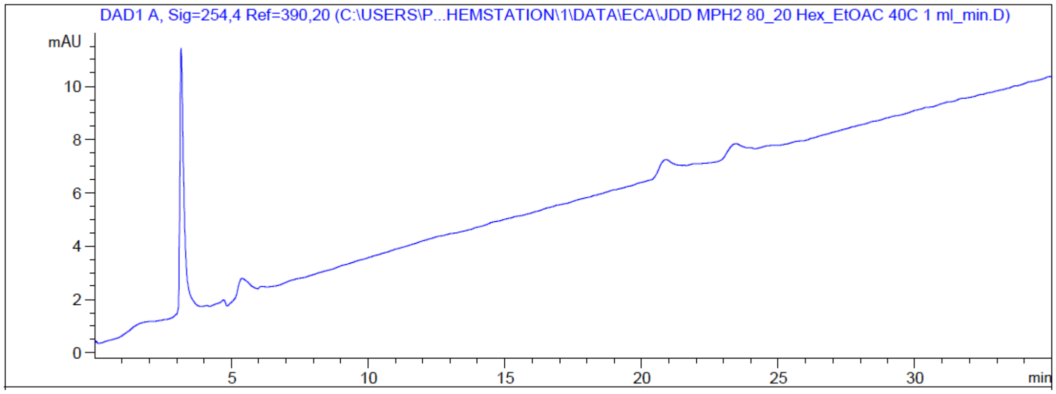


**Figure S58**: Chiral HPLC Spectrum for **(2*S*,7*R*)-21-Ms**

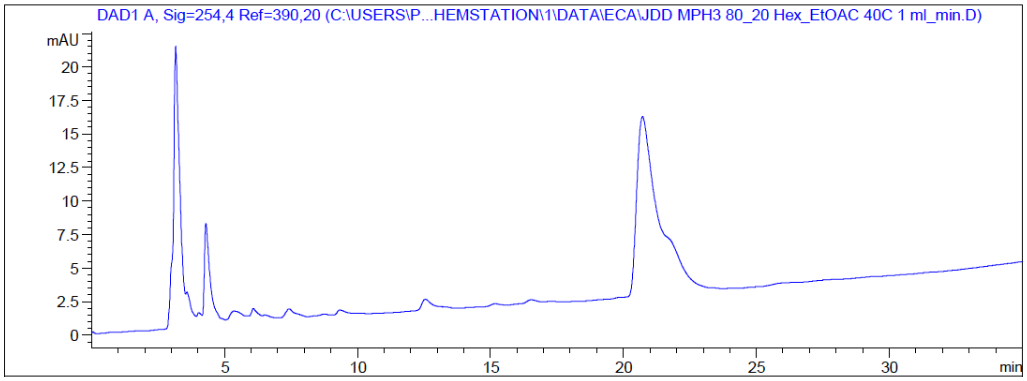

**Crystallography**:

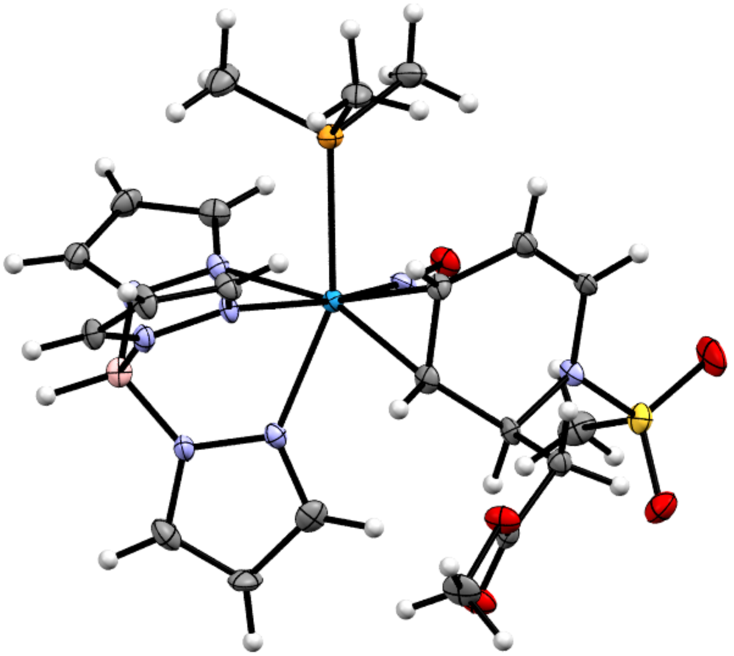

**Figure S59**: ORTEP/ellipsoid diagram of **6D**.

**Table S3**: SC-XRD data for **6D**.

| | | |
|---|---|---|
| CCDC | **Chemical Formula** | **FW (g/mol)** |
| 2256163 | $C_{21}H_{32}BN_8O_5PSW$ | 734.22 |
| T (K) | **λ** (Å) | Crystal size (mm) |
| 100(2) | 0.71073 | 0.083 x0.090 x 0.133 |
| Crystal habit | Crystal system | Space group |
| Yellow block | Monoclinic | P $2_{1/n}$ |
| a (Å) | b (Å) | c (Å) |
| 11.4488(15) | 20.387(3) | 12.8800(18) |
| α (°) | β (°) | γ (°) |
| 90 | 104.213(2) | 2914.3(7) |
| V ($Å^3$) | Z | $\rho_{calc}$ ($g/cm^3$) |
| 2914.3(7) | 2 | 1.770 |
| μ ($mm^{-1}$) | F(000) | θ range (°) |
| 4.229 | 1540 | 1.91 to 28.29 |
| Index ranges | Data/restraints/parameters | Goodness-of-fit on $F^2$ |
| $-15 \leq h \leq 15$<br>$-27 \leq k \leq 26$<br>$-17 \leq l \leq 17$ | 7221 / 0 / 385 | 0.976 |
| $R_1$ [I>2σ(I)] | $wR_2$ [all data] | |
| 0.0361 | 0.0706 | |

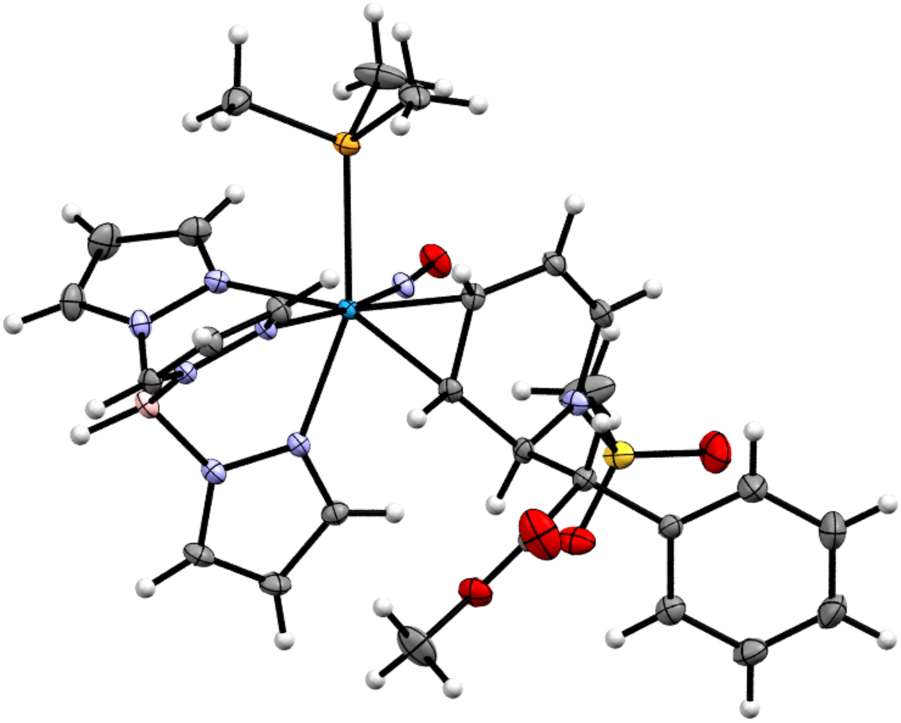

**Figure S60**: ORTEP/ellipsoid diagram of ***erythro* 7D**.

**Table S4**: SC-XRD data for **7D**.

| | | |
|---|---|---|
| CCDC<br>2256164 | Chemical Formula<br>$C_{27}H_{36}BN_8O_5PSW$ | FW (g/mol)<br>810.33 |
| T (K)<br>100(2) | $\lambda$ (Å)<br>0.71073 | Crystal size (mm)<br>0.070 x 0.144 x 0.164 |
| Crystal habit<br>Colorless plate | Crystal system<br>Triclinic | Space group<br>P -1 |
| a (Å)<br>9.8584(12) | b (Å)<br>12.0339(15) | c (Å)<br>14.6597(17) |
| α (°)<br>107.053(4) | β (°)<br>95.521(4) | γ (°)<br>103.067(4) |
| V (Å$^3$)<br>1594.8(3) | Z<br>2 | $\rho_{calc}$ (g/cm$^3$)<br>1.687 |
| μ (mm$^{-1}$)<br>3.787 | F(000)<br>808 | θ range (°)<br>1.48 to 30.57 |
| Index ranges<br>$-14 \le h \le 14$<br>$-17 \le k \le 17$<br>$-20 \le l \le 20$ | Data/restraints/parameters<br>9762 / 0 / 413 | Goodness-of-fit on F$^2$<br>1.025 |
| $R_1$ [I>2σ(I)]<br>0.0221 | $wR_2$ [all data]<br>0.0442 | |

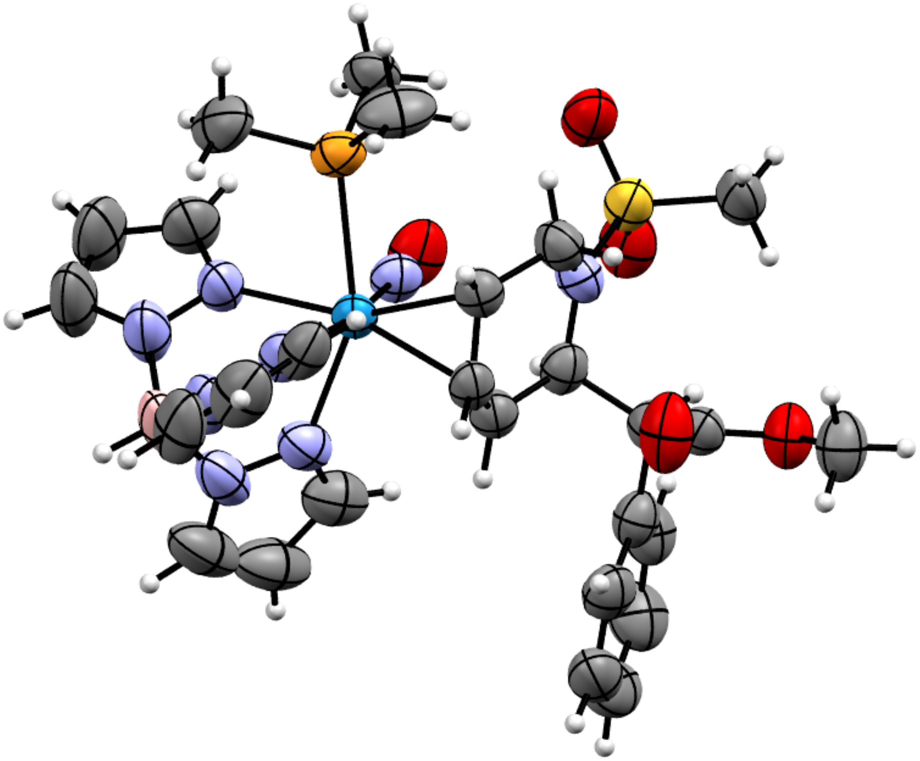

**Figure S61**: ORTEP/ellipsoid diagram of **8**.

**Table S5**: SC-XRD data for **8**.

| | | |
|---|---|---|
| CCDC | Chemical Formula | FW (g/mol) |
| 2256165 | $C_{28}H_{37}BF_3N_8O_8PS_2W$ | 961.52 |
| T (K) | $\lambda$ (Å) | Crystal size (mm) |
| 200(2) | 1.54178 | 0.057 x 0.081 x 0.092 |
| Crystal habit | Crystal system | Space group |
| Colorless plate-like | monoclinic | P $2_{1/c}$ |
| a (Å) | b (Å) | c (Å) |
| 15.1929(6) | 16.8559(7) | 15.1263(6) |
| α (°) | β (°) | γ (°) |
| 90 | 106.0660(10) | 90 |
| V ($Å^3$) | Z | $\rho_{calc}$ ($g/cm^3$) |
| 3722.4(3) | 4 | 1.716 |
| μ ($mm^{-1}$) | F(000) | θ range (°) |
| 7.851 | 1914 | 3.03 to 68.420 |
| Index ranges | Data/restraints/parameters | Goodness-of-fit on $F^2$ |
| $-18 \leq h \leq 17$<br>$0 \leq k \leq 20$<br>$0 \leq l \leq 18$ | 6825 / 688 / 622 | 1.061 |
| $R_1$ [I>2σ(I)] | $wR_2$ [all data] | |
| 0.0402 | 0.1196 | |

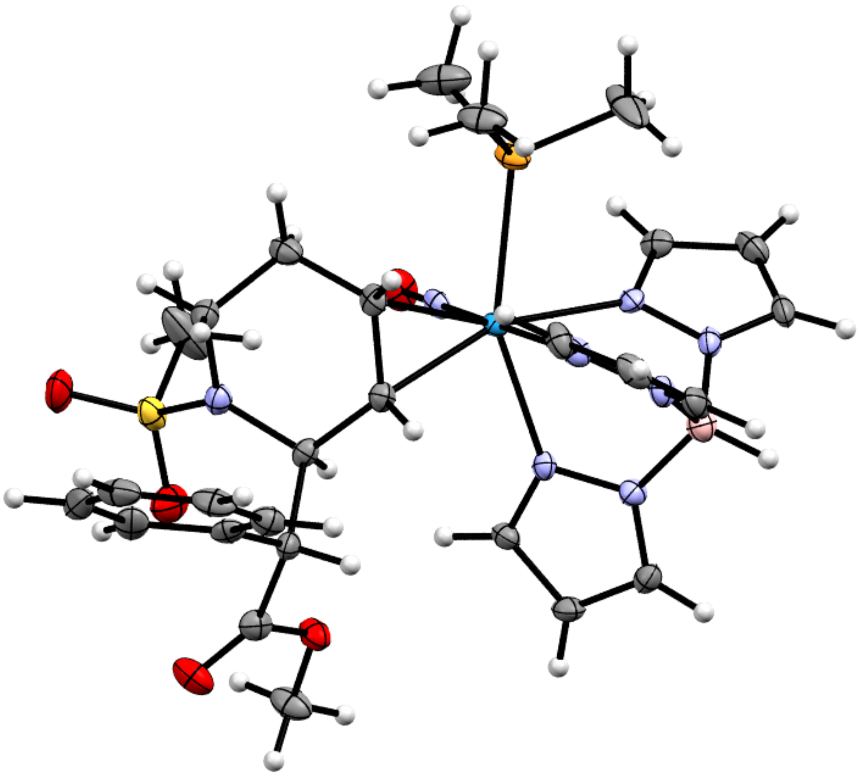

**Figure S62**: ORTEP/ellipsoid diagram of **9D**.

**Table S6**: SC-XRD data for **9D**.

| | | |
|---|---|---|
| CCDC<br>2256166 | Chemical Formula<br>$C_{27}H_{38}BN_8O_5PSW$ | FW (g/mol)<br>812.34 |
| T (K)<br>100(2) | **λ** (Å)<br>0.71073 | Crystal size (mm)<br>0.055 x 0.065 x 0.073 |
| Crystal habit<br>Colorless plate | Crystal system<br>triclinic | Space group<br>P -1 |
| a (Å)<br>10.8643(5) | b (Å)<br>12.0141(6) | c (Å)<br>13.3520(7) |
| α (°)<br>92.992(2) | β (°)<br>93.154(2) | γ (°)<br>113.706(2) |
| V ($Å^3$)<br>1588.02(14) | Z<br>2 | $\rho_{calc}$ ($g/cm^3$)<br>1.699 |
| μ ($mm^{-1}$)<br>3.804 | F(000)<br>812 | θ range (°)<br>2.05 to 27.89 |
| Index ranges<br>$-14 \leq h \leq 12$<br>$-15 \leq k \leq 15$<br>$-17 \leq l \leq 17$ | Data/restraints/parameters<br>7570 / 0 / 413 | Goodness-of-fit on $F^2$<br>1.055 |
| $R_1$ [I>2σ(I)]<br>0.0396 | $wR_2$ [all data]<br>0.0792 | |

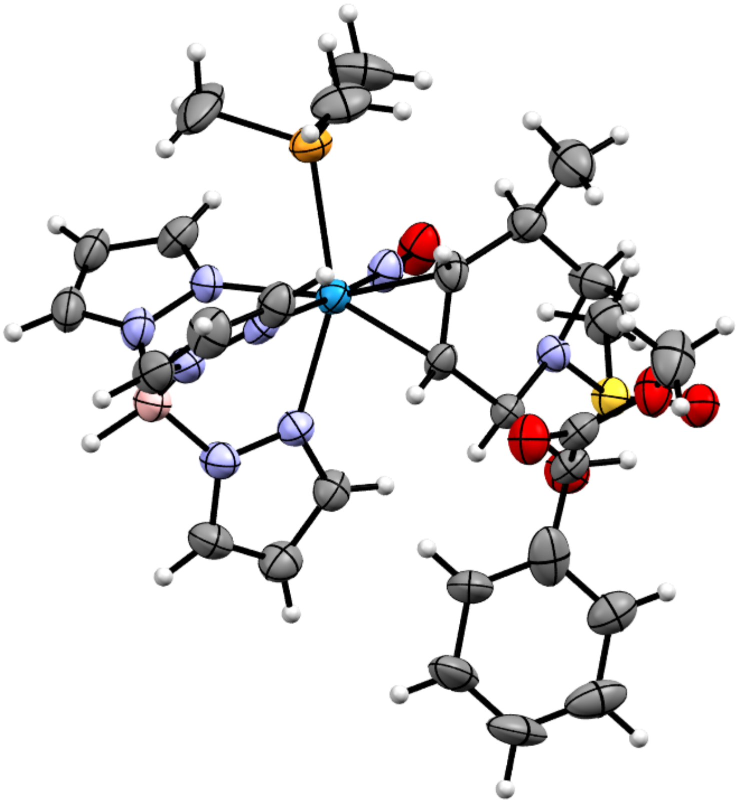

**Figure S63**: ORTEP/ellipsoid diagram of **13D**.

**Table S7**: SC-XRD data for **13D**.

| | | |
|---|---|---|
| CCDC<br>2256167 | Chemical Formula<br>$C_{28}H_{40}BN_8O_5PSW$ | FW (g/mol)<br>826.37 |
| T (K)<br>100(2) | **λ** (Å)<br>1.54178 | Crystal size (mm)<br>0.037 x 0.054 x 0.070 |
| Crystal habit<br>clear yellow needle | Crystal system<br>triclinic | Space group<br>P -1 |
| a (Å)<br>8.6828(4) | b (Å)<br>12.4253(5) | c (Å)<br>16.5767(8) |
| α (°)<br>78.170(3) | β (°)<br>77.152(4) | γ (°)<br>71.616(4) |
| V ($Å^3$)<br>1636.96(14) | Z<br>2 | $\rho_{calc}$ ($g/cm^3$)<br>1.677 |
| μ ($mm^{-1}$)<br>8.020 | F(000)<br>828 | θ range (°)<br>2.76 to 67.72 |
| Index ranges<br>$-10 \leq h \leq 10$<br>$-14 \leq k \leq 14$<br>$-19 \leq l \leq 19$ | Data/restraints/parameters<br>5819 / 162 / 454 | Goodness-of-fit on $F^2$<br>0.997 |
| $R_1$ [I>2σ(I)]<br>0.0457 | $wR_2$ [all data]<br>0.1192 | |

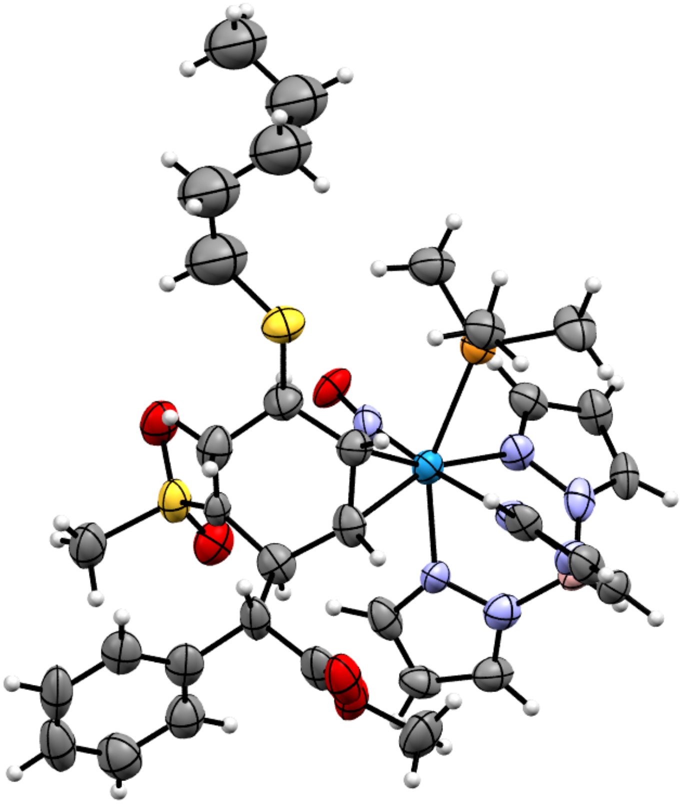

**Figure S64**: ORTEP/ellipsoid diagram of **19D**.

**Table S8**: SC-XRD data for **19D**.

| CCDC | Chemical Formula | FW (g/mol) |
|---|---|---|
| 2256168 | $C_{32}H_{48}BN_8O_5PS_2W$ | 914.53 |
| T (K) | **λ** (Å) | Crystal size (mm) |
| 100(2) | 0.71073 | 0.043 x 0.047 x 0.071 |
| Crystal habit | Crystal system | Space group |
| translucent colorless needle | orthorhombic | P $2_12_12_1$ |
| a (Å) | b (Å) | c (Å) |
| 10.6315(5) | 20.7318(12) | 21.6035(12) |
| α (°) | β (°) | γ (°) |
| 90 | 90 | 90 |
| V ($Å^3$) | Z | $\rho_{calc}$ ($g/cm^3$) |
| 4761.6(4) | 4 | 1.273 |
| μ ($mm^{-1}$) | F(000) | θ range (°) |
| 2.586 | 1844 | 1.97 to 25.69 |
| Index ranges | Data/restraints/parameters | Goodness-of-fit on $F^2$ |
| $-12 \leq h \leq 12$<br>$-25 \leq k \leq 25$<br>$-26 \leq l \leq 26$ | 9030 / 28 / 459 | 1.058 |
| $R_1$ [I>2σ(I)] | $wR_2$ [all data] | |
| 0.0422 | 0.1021 | |

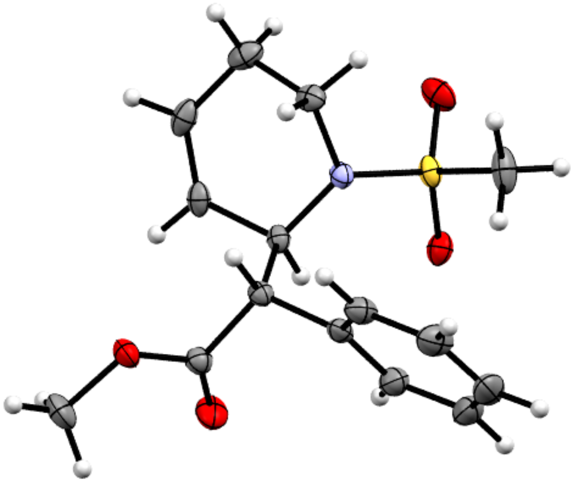

**Figure S65**: ORTEP/ellipsoid diagram of **21-Ms**.

**Table S9**: SC-XRD data for **21-Ms**.

| | | |
|---|---|---|
| CCDC | Chemical Formula | FW (g/mol) |
| 2256169 | $C_{15}H_{19}NO_4S$ | 309.37 |
| T (K) | $\lambda$ (Å) | Crystal size (mm) |
| 100(2) | 1.54178 | 0.100 x 0.222 x 0.438 |
| Crystal habit | Crystal system | Space group |
| Colorless plate | monoclinic | P $2_1$/c |
| a (Å) | b (Å) | c (Å) |
| 13.4105(4) | 8.4916(3) | 13.0180(4) |
| α (°) | β (°) | γ (°) |
| 90 | 94.6520(10) | 90 |
| V (Å$^3$) | Z | $\rho_{calc}$ (g/cm$^3$) |
| 1477.56(8) | 4 | 1.391 |
| μ (mm$^{-1}$) | F(000) | θ range (°) |
| 2.090 | 656 | 3.31 to 72.31 |
| Index ranges | Data/restraints/parameters | Goodness-of-fit on F$^2$ |
| $-16 \le h \le 16$<br>$-10 \le k \le 10$<br>$-16 \le l \le 16$ | 2912 / 0 / 192 | 1.052 |
| $R_1$ [I>2σ(I)] | $wR_2$ [all data] | |
| 0.0297 | 0.0797 | |

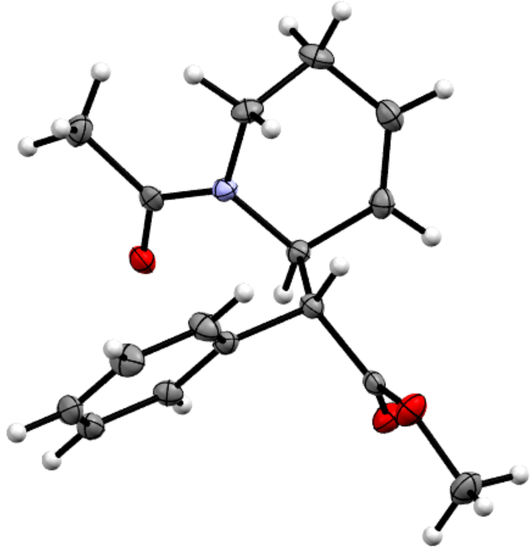

**Figure S66**: ORTEP/ellipsoid diagram of **21-Ac**.

**Table S10**: SC-XRD data for **21-Ac**.

| | | |
|---|---|---|
| CCDC | Chemical Formula | FW (g/mol) |
| 2260260 | $C_{16}H_{19}NO_3$ | 273.32 |
| T (K) | $\lambda$ (Å) | Crystal size (mm) |
| 100(2) | 1.54178 | 0.076 x 0.081 x 0.422 |
| Crystal habit | Crystal system | Space group |
| colorless rod | monoclinic | C 2/c |
| a (Å) | b (Å) | c (Å) |
| 22.6503(8) | 5.8701(2) | 21.6027(8) |
| α (°) | β (°) | γ (°) |
| 90 | 96.692(3) | 90 |
| V (Å$^3$) | Z | $\rho_{calc}$ (g/cm$^3$) |
| 2852.72(18) | 8 | 1.273 |
| μ (mm$^{-1}$) | F(000) | θ range (°) |
| 0.711 | 1168 | 3.93 to 68.30 |
| Index ranges | Data/restraints/parameters | Goodness-of-fit on F$^2$ |
| $-27 \leq h \leq 27$<br>$-7 \leq k \leq 7$<br>$-23 \leq l \leq 26$ | 2596 / 0 / 183 | 1.047 |
| $R_1$ [I>2σ(I)] | $wR_2$ [all data] | |
| 0.0382 | 0.1003 | |

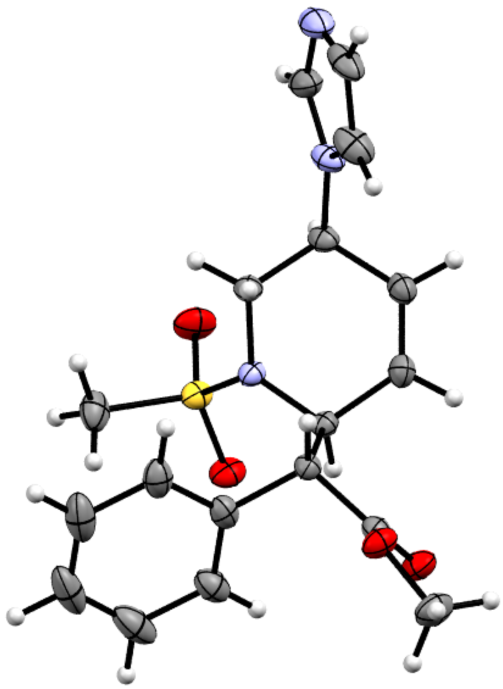

**Figure S67**: ORTEP/ellipsoid diagram of **22**.

**Table S11**: SC-XRD data for **22**.

| | | |
|---|---|---|
| CCDC<br>2256170 | Chemical Formula<br>$C_{18}H_{21}N_3O_4S$ | FW (g/mol)<br>375.44 |
| T (K)<br>100.0 | $\lambda$ (Å)<br>0.71073 | Crystal size (mm)<br>0.163 × 0.1 × 0.076 |
| Crystal habit<br>yellow plate | Crystal system<br>monoclinic | Space group<br>$P2_1/c$ |
| a (Å)<br>10.9884(3) | b (Å)<br>13.2829(4) | c (Å)<br>12.9404(3) |
| α (°)<br>90 | β (°)<br>91.6820(10) | γ (°)<br>90 |
| V ($Å^3$)<br>1887.94(9) | Z<br>4 | $\rho_{calc}$ ($g/cm^3$)<br>1.321 |
| μ ($mm^{-1}$)<br>0.199 | F(000)<br>792.0 | θ range (°)<br>4.396 to 56.574 |
| Index ranges<br>$-14 \leq h \leq 14$<br>$-17 \leq k \leq 17$<br>$-15 \leq l \leq 17$ | Data/restraints/parameters<br>4683 / 0 / 237 | Goodness-of-fit on $F^2$<br>1.050 |
| $R_1$ [I>2σ(I)]<br>0.0466 | $wR_2$ [all data]<br>0.1331 | |

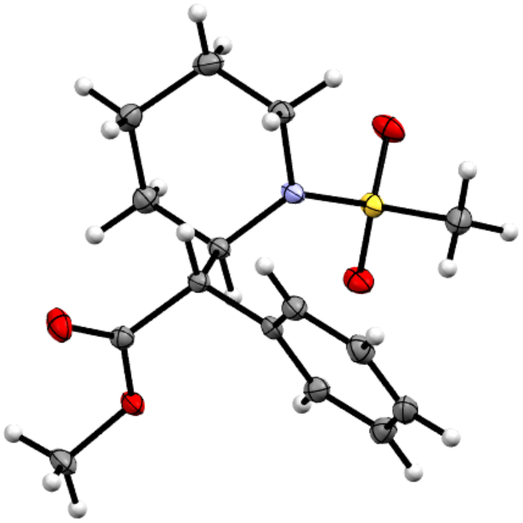

**Figure S68**: ORTEP/ellipsoid diagram of **26**.

**Table S12**: SC-XRD data for **26**.

| | | |
|---|---|---|
| CCDC | Chemical Formula | FW (g/mol) |
| 2256171 | $C_{15}H_{21}NO_4S$ | 311.39 |
| T (K) | $\boldsymbol{\lambda}$ (Å) | Crystal size (mm) |
| 100(2) | 0.71073 | 0.096 x 0.155 x 0.261 |
| Crystal habit | Crystal system | Space group |
| colorless block | triclinic | P -1 |
| a (Å) | b (Å) | c (Å) |
| 8.7149(3) | 9.2516(3) | 10.3319(4) |
| α (°) | β (°) | γ (°) |
| 89.9360(10) | 67.2660(10) | 80.0590(10) |
| V ($Å^3$) | Z | $\rho_{calc}$ ($g/cm^3$) |
| 754.80(5) | 2 | 1.370 |
| μ ($mm^{-1}$) | F(000) | θ range (°) |
| 0.230 | 332 | 2.14 to 29.59 |
| Index ranges | Data/restraints/parameters | Goodness-of-fit on $F^2$ |
| $-12 \leq h \leq 12$<br>$-12 \leq k \leq 12$<br>$-14 \leq l \leq 14$ | 4232 / 0 / 192 | 1.024 |
| $R_1$ [I>2σ(I)] | $wR_2$ [all data] | |
| 0.0326 | 0.0851 | |

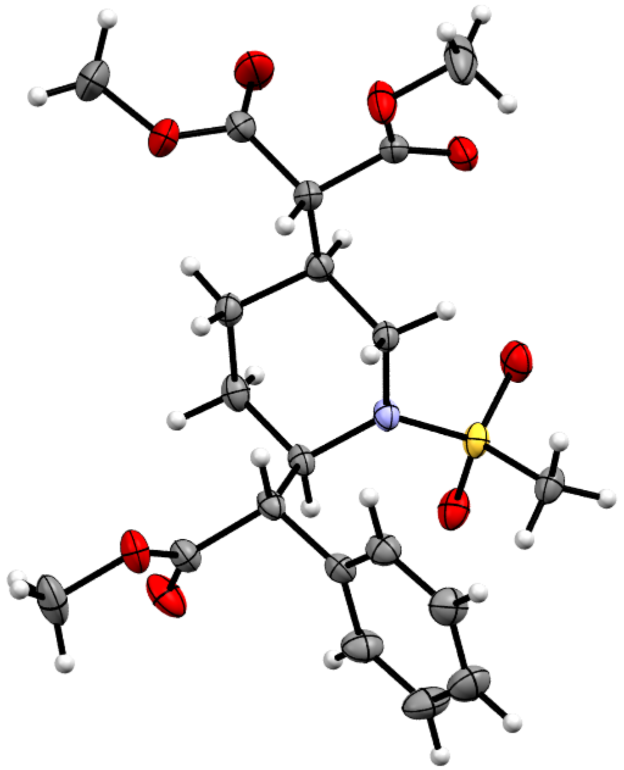

**Figure S69**: ORTEP/ellipsoid diagram of **27**.

**Table S13**: SC-XRD data for **27**.

| | | |
|---|---|---|
| CCDC<br>2256172 | Chemical Formula<br>$C_{20}H_{27}NO_8S$ | FW (g/mol)<br>441.48 |
| T (K)<br>100(2) | $\boldsymbol{\lambda}$ (Å)<br>1.54178 | Crystal size (mm)<br>0.027 x 0.049 x 0.249 |
| Crystal habit<br>colorless needle | Crystal system<br>monoclinic | Space group<br>I a |
| a (Å)<br>13.1713(3) | b (Å)<br>8.4679(2) | c (Å)<br>19.5697(7) |
| α (°)<br>90 | β (°)<br>90.4740(10) | γ (°)<br>90 |
| V (Å$^3$)<br>2182.60(11) | Z<br>4 | $\rho_{calc}$ (g/cm$^3$)<br>1.344 |
| μ (mm$^{-1}$)<br>1.721 | F(000)<br>936 | θ range (°)<br>6.21 to 72.12 |
| Index ranges<br>$-16 \leq h \leq 14$<br>$-10 \leq k \leq 10$<br>$-23 \leq l \leq 24$ | Data/restraints/parameters<br>4215 / 2 / 275 | Goodness-of-fit on F$^2$<br>1.035 |
| $R_1$ [I>2σ(I)]<br>0.0306 | $wR_2$ [all data]<br>0.0812 | |

References:

1. Wilde, J. H. Synthetic Applications of Molybdenum and Tungsten Dearomatization Agents. University of Virginia, 2020. Pages 145-156.
