# Supplementary material for: The Tungsten-Promoted Synthesis of Piperidyl-Modified erythro-Methylphenidate Derivatives
Source: ACS Cent Sci. 2023 Aug 30;9(9):1775–83. doi: 10.1021/acscentsci.3c00556 (PMC10540299; doi:10.1021/acscentsci.3c00556)
Supplement: Supplementary file 3 — oc3c00556_si_003.pdf [file oc3c00556_si_003.pdf]

80

DHP S,S Conformation 1

O	-0.556500	0.328100	3.092900
N	-0.688300	0.185300	1.893500
W	-1.227500	-0.106900	0.235500
C	0.399200	-1.512200	-0.449400
C	0.721600	-0.130500	-0.720900
C	1.973600	0.501200	-0.126500
H	0.065600	-2.108100	-1.303900
H	0.575400	0.181700	-1.760500
H	1.767200	1.511900	0.242900
C	3.063300	0.647000	-1.246800
N	2.453000	-0.257900	1.039500
S	3.390000	0.477700	2.221800
O	4.606200	-0.303000	2.406400
O	3.450500	1.892200	1.875700
C	2.428500	0.305100	3.705100
H	2.231300	-0.756100	3.877400
H	3.035600	0.716100	4.515500
H	1.483500	0.839000	3.583900
C	3.636400	-0.676200	-1.674900
C	4.639400	-1.321400	-0.944200
C	3.100700	-1.321300	-2.791800
C	5.073400	-2.588000	-1.318300
H	5.079900	-0.831000	-0.080700
C	3.531900	-2.590200	-3.163100
H	2.328000	-0.820800	-3.376300
C	4.519600	-3.229800	-2.421600
H	5.853600	-3.077000	-0.738900
H	3.101200	-3.075100	-4.037000
H	4.863300	-4.221600	-2.708400
C	4.091400	1.697400	-0.875600
O	5.258200	1.524400	-0.629200
O	3.506500	2.912400	-0.885500
C	4.331900	3.968000	-0.401400
H	3.736200	4.879200	-0.479200
H	4.606000	3.774600	0.640100
H	5.242200	4.055400	-1.002500
H	2.515000	1.089200	-2.092700
C	1.258300	-2.251600	0.482500
C	2.234600	-1.640900	1.163000
H	1.167100	-3.332600	0.572800
H	2.938200	-2.167000	1.803900
N	-1.287600	1.962700	-0.709900
N	-2.422200	2.396000	-1.320700
N	-2.343800	-0.569900	-1.722000
N	-3.368300	0.221500	-2.121500
N	-3.204500	0.705500	0.898400
N	-4.126200	1.231000	0.063000
C	-2.223000	3.625800	-1.832200
C	-0.929000	4.017000	-1.552100
C	-0.386000	2.938800	-0.850300
C	-3.916900	-0.268100	-3.247000
C	-3.239900	-1.419900	-3.604300

C	-2.262700	-1.561400	-2.618300
C	-5.182700	1.670100	0.773200
C	-4.949100	1.415600	2.113100
C	-3.684200	0.821200	2.139800
B	-3.702800	1.538500	-1.386500
H	-4.577200	2.123700	-1.970400
H	-3.023700	4.128100	-2.359700
H	-0.443800	4.945400	-1.814800
H	0.614200	2.831700	-0.449900
H	-4.746200	0.246700	-3.714700
H	-3.419000	-2.056700	-4.458100
H	-1.499300	-2.323600	-2.520900
H	-6.016200	2.145100	0.271800
H	-5.593300	1.642700	2.950000
H	-3.081600	0.489700	2.979800
C	-3.952300	-2.389300	1.202300
H	-4.268300	-3.397000	1.496900
H	-4.303400	-1.667700	1.945400
H	-4.410700	-2.132800	0.238500
C	-1.499900	-2.764000	2.677100
H	-1.681700	-1.937000	3.372000
H	-1.969500	-3.682500	3.046600
H	-0.413300	-2.892100	2.609400
P	-2.122900	-2.307100	1.021400
C	-1.866800	-3.844700	0.048100
H	-2.266100	-4.701500	0.603400
H	-2.402200	-3.766800	-0.904400
H	-0.806100	-4.013600	-0.155400
