# Supplementary material for: The Tungsten-Promoted Synthesis of Piperidyl-Modified erythro-Methylphenidate Derivatives
Source: ACS Cent Sci. 2023 Aug 30;9(9):1775–83. doi: 10.1021/acscentsci.3c00556 (PMC10540299; doi:10.1021/acscentsci.3c00556)
Supplement: Supplementary file 4 — oc3c00556_si_004.pdf [file oc3c00556_si_004.pdf]

```
80
mo_harman_mne_nb1_197_0m
W      -1.277300   -0.358800   -0.127400
S       3.133200   -2.164600    0.838600
O       2.402400    3.114100   -1.759800
O       3.555700   -1.287700    1.921500
O      -0.812300   -3.271500    0.209300
O       2.635700    3.087700    0.473200
O       4.080800   -2.982800    0.095900
N      -0.871400   -2.065200    0.113500
N       2.298600   -1.207900   -0.256700
C       0.651400    0.573900   -0.489800
C       0.228400    0.027500   -1.762900
H      -0.113300    0.720500   -2.537900
C       0.987300   -1.135400   -2.253000
H       0.785800   -1.536700   -3.245700
C       2.041200    0.209100   -0.012300
H       2.184200    0.408700    1.055800
C       1.935100   -1.733100   -1.511100
H       2.494700   -2.608600   -1.830900
C       3.089800    1.073700   -0.787400
H       2.969000    0.837000   -1.852100
C       4.511300    0.775800   -0.376900
C       2.711200    2.518500   -0.592800
C       5.255800   -0.117200   -1.147900
H       4.811100   -0.543800   -2.047000
C       6.364900    0.939900    1.162800
H       6.794700    1.353000    2.072900
C       1.897900   -3.265800    1.488300
H       2.418700   -3.970700    2.141200
H       1.397000   -3.781400    0.666400
H       1.163100   -2.683100    2.049300
C       6.539700   -0.488000   -0.764800
H       7.099500   -1.198500   -1.368400
C       1.950200    4.457200   -1.636400
H       2.704700    5.082200   -1.149100
H       1.031500    4.500000   -1.039300
H       1.761700    4.810000   -2.651600
C       5.078400    1.302700    0.785800
H       4.498100    1.989300    1.397900
C       7.098100    0.042300    0.392200
H       8.101200   -0.247700    0.697200
H       0.457400    1.644000   -0.352400
P      -2.574400   -1.049400   -2.165200
C      -4.395800   -0.820100   -2.039600
H      -4.806900   -1.422700   -1.224600
H      -4.890100   -1.094100   -2.978900
H      -4.600100    0.234700   -1.816200
C      -2.286100   -0.253900   -3.796200
H      -2.493400    0.819800   -3.730900
H      -2.956700   -0.694700   -4.543000
H      -1.251100   -0.394100   -4.120500
C      -2.380700   -2.820400   -2.577300
H      -1.330300   -3.002500   -2.830300
```

H	-3.024800	-3.108200	-3.415800
H	-2.604500	-3.436000	-1.700600
N	-3.204800	-0.755100	0.932500
N	-2.171100	1.714000	-0.475400
N	-0.939400	0.492500	1.920100
N	-1.874400	1.292800	2.493200
N	-3.907700	0.208700	1.567100
N	-2.977800	2.273300	0.455700
C	0.055300	0.365800	2.801400
H	0.926200	-0.234800	2.562400
C	-1.459400	1.660900	3.722300
H	-2.079100	2.305500	4.332200
C	-3.850700	-1.908100	1.125800
H	-3.436100	-2.828200	0.727100
C	-2.071300	2.597500	-1.475200
H	-1.444100	2.377300	-2.332200
C	-0.226100	1.089400	3.963500
H	0.388400	1.184600	4.846400
C	-2.824300	3.740300	-1.198700
H	-2.940900	4.623800	-1.809200
B	-3.230700	1.577200	1.812100
C	-3.380200	3.488300	0.043100
H	-4.028200	4.087700	0.669400
C	-4.993200	-0.334700	2.150600
H	-5.667800	0.284500	2.727700
C	-5.006200	-1.692100	1.882200
H	-5.735700	-2.421500	2.202900
H	-3.921100	2.272800	2.509700
