# Supplementary material for: The Tungsten-Promoted Synthesis of Piperidyl-Modified erythro-Methylphenidate Derivatives
Source: ACS Cent Sci. 2023 Aug 30;9(9):1775–83. doi: 10.1021/acscentsci.3c00556 (PMC10540299; doi:10.1021/acscentsci.3c00556)
Supplement: Supplementary file 5 — oc3c00556_si_005.pdf [file oc3c00556_si_005.pdf]

oc-2023-005568.R1

Name: Peer Review Information for "The Tungsten-Promoted Synthesis of Piperidyl-Modified erythro-Methylphenidate Derivatives"

First Round of Reviewer Comments

Reviewer: 1

Comments to the Author

This is a very nice piece of work which is beautifully presented. It describes the use of a sterically hindered low-valent tungsten centre as a binding and activation site for protected pyridines and their subsequent conversion into piperidenes via nucleophilic attack. The latter are highly selective and result in formation of otherwise inaccessible or difficult to prepare molecules that have relevance to the pharmaceutical industry. The ESI contains full characterizing data.

I can recommend publication following some very small changes;

(i) please check ref 4 - title suggests it relates to obesity treatment rather than cocaine addition as stated

(ii) Results and discussion - although very well-presented, the authors should consider some sub-headings to direct the reader as otherwise its quite a "deep" dive

(iii) ref 26 is from a 2020 PhD thesis. It seems to be the only reference supporting a key point here - namely the selective attack at C2 vs C4. This always unnerves me as its nearly impossible to track back and has not been peer reviewed. Are there other references that support this statement that can also be included? If not then can some information be put into the ESI to support the statement e.g. relevant section of the thesis?

(iv) "consecutive triturations" - I can see this being a nightmare to reproduce. I didn't directly check the ESI but hope that the instructions given are very clear as the statement is "woolly"

Reviewer: 2

Comments to the Author

The authors utilize a stoichiometric tungsten-based dearomatizing agent to convert pyridine into a variety of unconventionally substituted derivatives of the stimulant methylphenidate. This work builds on prior studies exploring the synthesis of the η^2-pyridine complex and its reaction with nucleophiles. Notable advances include the development of preparative methods to control the ratio of coordination diastereomers and expanding the scope of competent nucleophiles.

By varying the pyridine protecting group, the authors can enrich selectivity for a single coordination diastereomer through isomerization. This enables the later enantioenriched synthesis of a piperidinyl-substituted Ritalin derivative using established methodology in combination with the synthetic sequence developed in this work. Detailed study of the key Reformatsky reaction and subsequent protonation and nucleophilic addition steps by XRD, NOESY, and DFT demonstrate a thorough understanding of each step in the synthetic sequence. A diverse set of hydride, carbon, nitrogen, and sulfur nucleophiles are employed to prepare >10 tungsten-coordinated Ritalin derivatives.

An assessment of the biological activity for these unique derivatives would be a welcome addition to the manuscript but may lie outside the scope of the present publication. Previous studies on tungsten-mediated pyridine dearomatization establish that pyridinium salts, besides the pyridine-borane complex, promote oxidative decomposition of the tungsten complex. However, a control experiment treating the WTp(NO)(PMe3)-benzene complex with mesyl pyridinium could serve to justify the extra deprotection/protection steps.

While the manuscript and SI are well-written, there are a few corrections that should be taken into consideration prior to publication.

1. The phrase “cocaine agonist” features an unusual use of the word “agonist”, since cocaine is not a biological receptor. While reference 5 suggests that methylphenidate could be classified as an “agonist” medication relative to cocaine since they share similar pharmacodynamic mechanisms of action, consider rephrasing this in the current manuscript.

2. The phrase “however, to confirm the chiral identity of the bulk material” features a misleading instance of the word “chiral”. The NOESY, DFT, and XRD experiments confirm the predominant diastereomer present, but do not have anything to do with the chirality of the material because the complex is a racemic mixture.

3. Product yields of isolated compounds should be included in Figures 3 and 9.

4. More detailed reaction conditions should be provided in a caption for Table 1 and in Figure 7.

Author's Response to Peer Review Comments:

Sunday, July 16, 2023

Dear Professor Editor,

Please find attached the revised manuscript entitled "The Tungsten-Promoted Synthesis of Piperidyl-Modified *erythro*-Methylphenidate Derivatives" to be considered for publication in ACS Central Science.

We are delighted that you and the reviewers found our manuscript worthy of publication in ACS Central Science. As requested, I have attached a copy of the revised manuscript with changes highlighted. Here is our point by point response:

Formatting Needs:
AU EMAIL: Please include the email address of the corresponding author on the first page of the manuscript, and the Supporting Information if submitted, with an asterisk next to their name in the author list. Please be sure to label "email." Done for both main text and SI.
TOC MISSING: Provide a TOC image per journal guidelines (3.25 in. × 1.75 in. (8.25 cm × 4.45 cm) ; on the last page of the Manuscript) with the heading "TOC Graphic" above the graphic. Make sure to designate the file as "Graphic for Manuscript." TOC added
SYNOPSIS MISSING: The synopsis should be no more than 200 characters (including spaces) and should reasonably correlate with the TOC graphic. The synopsis is intended to explain the importance of the article to a broader readership across the sciences. Please place your synopsis in the manuscript file after the TOC graphic. Synopsis added

Reviewer 1:
I can recommend publication following some very small changes;
(i) please check ref 4 - title suggests it relates to obesity treatment rather than cocaine addition as stated Ref. 4 has been replaced with a more appropriate reference.
(ii) Results and discussion - although very well-presented, the authors should consider some sub-headings to direct the reader as otherwise its quite a "deep" dive Subheadings have been added.
(iii) ref 26 is from a 2020 PhD thesis. It seems to be the only reference supporting a key point here - namely the selective attack at C2 vs C4. This always unnerves me as its nearly impossible to track back and has not been peer reviewed. Are there other references that support this statement that can also be included? If not then can some information be put into the ESI to support the statement e.g. relevant section of the thesis? An additional reference has been cited (25) that definitively addresses the C2 vs C4 issue. In addition, the section in the thesis has been specified with page numbers in the SI.
(iv) "consecutive triturations" - I can see this being a nightmare to reproduce. I didn't directly check the ESI but hope that the instructions given are very clear as the statement is "woolly" More detail has been added to the SI. Further, the statement in the manuscript has been expanded.

Reviewer 2:
While the manuscript and SI are well-written, there are a few corrections that should be taken into consideration prior to publication.
1. The phrase "cocaine agonist" features an unusual use of the word "agonist", since cocaine is not a biological receptor. While reference 5 suggests that methylphenidate could be classified as an "agonist" medication relative to cocaine since they share similar pharmacodynamic mechanisms of action, consider

rephrasing this in the current manuscript. This statement has been rephrased. Thank you for alerting us to this error.

2. The phrase “however, to confirm the chiral identity of the bulk material” features a misleading instance of the word “chiral”. The NOESY, DFT, and XRD experiments confirm the predominant diastereomer present, but do not have anything to do with the chirality of the material because the complex is a racemic mixture. The referee is correct. The word “chiral” was replaced “with stereochemical”

3. Product yields of isolated compounds should be included in Figures 3 and 9. Yields are now included.

4. More detailed reaction conditions should be provided in a caption for Table 1 and in Figure 7. Reaction conditions are now included in caption.

We have elected to not submit a journal cover because of lack of resources, but of course we would welcome any elaboration of our TOC graphic into a cover design by ACS artists.

I Thank you for your further consideration of our manuscript.

Sincerely yours,

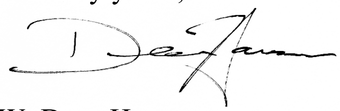

W. Dean Harman
William R. Kenan Jr. Professor of Chemistry
